# The Tracking of Moist Habitats Allowed *Aiphanes* (Arecaceae) to Cover the Elevation Gradient of the Northern Andes

**DOI:** 10.3389/fpls.2022.881879

**Published:** 2022-06-27

**Authors:** María José Sanín, Finn Borchsenius, Margot Paris, Sara Carvalho-Madrigal, Andrés Camilo Gómez Hoyos, Agustín Cardona, Natalia Arcila Marín, Yerson Ospina, Saúl E. Hoyos-Gómez, Héctor Favio Manrique, Rodrigo Bernal

**Affiliations:** ^1^Facultad de Ciencias y Biotecnología, Universidad CES, Medellín, Colombia; ^2^School of Mathematical and Natural Sciences, Arizona State University, Tempe, AZ, United States; ^3^Departamento de Procesos y Energía, Universidad Nacional de Colombia, Medellín, Colombia; ^4^Faculty of Technical Sciences, Aarhus University, Aarhus, Denmark; ^5^Unit of Ecology and Evolution, Department of Biology, University of Fribourg, Fribourg, Switzerland; ^6^Instituto de Biología, Universidad de Antioquia, Medellín, Colombia; ^7^Jardín Botánico del Quindío, Armenia, Colombia; ^8^Reserva Natural Guadualito, Montenegro, Colombia

**Keywords:** climatic, environmental niche, geographical overlap, narrow endemic, palms, realized niche, species distribution models, phylogenomics

## Abstract

The topographic gradients of the Tropical Andes may have triggered species divergence by different mechanisms. Topography separates species’ geographical ranges and offers climatic heterogeneity, which could potentially foster local adaptation to specific climatic conditions and result in narrowly distributed endemic species. Such a pattern is found in the Andean centered palm genus *Aiphanes*. To test the extent to which geographic barriers and climatic heterogeneity can explain distribution patterns in *Aiphanes*, we sampled 34 out of 36 currently recognized species in that genus and sequenced them by Sanger sequencing and/or sequence target capture sequencing. We generated Bayesian, likelihood, and species-tree phylogenies, with which we explored climatic trait evolution from current climatic occupation. We also estimated species distribution models to test the relative roles of geographical and climatic divergence in their evolution. We found that *Aiphanes* originated in the Miocene in Andean environments and possibly in mid-elevation habitats. Diversification is related to the occupation of the adjacent high and low elevation habitats tracking high annual precipitation and low precipitation seasonality (moist habitats). Different species in different clades repeatedly occupy all the different temperatures offered by the elevation gradient from 0 to 3,000 m in different geographically isolated areas. A pattern of conserved adaptation to moist environments is consistent among the clades. Our results stress the evolutionary roles of niche truncation of wide thermal tolerance by physical range fragmentation, coupled with water-related niche conservatism, to colonize the topographic gradient.

## Introduction

The Tropical Andes Biodiversity hotspot, also referred to as the uplands of Western Amazonia, spans from Venezuela, Colombia, Ecuador, Peru, Bolivia to Northern Argentina (Myers et al., 2000; Mittermeier et al., 2004, 2011). It ranks first among 36 world hotspots for biodiversity based on species richness and endemism and level of threat, and is estimated to contain nearly one-sixth of all vascular plant species. The causal mechanisms behind the explosion of species richness during the ongoing orogeny of the Tropical Andes have been extensively discussed in the last 2 decades (Hoorn et al., 2010; Antonelli and Sanmartín, 2011; Luebert and Weigend, 2014; Antonelli et al., 2018; Rahbek et al., 2019), and several factors associated with mountain building were identified to promote the extraordinary taxonomic diversification in these areas (Graham et al., 2014; Benham and Witt, 2016; Antonelli et al., 2018). With the creation of a remarkable diversity of novel heterogeneous habitats, organisms could adapt to and/or specialize in new topographic complexity and climatic conditions (i.e., temperature and orographic precipitation). In addition to the strong climatic and ecological gradients that characterize mountain areas, uplift and erosion form new physically constrained habitats potentially leading to a high proportion of mountain endemics by allopatric isolation (Antonelli and Sanmartín, 2011). A particular challenge in determining which factors promote diversification comes from the intrinsic relationship between topographic and climatic gradients during mountain evolution. Furthermore, both space and time dynamics of these factors are important such as climatically driven connection and disconnection of populations (Flantua et al., 2019), speciation extinction and migration over macroevolutionary time (Chazot et al., 2016, 2018), geodiversity (Muellner-Riehl et al., 2019; Rahbek et al., 2019), age, and isolation (Rahbek et al., 2019).

Andean taxa can be largely composed of rare and narrow endemics that require substantial local sampling in hardly accessible sites. Our knowledge of this diversity in the Northern Andes has grown over the last years, especially in Colombia where many areas of the country have now become available for research. This has made it possible to use the Andean palm genus *Aiphanes* Willd. (Arecoideae: Cocoseae: Bactridinae) as a test case. These palms are commonly narrow mountain endemics (Bernal and Borchsenius, 2010; Bernal et al., 2019a), even more so than previously thought, with the description of 14 species in the last 3 decades since the latest monograph (Borchsenius and Bernal, 1996), of which 6 species described in the last 5 years are known from only one locality (Bernal et al., 2017, 2019a,b).

**FIGURE 1:**
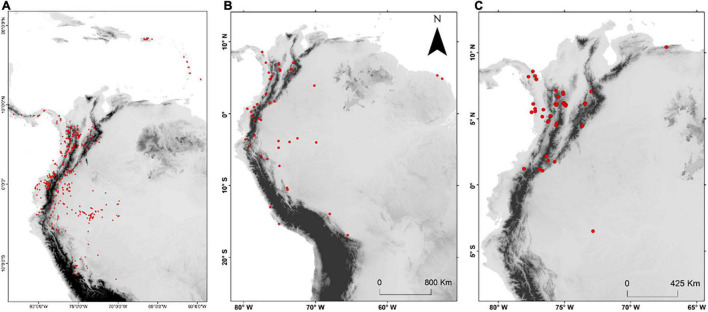
Curated occurrence records for **(A)** all species of *Aiphanes* used in this study, **(B)** all samples used for Sanger sequencing, and **(C)** all samples used for sequence capture.

*Aiphanes* includes 36 species restricted to the understory of lowland or montane forests of the Neotropics, with most species between 6° N and 4° S in the Andes or in the surrounding areas of Western Amazonia and the Choco (Figure 1A). It also includes species that grow up to 21 m tall, like *A. pilaris* R. Bernal and *A. grandis* Borchs. & Balslev, but most are medium-sized, understory palms, and some of them very small or acaulescent. A couple of species grows in dry seasonal habitats (*A. eggersii* Burret *and A. horrida* Burret), one at the mountain tree line (*A. verrucosa* Borchs. & Balslev), and several in the Andean foothills (i.e., *A. macroloba* Burret, *A. acaulis* Galeano & R. Bernal, and *A. buenaventurae* R. Bernal & Borchs), and a single species (*A. argos* R. Bernal, Borchs. & Hoyos-Gómez) is restricted to riverine habitats. Most species occur as inconspicuous or rare elements in cloud forests like *Aiphanes verrucosa* in Ecuador (Svenning et al., 2009).

Definitely, one of the most striking characteristics of *Aiphanes*, when compared to other species-rich Neotropical palm genera, is its paucity. It is uncommon to see extensive or dense populations of *Aiphanes* as it is to see several species of this genus coexisting at the same site. This contrasts with other speciose Neotropical forest palm genera like *Geonoma* Willd. and *Chamaedorea* Willd., which frequently form prominent and abundant populations and local assemblages of several congenerics dominating the understory. Despite their paucity, *Aiphanes* palms have conquered the elevation gradient of the Andes (which we here understand as the foothills and montane environments between 0 and 3,000 m above sea level), whereas most other Neotropical palm genera have not. Do geographical or climatic factors determine this genus’ success in conquering the gradient despite its paucity, low local diversity, and high endemicity?

Moist tropical climates have favored diversification in palms (Svenning et al., 2008), which could be due to higher population sizes (which appear unlikely in *Aiphanes*), biotic interactions, greater ecological success of palms given their specific morphology and anatomy, or water-energy dynamics (Eiserhardt et al., 2011a). Other studies on the geographical ecology of palms suggest that precipitation seasonality can be the most important climatic predictor of species richness (in western Amazonia: Kristiansen et al., 2011), especially if combined with temperature seasonality extremes (in Brazil: Salm et al., 2007). In South America, Neves et al. (2020) found that closely related lineages likely phylogenetically conserve adaptation to a precipitation regime. Here, we want to evaluate the role of geography and climate despite the fact that biotic interactions, morphology, and population sizes can also play important roles.

Phylogenetic studies provide the backbone for hypothesis testing of a causal mechanism underlying divergence. In the case of climatic specialization, they can be used to compare resulting niche breadths of sister species (Bonetti and Wiens, 2014). Different morphological and ecological traits can be compared between same-clade species (i.e., species belonging to a monophyletic clade in the genus) and across the evolution of taxa from different clades to test whether these differ more or less in any groups in particular (Graham et al., 2004; Schnitzler et al., 2012). This, used in combination with species distribution models (SDMs), allows for exploration of the role of geography and climate in species divergence. In principle, phylogenetic niche conservatism states that phylogenetically related species should share the tendency to occupy similar (climatic) niches (Harvey and Pagel, 1991). Similarly, closely related species should occupy closer geographical areas than distantly related ones or should have largely geographically overlapping distributions unless geography is the factor driving their divergence.

Furthermore, dispersal constraints caused by physical barriers can truncate species’ fundamental climatic niche (Feeley and Silman, 2010), enforcing differentiation of the realized niche between closely related species. In Australia, thermal truncation was prevalent especially in forest species. In that study, the truncation of water-related climatic space was not assessed (Bush et al., 2018). In the case of mountains, where physical barriers are known to be important factors causing species divergence (Antonelli et al., 2018), we expect the realized climatic niche of species to become a specialized subset of their potential climatic niche resulting in small realized niche widths. We also expect that the capacity to specialize (from a wider fundamental climatic tolerance) plays a role in species’ colonization of the elevation gradient. Here, we seek to understand which aspects of the climatic niche are conserved and which are free to change and specialize in the midst of physical range fragmentation.

In this article, we assess two main drivers for *Aiphanes* divergence patterns that correlate to mountain building across the Andean Cordilleras. The first one alludes to climatic specialization, which was assessed by current climatic space occupancy based on herbarium records, and its evolution was reconstructed on phylogenies. The second one alludes to physical isolation of populations over time (divergence mediated by geographical isolation) assessed by SDMs and their overlap between species. In contrast to the expectations of phylogenetic niche conservatism, we predict same-clade species to occupy a significantly different (non-overlapping) climatic space. We also predict non-overlapping geographical distributions between same-clade species. Thus, we expect species to depart from climatic niche conservatism because topographic gradients offer steep and wide climatic gradients in which species can specialize favored by isolation in physically confined areas. These areas truncate species climatic niche. We conducted an extensive field collection for genetic sampling covering all but two species of *Aiphanes* (*A. bio* R. Bernal, Borchs., Hoyos-Gómez, H.F. Manrique & Sanín and *A. cogollo* R. Bernal, Borchs., Hoyos-Gómez, H.F. Manrique & Sanín) to build genus-wide phylogenies combining classical Sanger sequencing with a target enrichment bait set of more than 4,000 targeted genes. Phylogenetic relationships are used to discuss how the climatic and geographical occupations contrast between same-clade species, and how climate-related traits evolve between clades and species.

## Materials and Methods

### Taxon Sampling and Genomic Data Obtainment

#### DNA Extraction

For all samples in both the target capture and Sanger sequencing datasets, we used a DNeasy^®^ plant mini kit (Qiagen, Venlo, Netherlands) following the supplier’s instructions for DNA isolation. DNA quantities were measured with a NanoDrop™ spectrophotometer (Thermo Fisher Scientific, Waltham, MA, United States). All extracted samples and their use for different phylogenies are listed in Supplementary Tables 1, 2.

#### Sampling for the Sanger Sequencing Phylogeny

We used 64 samples (Figure 1B) representing 31 of the 36 species currently accepted in the genus. Whenever possible, we included several individuals per species; for widely distributed species with described subspecies or forms (such as in *A. hirsuta* where there are four described subspecies: Borchsenius and Bernal, 1996), we aimed at sampling from geographically distant localities. Furthermore, we sampled 11 individuals representing 11 species of the other four genera of the subtribe Bactridinae (*Acrocomia* Mart., *Astrocaryum* G. Mey., *Bactris* Jacq. ex Scop., and *Desmoncus* Mart.).

#### Amplification and Sequence Preparation

For Sanger sequencing, we amplified three nuclear genome regions that are commonly used in palm systematics: ITS, prk, and rpb2. We used classical PCR parameters, as described by Eiserhardt et al. (2011b). Chromatograms were visually checked for erroneous or ambiguous nucleotide calling. Supplementary Table 1 lists all the samples included and genes that were amplified for each sample, as well as the accession codes for each marker on GenBank. DNA Sequences were aligned for each gene independently using Muscle (Edgar, 2004) as implemented in the EMBL-EBI search and sequence analysis tools (Madeira et al., 2019) followed by a visual check.

#### Sampling for the Sequence Capture Phylogeny

We gathered 98 samples (Figure 1C) representing 23 species of predominantly Colombian species complexes of *Aiphanes*, totaling 64% of the genus and focusing on groups that we considered problematic or poorly studied including *Aiphanes parvifolia* Burret and recently described and morphologically similar species (Bernal et al., 2019a), *A. lindeniana* H. Wendl., *A. hirsuta* Burret, and *A. simplex* Burret complexes. In these cases and in few others, we included 2–31 individuals per species, covering different forms or subspecies that have been described as well as the widest possible geographic distribution. The most densely sampled species was *A. hirsuta*, covering all four recognized subspecies. The sampling concentrated on the Andes of Colombia, on the Central, Western Cordilleras, and the Pacific lowlands (including Gulf of Tribugá where it had not been previously registered), where taxonomic novelties and micro-endemism seemed more relevant because of recent findings (Bernal et al., 2019a,b). We also sampled 24 individuals in 23 species from other palm genera as outgroups (refer to Supplementary Table 2 for a full list with coordinates, herbarium samples, and accession codes). For a brief description of the sampling schemes for both sequencing approaches, refer to Table 1 and Supplementary Material in this article.

**TABLE 1:** Number of *Aiphanes* individuals sampled for each sequencing approach.

	Species	Sanger sequence	Sequence capture
1	*Aiphanes acaulis* Galeano & R. Bernal	1	1
2	*Aiphanes argos* R. Bernal, Borchs., Hoyos-Gómez	2	2
3	*Aiphanes bicornis* Cerón & R. Bernal	1	0
4	*Aiphanes bio* R. Bernal, Borchs., Hoyos-Gómez, H.F. Manrique & Sanín	0	0
5	*Aiphanes buenaventurae* R. Bernal & Borchs.	1	2
6	*Aiphanes chiribogensis* Borchs. & Balslev	1	0
7	*Aiphanes cogollo* R. Bernal, Borchs., Hoyos-Gómez, H.F. Manrique & Sanín	0	0
8	*Aiphanes concinna* H.E. Moore	0	7
9	*Aiphanes decipiens* R. Bernal, Borchs., Hoyos-Gómez, H.F. Manrique & Sanín	1	2
10	*Aiphanes deltoidea* Burret	1	1
11	*Aiphanes duquei* Burret	1	0
12	*Aiphanes eggersii* Burret	2	0
13	*Aiphanes erinacea* (H. Karst.) H. Wendl.	4	1
14	*Aiphanes gelatinosa* H.E. Moore	0	1
15	*Aiphanes gloria* R. Bernal, Borchs., Hoyos-Gómez, H.F. Manrique & Sanín	2	3
16	*Aiphanes graminifolia* Galeano & R. Bernal	1	0
17	*Aiphanes grandis* Borchs. & Balslev	1	0
18	*Aiphanes hirsuta* Burret	6	31
19	*Aiphanes horrida* (Jacq.) Burret	6	3
20	*Aiphanes killipii* (Burret) Burret	1	2
21	*Aiphanes leiostachys* Burret	1	2
22	*Aiphanes lindeniana* (H. Wendl.) H. Wendl.	3	9
23	*Aiphanes linearis* Burret	2	12
24	*Aiphanes macroloba* Burret	1	3
25	*Aiphanes minima* (Gaertn.) Burret	1	0
26	*Aiphanes multiplex* R. Bernal & Borchs.	1	0
27	*Aiphanes parvifolia* Burret	1	2
28	*Aiphanes pilaris* R. Bernal	1	1
29	*Aiphanes simplex* Burret	2	5
30	*Aiphanes spicata* Borchs. & R. Bernal	2	0
31	*Aiphanes suaita* R. Bernal, Sanín & Castaño	1	3
32	*Aiphanes tatama* R. Bernal, Borchs., Hoyos-Gómez, H.F. Manrique & Sanín	1	1
33	*Aiphanes tricuspidata* Borchs., R. Bernal & M. Ruiz	1	2
34	*Aiphanes ulei* (Dammer) Burret	7	2
35	*Aiphanes verrucosa* Borchs. & Balslev	1	0
36	*Aiphanes weberbaueri* Burret	6	0
	Assorted Arecaceae	11	24
	TOTAL	75	122

#### Dual-Indexed Library Preparation and Target Capture Sequencing

For nuclear target sequencing, a total of 500 ng was fragmented to 400-bp fragments with a Bioruptor^®^ ultrasonicator (Diagenode, Liège, Belgium). Library preparations were performed following de La Harpe et al. (2019) and using a KAPA LTP library preparation kit (Roche, Basel, Switzerland) for sample cleaning, end-repair, and A-tailing steps, and the protocol of Meyer and Kircher (2010) for adaptor ligation and adaptor fill-in reactions steps. Four μl of the ligated fragment solution were amplified for eight cycles using KAPA HiFi DNA Polymerase (Roche, Basel, Switzerland) and the set of 60 dual index primers described in Loiseau et al. (2019). Libraries were quantified with a Qubit^®^ Fluorometer v2.2 before pooling in equimolar ratio. Target capture was performed using the custom kit PopcornPalm developed by de La Harpe et al. (2019), and targeting 4,051 palm genes. Target capture was conducted on pools of 50 or 51 samples, following myBait^®^ Custom Target Capture Kits protocol v3.0 (Arbor Biosciences, Ann Arbor, MI, United States), with 18 h of incubation time at 65°C and 12 cycles of post-capture PCR reactions. The pooled target capture reactions were quantified with Qubit^®^ Fluorometer v 2.2, before sequencing with an Illumina HiSeq 3000 sequencer (Illumina, San Diego, CA, United States) in paired-end 2 × 150-bp mode.

#### Read Trimming, Mapping, and SNP Calling

The program ConDeTri v2.2 (Smeds and Künstner, 2011) was used to trim the raw reads, with 20 as high-quality threshold parameter. The program bowtie2 v2.2.5 (Langmead and Salzberg, 2012) with the very sensitive local option was used for read mapping. We used the *Geonoma undata* reference genome (de La Harpe et al., 2019) for mapping; it was the closest reference genome available. The proportion of in−target reads (specificity) and the proportion of baits covered (efficiency) were calculated for each sample using bedtools v2.24.0 (Quinlan and Hall, 2010) following de La Harpe et al. (2019). The target capture method was highly successful for all the *Aiphanes* species and samples included in our analyses, with average specificity of 79.3% (range: 74.9–81.1%) and average efficiency of 91.3% (range: 76.4–95.4%). We then selected reads mapping at a unique location on the genome and masked PCR duplicates with Picard tools v1.119^1^. The program GATK v3.8 (McKenna et al., 2010) was used to realign the reads around indels, base-recalibration, and SNPs calling for target regions, using UnifiedGenotyper with the EMIT_ALL_SITES option in order to obtain both variable and invariable sites. Sites were filtered using VCFtools v0.1.13 (Danecek et al., 2011), with a minimum quality of 20, a minimum depth of 8× per sample, and a maximum of 50% of missing data, and by removing indels. After filtering, the targeted capture method provided a total of 2.557.512 high-quality sequenced bases with an average coverage of 29.2× per sample and distributed in 2,867 genes. The bait kit developed by de La Harpe et al. (2019) for micro- and macro-evolutionary analyses of palms is large (4,051 genes) and contains fast- to slow-evolving DNA regions. We included the whole set of markers for this study, because we sampled both at the species and “morphotype” levels. We also aimed at having a robust phylogeny that relies on informative data and could serve as a backbone or a phylogenetic framework for future studies involving more specific questions on *Aiphanes*.

### Phylogenetic Inference

#### Phylogenetic Analysis of the Sanger Sequences

Two Bayesian phylogenies were generated in BEAST v 1.10 (Suchard et al., 2018). The three different DNA regions were concatenated and partitioned, and the site model was unlinked. The evolutionary site model was selected using the Akaike Information Criterion (GTR + *invariant sites* for ITS, TN92 for prk, and GTR+GAMMA for rpb2) in jModelTest (Guindon and Gascuel, 2003; Darriba et al., 2012). We chose a lognormal uncorrelated relaxed clock model to account for rate heterogeneity and the birth death tree branching prior. The analyses were run for 2 chains of 100.000 generations each. ESS values (>200) and chain convergence were assessed in TRACER v 1.7.1 (Rambaut et al., 2018). For each different analysis, trees were combined and summarized in the LogCombiner and TreeAnnotator (maximum clade credibility tree with a posterior probability limit of 0.5 and burn-in of 10%) applications of the BEAST 1.10 package (Suchard et al., 2018). The resulting maximum clade credibility tree will be hereafter referred to as the Sanger sequence phylogeny (SSP).

#### Phylogenetic Analyses of the Target Capture Sequences

Phylogenetic trees were estimated using the maximum-likelihood and coalescent-based species tree methods. The choice to use both methods stems from the expectation that different gene trees could lead to different phylogenetic relationships (Liu et al., 2015a), something that could not be accounted for using concatenated DNA regions in a Maximum Likelihood reconstruction alone. This was of particular importance in this data set where we concentrated our sampling in species complexes that included several described forms or newly described taxa. It was our priority to be able to conduct species clade assignment in the downstream analyses that relies on accounting for possible incongruence between gene tree topologies. The concatenated alignment of the 2,557.512 high-quality bases, including both variable and invariable sites and distributed in 2867 genes, was analyzed with RAxML v8.228 (Stamatakis, 2014) using the GTR+GAMMA model of substitution and 100 rapid bootstrap replicates. The concatenation of a large number of genes often results in phylogenetic trees with high node support values, but the assumption that all genes share the same topology and branch lengths is often violated and can lead to high support for the wrong topology (Kubatko and Degnan, 2007). Coalescent-based methods are better suited for datasets with multiple loci, as they consider gene tree incongruence due, for example, to incomplete lineage sorting (Liu et al., 2009). We therefore used ASTRAL v5.6.1 with default parameters; ASTRAL is a faster, two-steps coalescent-based method that estimates the species tree, given a set of gene trees (Mirarab et al., 2014; Mirarab and Warnow, 2015). We used the *-a* option from Rabiee et al. (2019), 515 genes and 40 bootstrap replicates per gene, with each gene contributing equally to localPP support. For gene selection, a first list of genes was selected based on missing data, retaining 1,993 genes with more than 400-bp sequence length covered after filtering for high-quality bases, including both variable and invariable sites, and with sequence information for all the samples (i.e., no sample consisted entirely of missing data). Gene trees were first estimated with RAxML v8.228 (Stamatakis, 2014) using the GTR+GAMMA model of substitution and 100 bootstrap replicates. Weakly informative gene trees with average bootstrap values lower than 40 were not kept for further ASTRAL analysis in order to avoid a potential decrease in the accuracy of species tree estimation (Liu et al., 2015b; Molloy and Warnow, 2018). A total of 515 highly informative selected genes exhibited an average length of 1,894 bp (range: 475–11,383 bp), more than two times higher than the average length of 920 bp for weakly informative genes (range: 400–5,112 bp). This number of highly informative genes detected with our analyses involving mainly *Aiphanes* samples is concordant with the number of 795 highly informative genes detected by Loiseau et al. (2019) using the same capture kit and 20 palm samples representing a wide range of evolutionary time scales, from intra-specific variability of up to 88 Ma of divergence. The RAxML and the ASTRAL trees will be hereafter referred to as the sequence capture phylogenies (S).

### Phylogenetic Dating

The SSP was dated by the following secondary calibrations obtained from Eiserhardt et al. (2011b): core north Andean clade at 11 My and crown of genus at 28 My, both using normal distribution with a standard deviation of 1, a log-normal relaxed clock, and a birth death branching prior.

Dating of the SCPs was performed by penalized likelihood using the function *chronos* in “ape” R package v. 5.4–1 (Paradis et al., 2004; Paradis, 2013) (lambda smoothing parameter = 2, model = correlated) and node calibration for the crown *Aiphanes* (age.min = 27 and age.max = 29), and a second calibration age resulting from population parameter estimation in the coalescent. This divergence age was estimated for *lindeniana* + *linearis* clades in SNAPP (Bryant et al., 2012) implemented in Beast 2.6 (Bouckaert et al., 2019) on CIPRES Science Gateway version 3.3 (Stamatakis et al., 2008; Miller et al., 2010). For this age estimation, we included all sampled individuals of *A. hirsuta, A. lindeniana, A. linearis*, and *A. concinna* in the SCP. We ran 5 chains with a randomly resampled matrix of 800 nuclear SNPs for 10 million generations and then checked them in Tracer v (Rambaut et al., 2018) for chain convergence, sampling efficiency of the priors, likelihood and posterior as well as mutation rates, and theta parameters (these two were left to estimate). Time was obtained from node height of the maximum clade credibility tree after 10% burn-in by conversion using the mutation rate for corn (*m* = 2.61 × 10^–9^) (Gaut et al., 1996).

### Species Environmental Distribution Models

Species ranges were estimated by extracting species occurrence data from GBIF using the “rgbif” package (version 2.2.0) (Chamberlain et al., 2022) and other data sources (Herbaria: HUA, JAUM, and UTMC). To clean up common errors in the occurrence data, we used QGIS (version 10.2). The coordinates were compared with maps made by experts (Henderson et al., 1995; Borchsenius and Bernal, 1996; Dransfield et al., 2008; Bernal and Borchsenius, 2010; Galeano and Bernal, 2010), as we explain below. Expected distributions were taken from drawn polygons from the existing literature (cited above) and were overlaid to each species coordinates to check for outliers. When we found a dubious point, it was checked by RB (coauthor). If the point could not be verified (or the taxonomy was dubious, meaning the point could have been erroneously assigned to another species), it was discarded.

Georeferencing precision was assessed following Escobar et al. (2016) and verifying with official Base Cartography that the sites described coincided with the coordinates of the biological records. Multicollinearity was evaluated on bioclimatic layers from where the accessible area (m area for species) was cut using an “Extract by mask” algorithm (ArcMap algorithm). Accessible areas (m) were defined by selecting Olson ecoregions that intersected with biological records (Barve et al., 2011). We also performed attribute filtering to eliminate incomplete information, duplicate records, and data from years before 1979. Finally, we stored this information in a geodatabase using the ArcCatalog software (version 10.5).

We used the 19 bioclimatic variables developed by Karger et al. (2017) and available online. We chose CHELSA layers because they incorporate corrections for the effect of wind, the boundary layer, and exposures in mountain valleys (Karger et al., 2017). The layers Bio1-Bio19 incorporate data from the time period of January 1979 to December 2013 and are available at 1 km (30 arcs) resolution. We extracted all climatic layer values for each curated occurrence point using the accessible areas (M) as a mask. This M area was based on the delineation of ecoregions (Olson et al., 2001) and the species range proposed by different authors (Henderson et al., 1995; Galeano and Bernal, 2010; Galeano et al., 2015). We eliminated the correlated predictors using the variance inflation factor (VIF) with the R package *usdm* v 1.18 (Naimi et al., 2014). Seven bioclimatic variables did not exhibit collinearity (VIF < 10) (Supplementary Table 3). All used coordinates fell within 20° N and 18° S and 60° W and 81° W.

Species distribution models were generated with Maxent maximum entropy algorithm v 3.4.1 (Phillips et al., 2017). This algorithm has been widely used, generating adequate results in exploration of niches and species distribution (Altamiranda-Saavedra et al., 2017; Calixto-Pérez et al., 2018). Maxent has the advantage of providing an evaluation of omission/commission, response curves, and analysis of variable contributions, which is highly useful for understanding the outputs of the model. The background points were generated on the M area to avoid inflation of the AUC. We generated 10 repetitions per species applying the bootstrapping technique and randomly partitioned the species data in each replicate (85% training and 15% validation). All the models were run using default settings (10,000 background points, 500 maximum iterations with a 10^–5^ convergence threshold, regularization multiplier of 1, and duplicate occurrence removal). The predictive capacity of each model was assessed using the area under the curve (AUC) value that is generated using the ROC (receiver operating characteristic) technique performed by Maxent. The best model was kept ensuring a test AUC ≥ 0.9 for each species. Finally, the best model of each species was projected to geography and reclassified using a threshold that represents the lowest rate of omission in the data training and testing (<15%). The result was a binary polygon (1/0: presence/absence) (Elith et al., 2011; Phillips et al., 2017). For species with few records and known as endemic to only a few pixels, manual models were produced by reclassifying the digital elevation model Alos Palsar (pixel size: 12.5 m × 12.5 m) to the elevation reported in the coordinates of the few herbarium samples available, corresponding to the narrow distribution of the species. Then, the reclassified raster was cut with watersheds that included all the coordinates.

### Measuring Niche Overlap

We tested for niche overlap following the environmental-PCA method proposed by Broennimann et al. (2012) and implemented in the R package *ecospat* v. 3.1 (Di Cola et al., 2017). A PCA on the selected predictors by the VIF was computed, and the resulting environmental space for the study area was gridded at 1 km^2^ cell resolution. Then, a smooth kernel density function was applied to the occurrence records plotted on the gridded environmental space. The observed niche overlap score for each species pair was estimated with Schoener’s *D* metric, which ranges from 0 (no overlap) to 1 (complete overlap) (Warren et al., 2008). Then, statistical tests for niche similarity and niche equivalence hypotheses (Warren et al., 2008) were performed. The first test evaluated whether the ecological niche occupied by two lineages were identical and the second assessed whether the ecological niches of two entities were more or less similar than expected by chance. We repeated each test 100 times, returning a null distribution of overlap values to which the observed niche overlap (D) was compared. If the observed *D* value fell outside of the 95th percentile of expected *D* values, the null hypothesis of random equivalency/similarity was rejected. Finally, we evaluated if the mean *D* values per clade was negatively correlated to the age of each clade [Pearson method implemented in *cor.test* function of the *stats* package in R Core Team (2020)].

### Geographic Distribution and Climatic Evolution

The climatic data for statistical analysis were extracted from the previously curated occurrence points overlaid on the CHELSA layers. The temperature-related (Bio1-Bio11) and precipitation-related variables (Bio12-Bio19) were explored by principal component analyses on temperature and precipitation biplots. Four variables were chosen based on their orthogonality in the PCA, on Sanín et al. (2016), and on our knowledge of factors that could be related to topography and that could determine plant growth; these were: mean annual temperature (Bio1), temperature during the coldest month (Bio6), annual precipitation (Bio12), and precipitation seasonality (Bio15). Although we could have reconstructed the seven variables from VIF, this analysis was exploratory and independent from the SDMs; our aim was to see how they evolved on the phylogenies. These variables were reconstructed as continuous traits on the SCP and SSP phylogenies using the *Rphylopars* R package v 0.3.2 (Bruggeman et al., 2009) under the Brownian motion default function of continuous trait evolution by Ho and Ané (2014) and using the mean of each species of all values extracted from all occurrences available for each species. We also used these values to conduct a PGLS (phylogenetic generalized least squares) test for phylogenetic signal of two variable sets: the temperature-related and precipitation-related variables using the *pgls* and *pgls.profile* functions of the package *caper* v 1.0.1 (Orme et al., 2012).

## Results

### Phylogenetic Reconstructions

**FIGURE 2:**
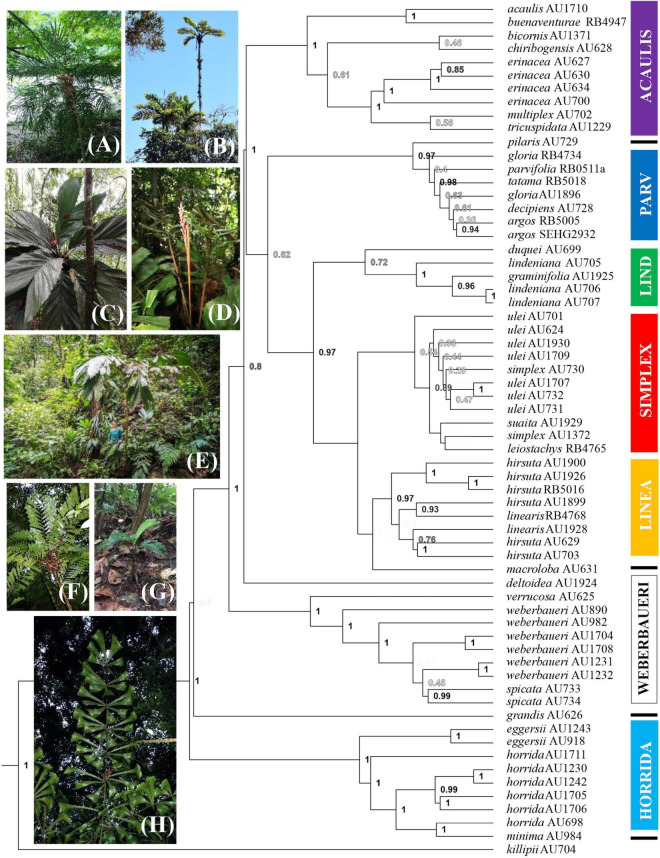
Bayesian phylogeny of the genus *Aiphanes* by Sanger sequencing. Colors of clades marked by boxes follow online Supplementary Figures 1–4, 6 and Figure 5; the lines indicate species not assigned to clades; support values as posterior probabilities are shown in shades of gray with higher support values in black; epithets are followed by voucher code. Photos show **(A)** *Aiphanes argos* (*parvifolia* clade): habit; **(B)** *A. concinna* (*lindeniana* clade): habit; **(C)** *A. cogollo* (*parvifolia* clade): crown; **(D)** *A. bio* (*parvifolia* clade): inflorescence; **(E)** *A. hirsuta* (*linearis* clade): habit; **(F)** *A. leiostachys* (*simplex* clade): crown; **(G)** *A. macroloba*: habit*;* **(H)** *A. killipii*: funneled pinnae. Photographs: **(C)** by Alvaro Cogollo, **(D)** by Camilo Flórez, **(E)** by Felipe Mesa.

The SSP (Figure 2) shows seven clades that agree with the SCPs, although these clades are not all well-supported [posterior probabilities (PP) of 1 for clades *acaulis*, *weberbaueri*, and *horrida*; PP of 0.97 for clades *parvifolia* and *linearis*; PP of 0.72 for clade *lindeniana*; PP of 0.54 for clade *simplex*]. Support for this seven-clade backbone is strengthened in the SCPs that we discuss below (for support values in all trees, refer to Table 2). Thus, the SSP provides a backbone showing several important relationships: (a) *Aiphanes killipii* as sister to the rest of the genus, (b) the *horrida* clade is supported, with three allopatrically distributed species, (c) *A. grandis* as sister to the mainly Andean species placed by Burret (1932) in subgenus *Brachyanthera*, and (d) the main pattern inside the *Brachyanthera* clade. This main pattern shows a well-supported *weberbaueri* clade, a well-supported *acaulis* clade, and the remaining predominantly Colombian species organized into four clades (*lindeniana*, *linearis*, *parvifolia*, and *simplex* clades).

**TABLE 2:** Support values (as posterior probabilities) of *Aiphanes* clades for the Bayesian and ASTRAL analyses, and in Bootstrap values for RAxML.

	Clade Support		
	
Clades	Sanger sequence Bayesian	Sequence Capture ASTRAL	Sequence Capture RAxML
*acaulis*	1	1	100
*parvifolia* + *pilaris*	0.97	NA	NA
*parvifolia*	0.4	1	100
*lindeniana*	0.72	1	100
*deltoidea*	NA	NA	NA
*horrida*	1	1	100
*grandis*	NA	Not studied	Not studied
*linearis*	0.97	1	100
*killipii*	NA	1	100
*simplex*	0.54	1	100
*macroloba*	NA	1*	100*
*pilaris*	NA	NA	NA
*weberbaueri*	1	Not studied	Not studied

*NA, not applicable, meaning not recovered within a clade but as a grade.*

*Asterisk stands for support for a single species sampled with more than one accession.*

**FIGURE 3:**
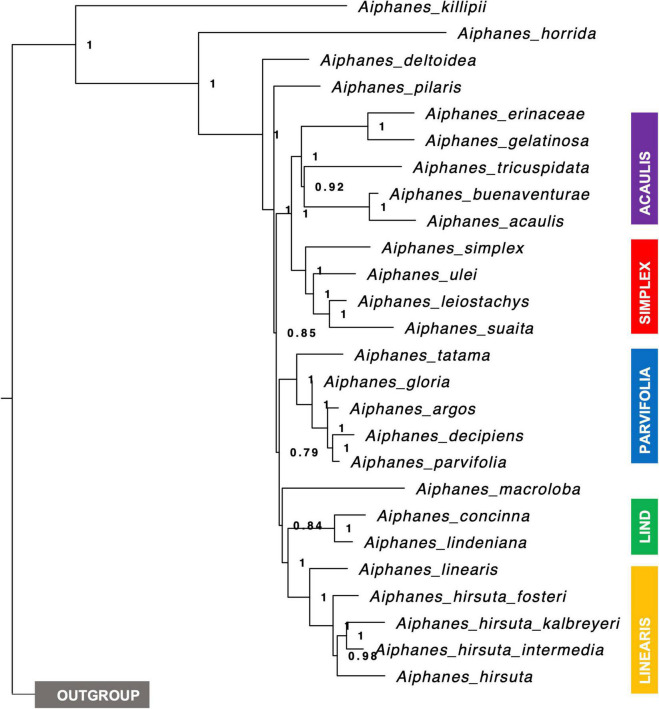
Species tree of *Aiphanes* where tips are represented by a species (or subspecies) and not by each sampled individual based on 515 genes. Node branches show local PP support, and clades are color-coded to match Figure 2.

The SCPs provide additional resolution to groups that the Sanger tree did not resolve. The targeted capture method provided a total of 2,557,512 high-quality sequenced bases, with an average coverage of 29.2× per sample. The Maximum Likelihood concatenated RAxML (SCP) and ASTRAL (SCP) topologies reveal similar relationships (Figure 3 and Supplementary Figures 1, 2), with a few exceptions of infraspecific accessions. Support values in these two topologies are significantly higher than in the SSP in the mainly Colombian species (Table 2). Both samplings are not equivalent, as the SSP includes more species and the SCP includes more and complementary accessions for the poorly supported and less studied clades *parvifolia*, *lindeniana*, *simplex*, and *linearis*; only *A. bio* and *A. cogollo* were left unsampled altogether.

There were several important topologic dissimilarities between the trees obtained from different sequencing strategies, with the most relevant being not the species included in clades but the relationship between clades (i.e., the backbone relationships between the clades, not clade definition). Thus, we chose to discuss the clade relationships obtained by the SCPs, as the resulting supports were higher and sequencing more extensive in the genome, and used the SSP for clade definition (i.e., species placement into clades) because of the inclusion of *A. verrucosa* and *A. grandis*, as well as *A. graminifolia* Galeano & R. Bernal, *A. weberbaueri* Burret, and *A. spicata* Borchs. & R. Bernal in the sampling. An important inconsistency was related to two of the species forming monospecific clades, *A. pilaris* and *A. macroloba. A. pilaris* was sampled in both approaches and was recovered as sister to the *parvifolia* complex in the SSP but not in the SCP, where it was recovered as sister to everything but *A. killipii* Burret, the *horrida* and *weberbaueri* clades. In the SCP, *A. macroloba* was placed as sister to both the *lindeniana* and the *linearis* clades, whereas in the SSP it was placed as sister only to the *linearis* clade. Despite these differences, the strategies were complementary by providing species assignment into the clades (SSP) plus relationships between the clades (SCP).

Using the two different calibration schemes for the SSP and SCP resulted in similar divergence age estimates, concentrating in the Miocene-Pliocene. The SNAPP analyses resulted in an estimated divergence time between the *lindeniana* and *linearis* clades of 2-1 million years before the present, and tree inconsistencies regarding the relationship among the four included species (Supplementary Table 4).

### Geographical Distribution and Overlap Between Same-Clade Species

The first branching *A. killipii* has a narrow distribution in the Eastern Cordillera of Colombia. Further splits in the phylogeny produce the widely distributed *horrida* clade, spanning the Tropical Andes and the Caribbean, a *weberbaueri* clade from mostly Peru and Ecuador, the Colombia and Ecuador Pacific *acaulis* clade, and then the four most nested and predominantly Colombian Andean *linearis*, *lindeniana*, *parvifolia*, and *simplex* clades.

The species of *Aiphanes* present a very small overlap of distribution ranges, with most species in clades showing a less than 10% overlap. Average range overlap was highest in the *deltoidea* and *linearis* clades (38 and 31%, respectively), followed by the *acaulis* and *lindeniana* clades (18 and 16%, respectively), and with the other clades showing a less than 10% average overlap. Most of the overlapped geographical ranges include a species that is microendemic (known from one or a few close localities) embedded in a range of a widely distributed species (i.e., 99% of *A. spicata’s* range is within *A. weberbaueri’s* but only 1% vice versa). Overlap of 31–92% did occur between widely distributed Andean sister species pairs: *A. concinna*/*A. lindeniana*, and *A. hirsuta*/*A. linearis*. The geographical distributions are available in Supplementary Figure 3 (distribution point maps) and Supplementary Figure 4 (SDMs used to estimate geographical overlap); the table with all the paired overlap estimates is available in Supplementary Table 5.

### Evolution of the Climatic Niche in *Aiphanes*

#### Temperature

**FIGURE 4:**
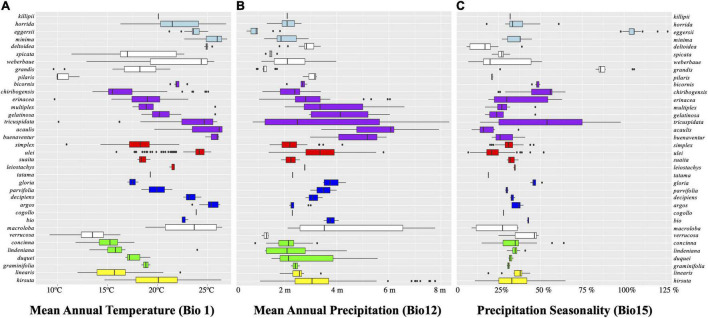
Boxplots for three of the variables evaluated grouped by clades indicated by the same colors from Figure 2. **(A)** Bio1, mean annual temperature in Celsius degrees; **(B)** Bio12, mean annual precipitation in 1 year in kg m^–2^; **(C)** Bio15, precipitation seasonality in meters of rain in kg m^–2^ (i.e., the standard deviation of monthly precipitation estimates is expressed as a percentage of their mean).

Figure 4 and Supplementary Figure 5 show the mean annual temperature (MAT, or Bio1) reconstruction throughout the Bayesian tree. The genus is and has been adapted to temperatures of ca. 18°C. This MAT is not typical of the lowlands but of mid-elevation forests, between 1,000 and 2,000 m under current climate. Several times independently in the phylogeny, *Aiphanes* species adapt to either colder (close to 11°C of MAT in *A. concinna* H.E. Moore, *A. pilaris*, and *A. ulei* Burret, shown in blue) or warmer MAT (near 26°C, as *A. acaulis, A. horrida*, and *A. deltoidea* Burret, shown in red). The ancestral states of the earliest nodes in *Aiphanes* indicate intermediate values for MAT. Isothermality (Bio3) shows a pattern similar to that of MAT. The two higher clades reconstructed under the SCP (i.e., *lindeniana* + *linearis* + *macroloba* and *acaulis* + *simplex* + *parvifolia*) have lowland, midland, and highland species, covering the full topographic gradient for the whole genus and thus covering the gradient for both MAT and other indicative variables such as isothermality and temperature of the coldest month.

#### Precipitation

Figure 4 and Supplementary Figure 5 show that mean annual precipitation (MAP, or Bio12) is in the 2,000–4,000 mm/yr range, with high elevation species adapting to less MAP above 1,700 mm/yr and few coastal or lowland species adapting to higher MAP under 6,000 mm/yr. The ancestral state for all *Aiphanes* for MAP is close to 2,000 mm/yr. Precipitation seasonality (Bio15: the differences in rainfall occurring throughout the year), varies only in a few species in the *acaulis, horrida*, and *parvifolia* clades, which indicates that *Aiphanes* mostly occurred in less seasonal ecosystems in terms of rainfall. Annual precipitation and precipitation seasonality varied inversely, with progressive evolution of the genus toward higher annual precipitation and lower precipitation seasonality. Only two species from the Colombian Choco occur in areas with high annual precipitation of around 6,000 mm/yr: *A. buenaventurae* R. Bernal and Borchs. and *A. acaulis*. Only one species occurs in areas with high precipitation seasonality: *A. eggersii* Burret.

Figure 4 shows the breadth of occupancy by species (rows) and clades (colors from SSP and SCPs) for three climatic variables that are representative of annual variation (mean annual temperature and precipitation, MAT and MAP, and precipitation seasonality). MAT shows segregation between species, whereas MAP and precipitation seasonality tend to be closer between species in clades. Thermal occupation is divided among species of each clade, as can be seen in the “laddered” distribution of the boxes within each color-coded clade.

#### Climatic Niche Overlap Between Same-Clade Species

**FIGURE 5:**
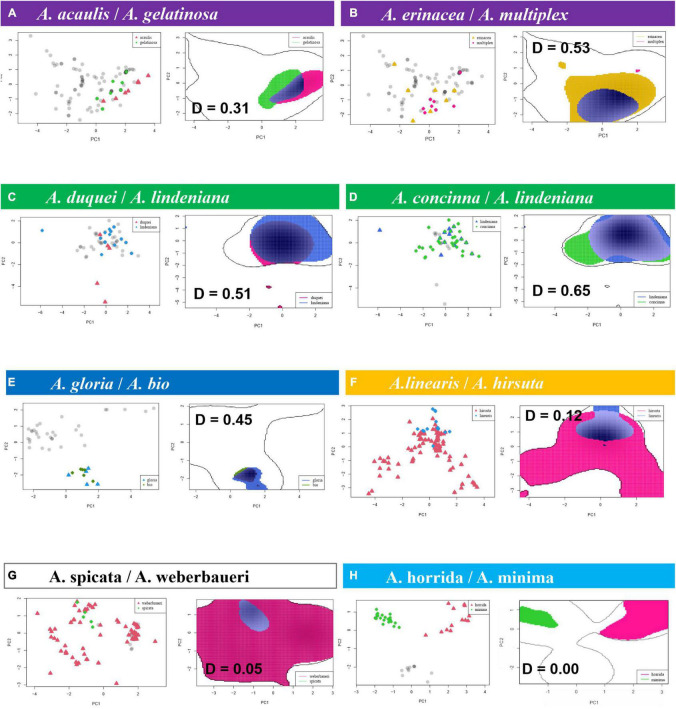
Niche overlap, projected on the two first components of the variance inflation factor variable principle component analyses, can occur in relation to a wide variety of geographical species ranges: **(A,E)** between two microendemics; **(C,G)** between a microendemic and a widely distributed species, **(B,D,F)** between two widely distributed species occurring in the **(B)** lowlands, **(D)** highlands, or **(F)** throughout the elevation gradient; **(H)** two widely distributed same-clade species with entirely non-overlapping niches. Schoener’s *D* in bold, including the highest level of observed overlap between same-clade species (*D* = 0.65 in the *lindeniana* clade) and the lowest possible value (*D* = 0) observed for many same species pairs and all species pairs in the *horrida* clade. The same-clade species here are understood as belonging to the same outline clade (in colors).

The species pair niche comparisons yield significant differences for most same-clade species pairs (Figure 5 and Table 3; refer to Supplementary Figure 6 for niche volume overlap projections on the two first principal components encompassing 75% of variance and Supplementary Table 5 for Schoener’s *D* index for all species pairs in the clades). Schoener’s *D* indices for all species pairs were relatively low, with a minimum of 0, a maximum of 0.65, mean = 0.08, and median = 0.01). The geographically overlapped same-clade species pair (*A. concinna*/*A. lindeniana*) also have significantly overlapped niches from the VIF variable set (Schoener’s *D* = 0.65). Climatic niche overlap of species pairs, as measured by Schoener’s *D*, is highest in the *acaulis, lindeniana*, and *parvifolia* clades. Niche overlap is, in many cases, related to geographical overlap and is most common between species pairs where at least one species has a very narrow distribution (is only known from one or few localities). Pearson correlation between the average Schoener’s *D* value per clade and the age of the clade resulted in a weak negative correlation (-0.349) that is not statistically significant (*p*-value = 0.4425).

**TABLE 3:** Niche and geographical range overlap of species pairs in clades; for complete list of species pairs, refer to Supplementary Table 5; here, we only show species pairs where Schoener’s *D* index or *p*-values for equivalency and similarity indicate that climatic niches are not different; we also show their corresponding geographical range overlaps (ROs) in km^2^.

Clade	Species pairs	Schoener’s *D* Index	*p*-value *equivalency*	*p*-value *similarity*	Area sp. 1 (km^2^)	Area sp. 2 (km^2^)	RO sp. 1 vs. 2	RO sp. 2 vs. 1
ACAULIS	*acaulis vs. gelatinosa*	0.3084	0.91089	0.17822	12505.7	15307.4	0.07	0.09
ACAULIS	*buenaventurae vs. gelatinosa*	0.2305	0.9109	0.0396	913.7	15307.4	0.00	0.00
ACAULIS	*multiplex vs. gelatinosa*	0.2104	0.9802	0.2376	27022.8	15307.4	0.43	0.76
ACAULIS	*erinacea vs. gelatinosa*	0.2605	0.8317	0.1485	64697.2	15307.4	0.00	0.84
ACAULIS	*erinacea vs. multiplex*	0.5246	0.5149	0.0099	64697.2	27022.8	0.34	0.81
LINDENIANA	*duquei vs. lindeniana*	0.5093	0.4554	0.3366	608.6	148344.8	0.36	0.00
LINDENIANA	*duquei vs. concinna*	0.4422	0.2475	0.2178	608.6	79175.9	0.24	0.00
LINDENIANA	*lindeniana vs. concinna*	0.6455	0.1287	0.0693	148344.8	79175.9	0.49	0.93
PARVIFOLIA	*cogollo vs. argos*	0.3005	0.7129	0.0594	294.8	1652.8	0.53	0.09
PARVIFOLIA	*gloria vs. bio*	0.4460	0.4753	0.0099	510.0	342.5	0.00	0.00

#### Phylogenetic Signal of Climatic Variables

The phylogenetic signal tests (Table 4 and Supplementary Figure 7) show that the two principal components of temperature- and precipitation-related variables are best explained by different evolutionary models according to the Akaike Information Criterion (AIC), as shown in Table 4. The temperature-related variables exhibit less phylogenetic signal than the precipitation-related variables, shown by Blomberg’s K (Blomberg et al., 2003) and Pagel’s lambda (Pagel, 1999). This is true both for the SSP and the SCP (RAxML). The low values of both indicators (of phylogenetic signal of MAP) show that species of different clades converge to living at similar temperatures.

**TABLE 4:** Phylogenetic signal of temperature- and precipitation-related variables on the sequence capture phylogeny (SCP) (RAxML) and Sanger sequence phylogeny (SSP).

Variable used	Representative bio-variable	Model of best fit by AIC and AICc (SSP)	Model of best fit by AIC and AICc (SCP)	K-Statistic of phylogenetic signal (SSP)	K-Statistic of phylogenetic signal (SCP)	Pagel’s lambda (SSP)	Pagel’s lambda (SCP)
PC1_temp	Bio1	Lambda	Lambda	0.41	0.68	0.46	0.59
PC2_temp	Bio3	Ornstein-Uhlenbeck	Ornstein-Uhlenbeck	0.43	0.68	0	0.59
PC1_prec	Bio15	Brownian Motion	Early Burst	0.71	1.35	1	1
PC2_prec	Bio12	Brownian Motion	Brownian Motion	0.96	1.05	1	1

## Discussion

Our complementary taxon and sequence sampling methods allowed us to cover the different depths of the phylogenetic history of *Aiphanes.* The Sanger phylogeny (Figure 2) included more species and provided sufficient support for the inclusion of species into clades, whereas the sequence capture phylogenies (Figure 3 and Supplementary Figures 1, 2) densely sampled several poorly known species and clades that the Sanger sequence failed to resolve (i.e., the subspecies of *A. hirsuta*, the differentiation between *A. concinna* and *A. lindeniana*). Also, the Sanger sequencing phylogeny did not support the relationships between clades, whereas the sequence capture phylogeny provided a better-resolved backbone for between-clade relationships (Table 2). Therefore, we encourage this mixed approach when (1) sampling with more genome-wide methods is not available for all the targeted taxa and (2) different phylogenetic depths are relevant to the study. At the micro-macroevolutionary interphase where populations, subspecies, and species are being sampled, the Sequence Capture approach was most important. We base the following paragraphs of the current section on these complementary results. We use these phylogenetic hypotheses to discuss the roles of geographical and climatic niche evolution in fostering divergence in the genus.

### Diversification in *Aiphanes*

**FIGURE 6:**
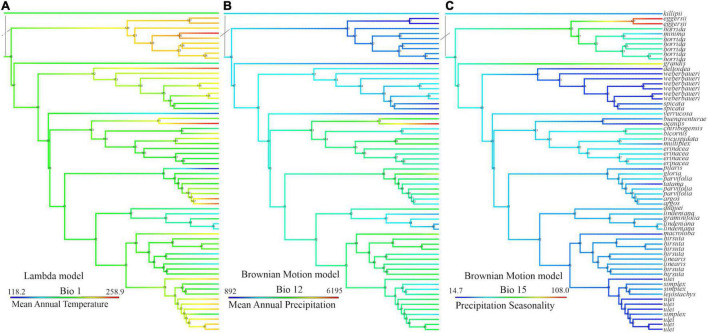
Climatic variable reconstruction according to the best-fitting model of continuous trait evolution by the Akaike Information Criterion, values reconstructed from all curated species occurrences, and CHELSA climatic layers on the Sanger sequence phylogeny (SSP); **(A)** mean annual temperature by the Lambda model, values in deciCelsius, **(B)** mean annual precipitation by Brownian Motion, values in kg m^–2^/10, and **(C)** precipitation seasonality by Brownian motion, values in percentage of monthly variation over annual mean.

The earliest diverging members of the genus (except *A. minima*) occur today in mid-elevation mesic environments (*A. killipii, A. horrida*, and *A. eggersii*). Diversification in the genus appears to have occurred recurrently in different temperatures (Figure 6A), and to have been related to the early conquest of areas with higher precipitation and lower precipitation seasonality, and the conservation of this trait (Figures 4B,C, 6B,C and Supplementary Figure 5). These precipitation-related adaptations were conserved both at higher elevations in the northern Andes (*linearis* and *lindeniana* clades) and at lower elevations in the *cis*- and *trans*-Andean lowlands (*acaulis* clade in Choco and *A. ulei* and *A. deltoidea* in the Amazon). This is shown by the high phylogenetic signal of the joint precipitation-related variables but not of the temperature-related variables reflected in the lower values of Blomberg’s K and Pagel’s Lambda for the former variable set (Table 3 and refer to Supplementary Figure 7).

Both the geographic distribution of species and clades and the climatic evolution of the genus highlight its close relationship with Andean mountain building from the origin of the genus, as shown by the character state reconstruction indicating mid-elevation temperatures from the ancestor of the genus and subsequent nodes (Figure 4). Also, only *A. hirsuta* reaches Panama and Costa Rica, and *A. minima* Burret the West Indies; the rest of the species are on the Andean Mountains or surrounding lowlands. The mean annual temperature of the nodes in *Aiphanes* is reconstructed as mild, which can be understood as mid-elevations at 1,000–2,000 m. The MAT during the late Miocene to Pliocene, when *Aiphanes* diversified (Eiserhardt et al., 2011b; this study), was probably warmer than today (Zachos et al., 2001; Hansen et al., 2013); thus we expect mild temperatures to be related to paleoelevation. Furthermore, as expected by increasing mountain building, high elevation species belong to the more nested clades of *lindeniana* and *linearis*, as well as the species *A. spicata* and *A. pilaris*.

The progression from mid to higher elevations probably follows the process of mountain building and points at mid-elevation areas in the Colombian Andes (possibly the Eastern Cordillera where *A. killipii* is found today and is near two other microendemic species from different clades, *A. graminifolia* and *A. suaita*) already available during the Miocene (Anderson et al., 2015). Despite the absence of paleoelevation constraints for the Central and Western Cordilleras, thermochronological and geomorphological considerations suggest that these Cordilleras may have also experienced exhumation and uplift since the Miocene (León et al., 2018; Noriega-Londoño et al., 2020). Nevertheless, the concentrated origin of several early-diverging species in the Eastern Cordillera suggests that this area was either uplifted or connected earlier in time. The high-elevation *A. linearis* from the Western and Eastern Cordilleras is nested in the phylogeny and is among the last to form. Diversification in the Colombian cordilleras and the Choco (the *acaulis, parvifolia, lindeniana, linearis*, and *simplex* clades), as well as tendency to gradually occupy areas with higher precipitation and less seasonality and a wide variety of temperatures, hints at how the evolution of *Aiphanes* and the North Andean chains are intertwined.

**FIGURE 7:**
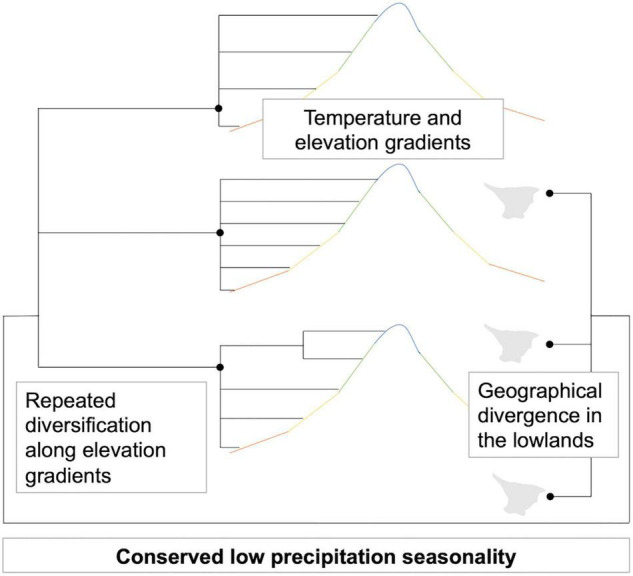
Schematic representation of how *Aiphanes* diversified.

In simple terms, the tracking of these palms of continuously moist environments allowed them to diversify, occupying different temperatures available at the places where they became geographically isolated by mountain building (Figure 7; the elevation ranges for all species are available in Supplementary Figure 8). High level of precipitation and low precipitation seasonality have been the backdrop for species persistence in the dynamic Neogene evolution of Northwestern South America (Hoorn et al., 2010), and are conserved traits in the phylogeny. On the other hand, the repeated temperature differentiation in the different clades is the result of the evolving topography, isolating species in valleys and mountains at different elevations.

Geographical divergence is important in different Andean birds (Hazzi et al., 2018) and plants (Vargas, 2018; Vargas et al., 2020) and is reflected in the low mean geographical overlap between species and their relatively small ranges. Also, it can, in some cases, be concomitant with niche adaptation (Benham and Witt, 2016). Indeed, the temperature differentiation here follows a spreading pattern shown by the parallel laddered occupation of different temperatures of same-clade species (Figure 4, refer to Bio1 occupation for clades *horrida* in light blue, *acaulis* in purple, *parvifolia* in blue, and *lindeniana* in green). Climatic space differentiation was also shown for an Andean endemic palm genus, *Ceroxylon* (Sanín et al., 2016), and Andean vertebrates (Patterson et al., 1998; Cadena et al., 2011). In the case of *Aiphanes*, topography plays a dual role by isolating populations and then truncating their potential occupation along the temperature gradient (Benham and Witt, 2016).

It is widely acknowledged that realized distributions underestimate species’ physiological tolerance (Soberon and Peterson, 2005; Feeley and Silman, 2010), and that physical barriers to dispersal might amplify this effect. The resulting physically constrained extension of species’ natural ranges enforces niche truncation (Bush et al., 2018). In *Aiphanes*, the rarely overlapping occupied temperature niches between same-clade species might be the effect of dispersal limitations affecting climatic niche realization because of, in particular, topographic barriers. Thus, our estimation of potential niche occupation, which relies on occurrences, might be ultimately biased by how well the species has succeeded in “sampling” the topographically fragmented landscape. Conversely, it is clear that the occupation of precipitation-related climatic space is much more phylogenetically constrained, as it is conserved despite geographical circumscription.

The overall pattern appears as diversification by geographical isolation in moist areas with different temperatures, which has made *Aiphanes* species-rich on the regional scale but locally rare (Borchsenius and Bernal, 1996) and locally species-poor. Ultimately, this recoils to the genus’ aforementioned paucity. Either the relative scarcity of pollinators or fluctuations in their specificity or other biotic factors, such as poor ability to recolonize areas due to poor seedling recruitment (Svenning, 1998), may have limited the population expansion of most species of *Aiphanes*. The reproductive biology of this genus has only been studied in *A. erinacea* and in *A. chiribogensis* Borchs. & Balslev, where hoverflies and gnats/midges, respectively, were reported as the main pollinators in Ecuador (Borchsenius, 1993). Our field observation is that infructescences and ripe fruits are seldom found in wild populations, but this has not been systematically assessed.

This study could be expanded in different ways. First, we lack natural history knowledge of the physiological tolerance of compound variables like energy-water balance in many tropical plant groups. Second, we lack knowledge of actual dispersal abilities mediated by biotic interactions in tropical taxa. Third, we should further understand geographical isolation: is it slope, topographic complexity, or the temporal and spatial extent of a physical barrier that is related to biodiversity patterns determined by isolation? A limitation of this study stems from these knowledge gaps.

### Concluding Remarks

As seen in the previous sections, *Aiphanes* provides an example of a diversification pattern by the occupation of the elevation gradient following high precipitation with low seasonality. Isolation into different corners of Andean relief leads to temperature differentiation the same way repeatedly across the different clades, and to high abundance of narrowly distributed species. The phylogenetic conservation of precipitation-related adaptations is coupled with possibly fundamentally larger tolerance to different temperatures. However, wider tolerance to different temperatures is not reflected in species occupation of climatic space (i.e., their realized climatic niches), because it is truncated by physical occupation of mountains and valleys from where the species are unable to disperse. This pattern might be common in Andean plant groups but understudied because of lack of taxonomic understanding, collections, sequencing, and validated distribution data for plant groups that include a high proportion of rare and narrowly distributed species.

In *Aiphanes*, future research should aim at discovering how this often-unnoticeable genus maintains populations and effective reproduction in time despite its elusive presence in mountain forests. This, combined with the expected discovery of additional microendemic species, will help to understand the evolution of this fascinating Andean gem.

## Data Availability Statement

The datasets presented in this study can be found in online repositories. The names of the repository/repositories and accession number(s) can be found below: https://www.ncbi.nlm.nih.gov/, PRJNA689999.

## Author Contributions

MS, FB, and RB designed the study. MS, FB, RB, SH-G, HM, and AC collected the data. MS, AG, FB, SC-M, YO, NA, and MP analyzed the data. MS wrote the manuscript. All authors revised, contributed, and approved the manuscript.

## Conflict of Interest

The authors declare that the research was conducted in the absence of any commercial or financial relationships that could be construed as a potential conflict of interest.

## Publisher’s Note

All claims expressed in this article are solely those of the authors and do not necessarily represent those of their affiliated organizations, or those of the publisher, the editors and the reviewers. Any product that may be evaluated in this article, or claim that may be made by its manufacturer, is not guaranteed or endorsed by the publisher.

## Footnotes

1
http://broadinstitute.github.io/picard
