# Supplementary material for: The Tracking of Moist Habitats Allowed Aiphanes (Arecaceae) to Cover the Elevation Gradient of the Northern Andes
Source: Front Plant Sci. 2022 Jun 27;13:881879. doi: 10.3389/fpls.2022.881879 (PMC9272002; doi:10.3389/fpls.2022.881879)

Supplementary Material

Supplementary Figure 1 - ASTRAL species tree phylogeny (SCPa) of Aiphanes based on 1993 independent gene trees, with quartet support values of 1.0 for clades highlighted in colors and discussed below (see Table 2 for support values for all clades). Population sampling collapsed for simplification, with number of individuals indicated in parentheses; dashed lines indicate correspondence with RAxML concatenated phylogeny (SCPc: Supplementary Figure 2). Photos show: (**A**) *Aiphanes killipii*: habit; (**B**) *A. leiostachys* (*simplex* clade): habit; (**C**) *A. cogollo* (*parvifolia* clade): habit; (**D**) *A. bio*: inflorescence; (**E**) *A. concinna* (*lindeniana* clade): habit; (**F**) *A. macroloba*: habit; (**G**) *A. hirsuta*: habit. For a comparison between the ASTRAL and the RaxML concatenated sequence phylogenies, see Supplementary Figure 2, SCPc) (Photographs: [**C**] by Alvaro Cogollo, [**D**] by Camilo Flórez, [**G**] by Felipe Mesa).

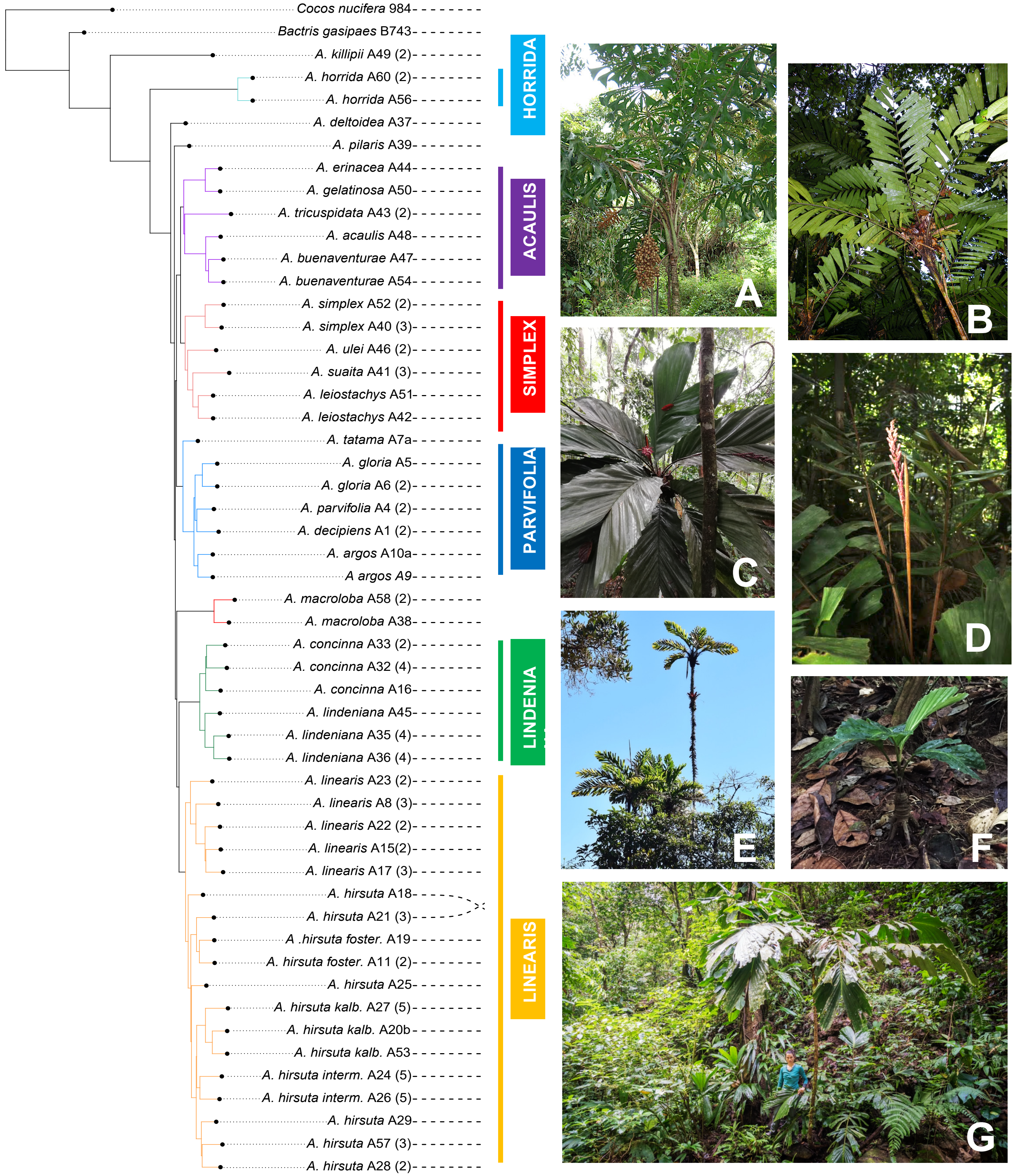

Supplement: Supplementary file 1 [file Data_Sheet_1.PDF]
