# Supplementary material for: The Tracking of Moist Habitats Allowed Aiphanes (Arecaceae) to Cover the Elevation Gradient of the Northern Andes
Source: Front Plant Sci. 2022 Jun 27;13:881879. doi: 10.3389/fpls.2022.881879 (PMC9272002; doi:10.3389/fpls.2022.881879)

Supplementary Material

Supplementary Figure 2- Contrasted (**A**) ASTRAL species tree and (**B**) RAXML concatenated sequence phylogeny of the genus *Aiphanes,* with full sampling indicated and color-coding following fig. 1; for clade support see Table 2 in main text.

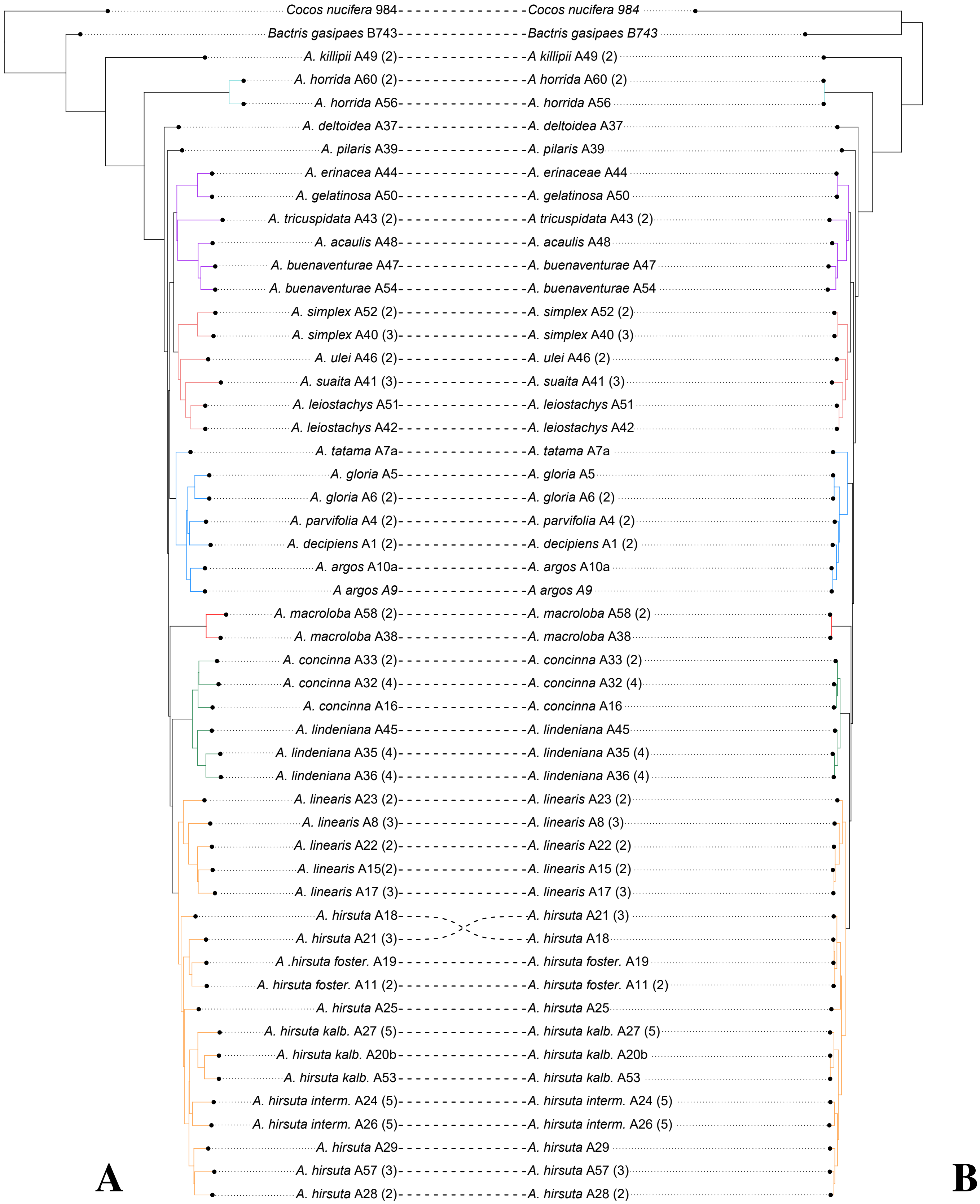

Supplement: Supplementary file 2 [file Data_Sheet_2.PDF]
