# Supplementary material for: The Tracking of Moist Habitats Allowed Aiphanes (Arecaceae) to Cover the Elevation Gradient of the Northern Andes
Source: Front Plant Sci. 2022 Jun 27;13:881879. doi: 10.3389/fpls.2022.881879 (PMC9272002; doi:10.3389/fpls.2022.881879)
Supplement: Supplementary file 3 [file Data_Sheet_3.PDF]

Supplementary Material

Supplementary Figure 3 - Occurrence points, temperature and precipitation variable Principal Component Analysis (PCA) plots for (**A, D, G**) the *acaulis* clade, (**B, E, H**) the *parvifolia* clade, (**C F, I**) the *lindeniana* clade, (**J, M, P**) the *weberbaueri* clade, (**K, N, Q**) the *simplex* clade, (**L, O, R**) the *linearis* clade, (**S, T, U**) the *horrida* clade; occurence points of (**V**) *A. weberbaueri*, (**W**) *A. suaita*, (**X**) *A. pilaris*, (**Y**) *A. macroloba*. In the cases where there are seemingly fewer points in the maps than in the PCA, this is due to point overlap.

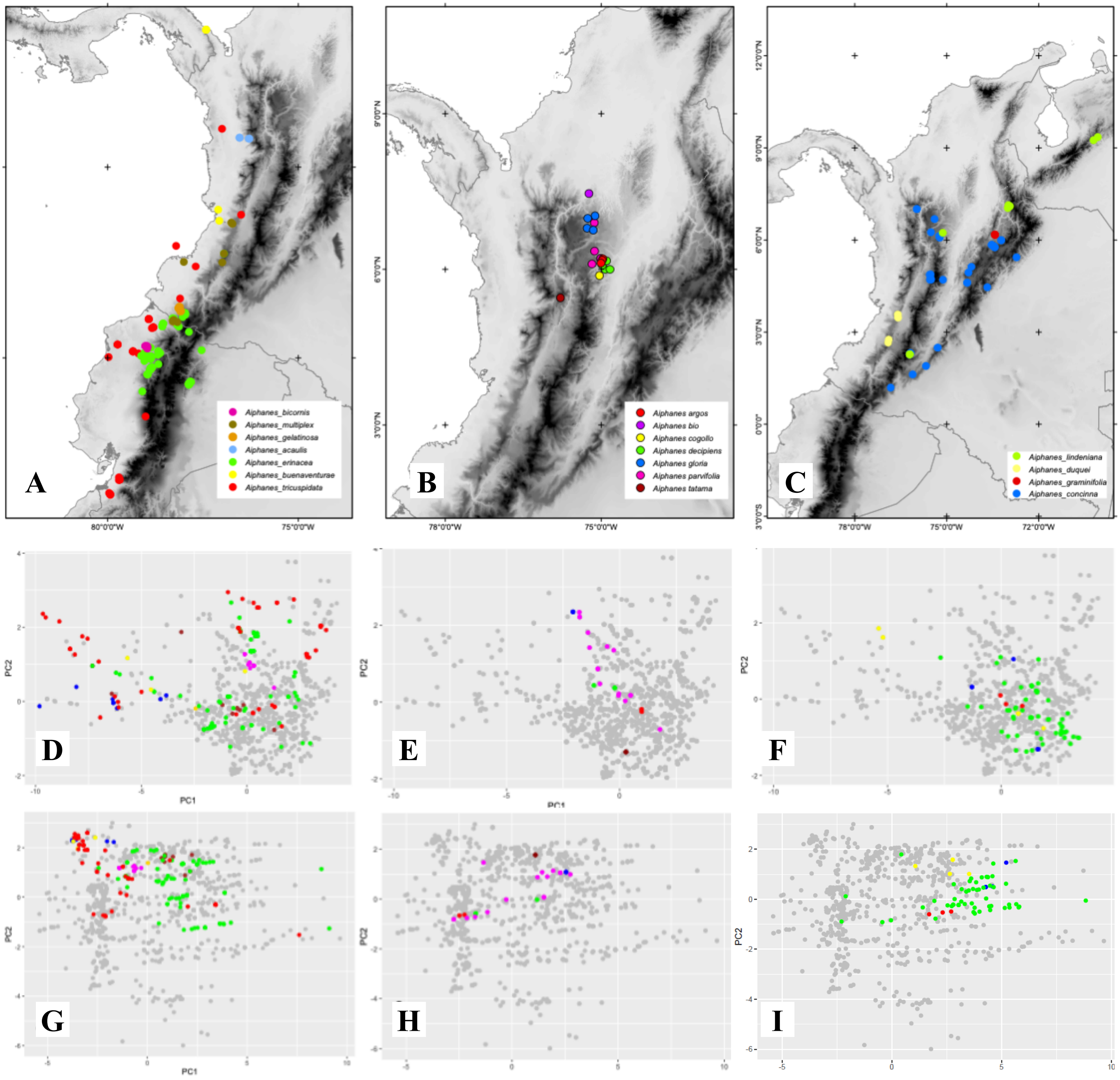

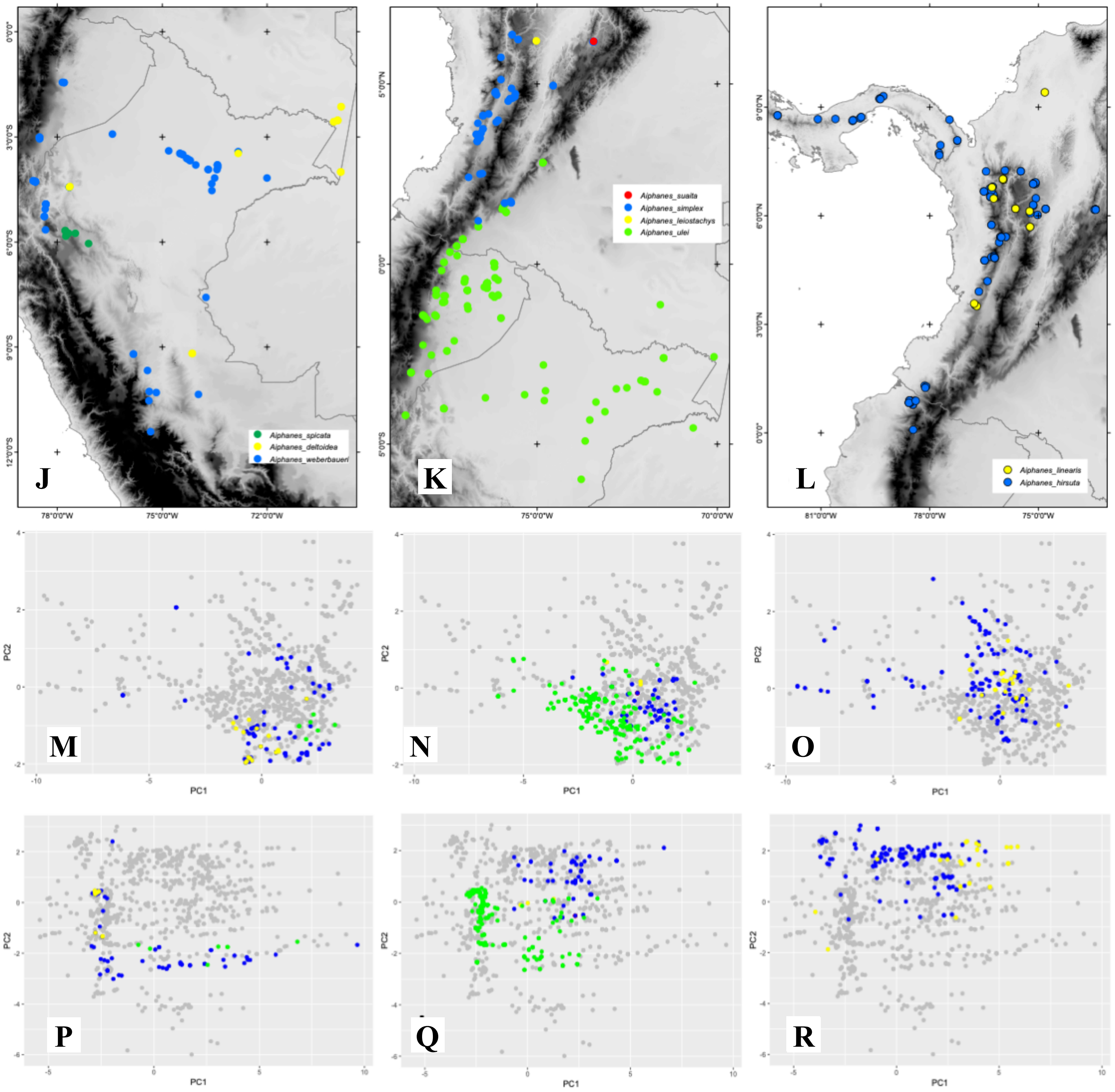
J
Aiphanes_spicata
Aiphanes_deltoidea
Aiphanes_weberbaueri
K
Aiphanes_suaita
Aiphanes_simplex
Aiphanes_leiostachys
Aiphanes_ulei
L
Aiphanes_linearis
Aiphanes_hirsuta
M
N
O
P
Q
R
PC1
PC2

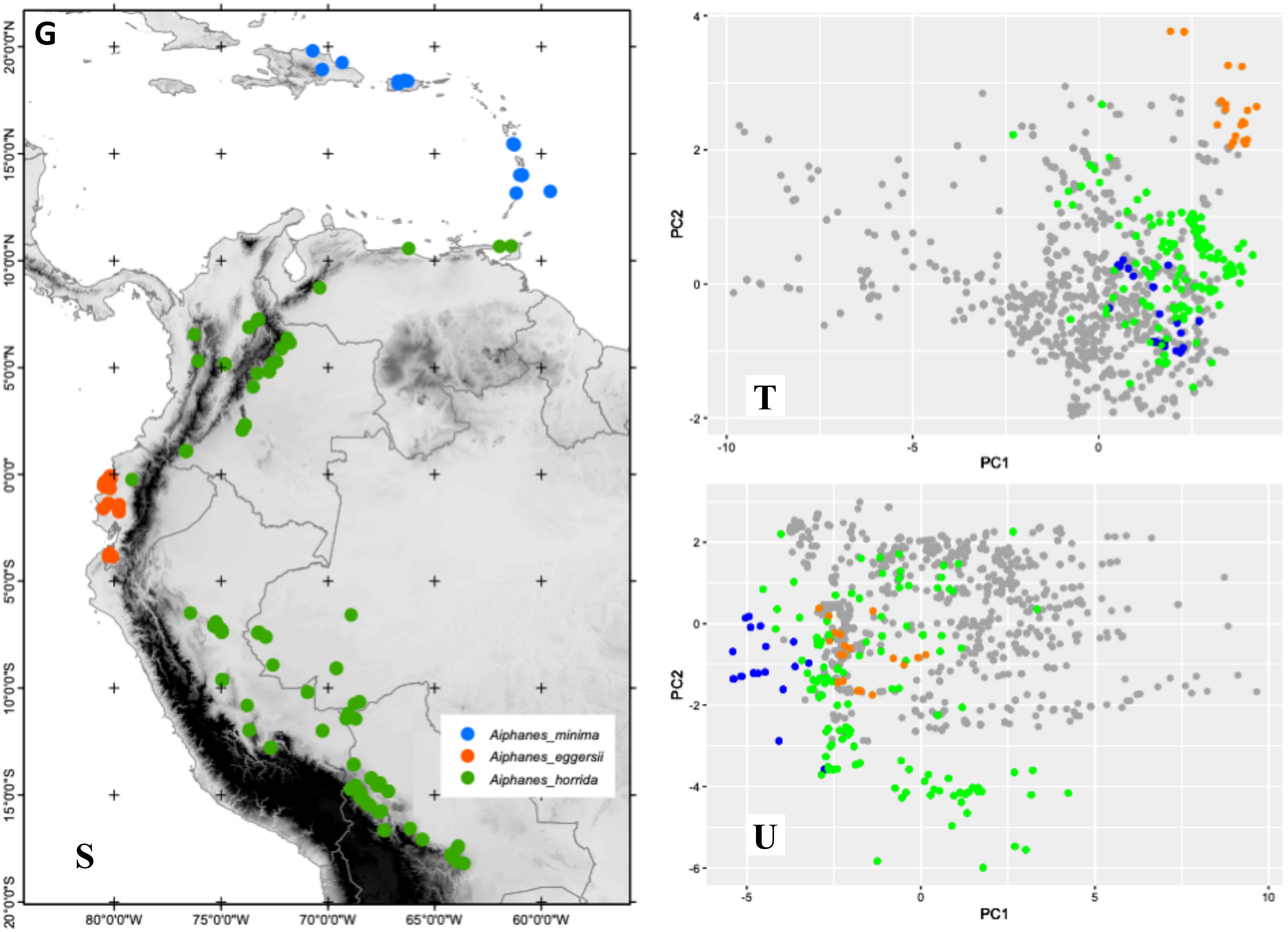

G
S
T
U
Aiphanes_minima
Aiphanes_eggersii
Aiphanes_horrida
20°0'0"N
15°0'0"N
10°0'0"N
5°0'0"N
0°0'0"
5°0'0"S
10°0'0"S
15°0'0"S
20°0'0"S
80°0'0"W
75°0'0"W
70°0'0"W
65°0'0"W
60°0'0"W
PC1
PC2

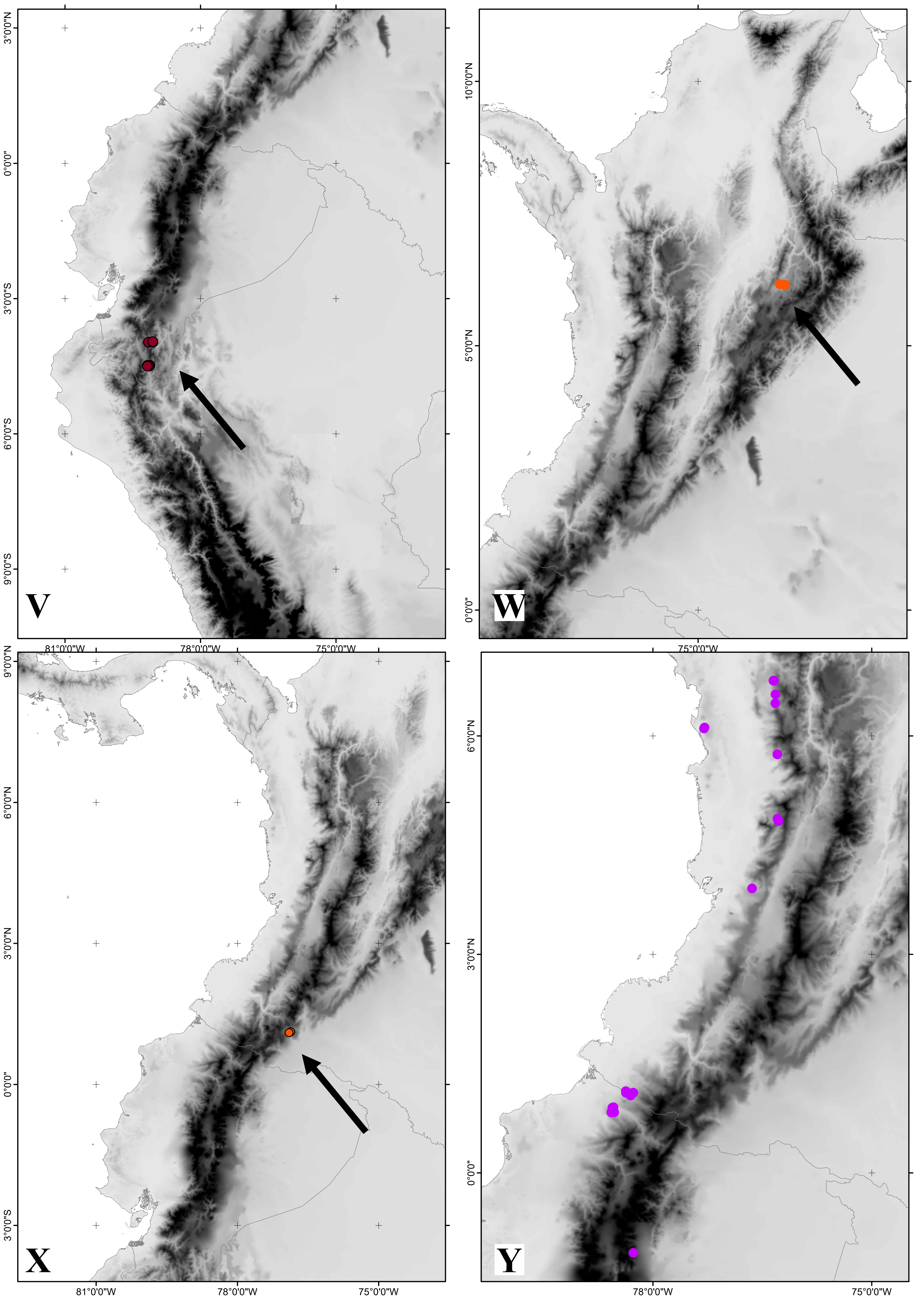
3°0'0"N
0°0'0"
3°0'0"S
6°0'0"S
9°0'0"S
V
81°0'0"W
78°0'0"W
75°0'0"W
10°0'0"N
5°0'0"N
0°0'0"
W
75°0'0"W
9°0'0"N
6°0'0"N
3°0'0"N
0°0'0"
3°0'0"S
X
81°0'0"W
78°0'0"W
75°0'0"W
6°0'0"N
3°0'0"N
0°0'0"
Y
78°0'0"W
75°0'0"W
