# Supplementary material for: The Tracking of Moist Habitats Allowed Aiphanes (Arecaceae) to Cover the Elevation Gradient of the Northern Andes
Source: Front Plant Sci. 2022 Jun 27;13:881879. doi: 10.3389/fpls.2022.881879 (PMC9272002; doi:10.3389/fpls.2022.881879)
Supplement: Supplementary file 4 [file Data_Sheet_4.PDF]

Supplementary Material

Supplementary Figure 4 - Binarized Species Distribution Models for all species and clades (70% probability threshold and less than 15% data omission, clade assignments for species are indicated by the colored bars on top of the models).

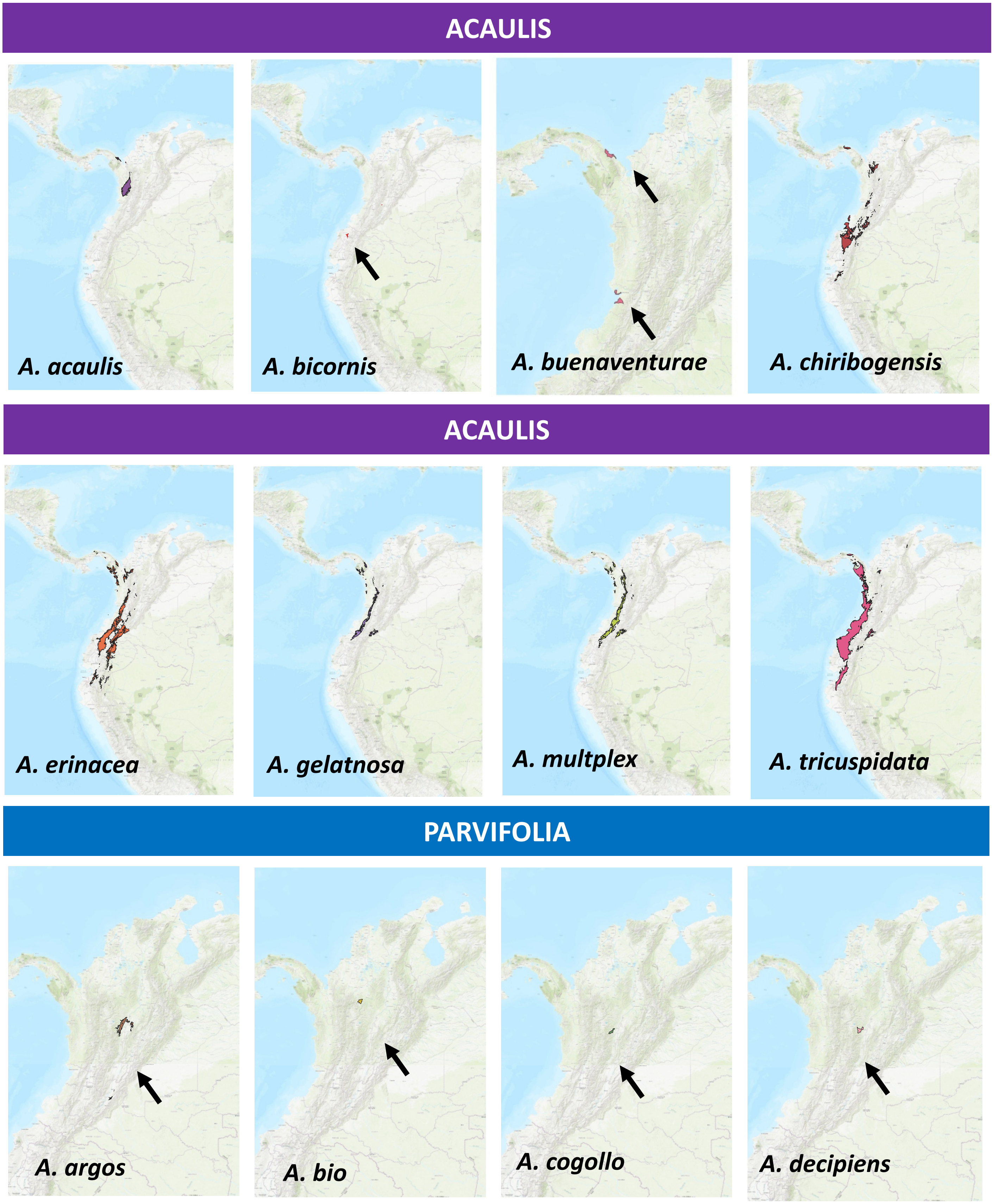

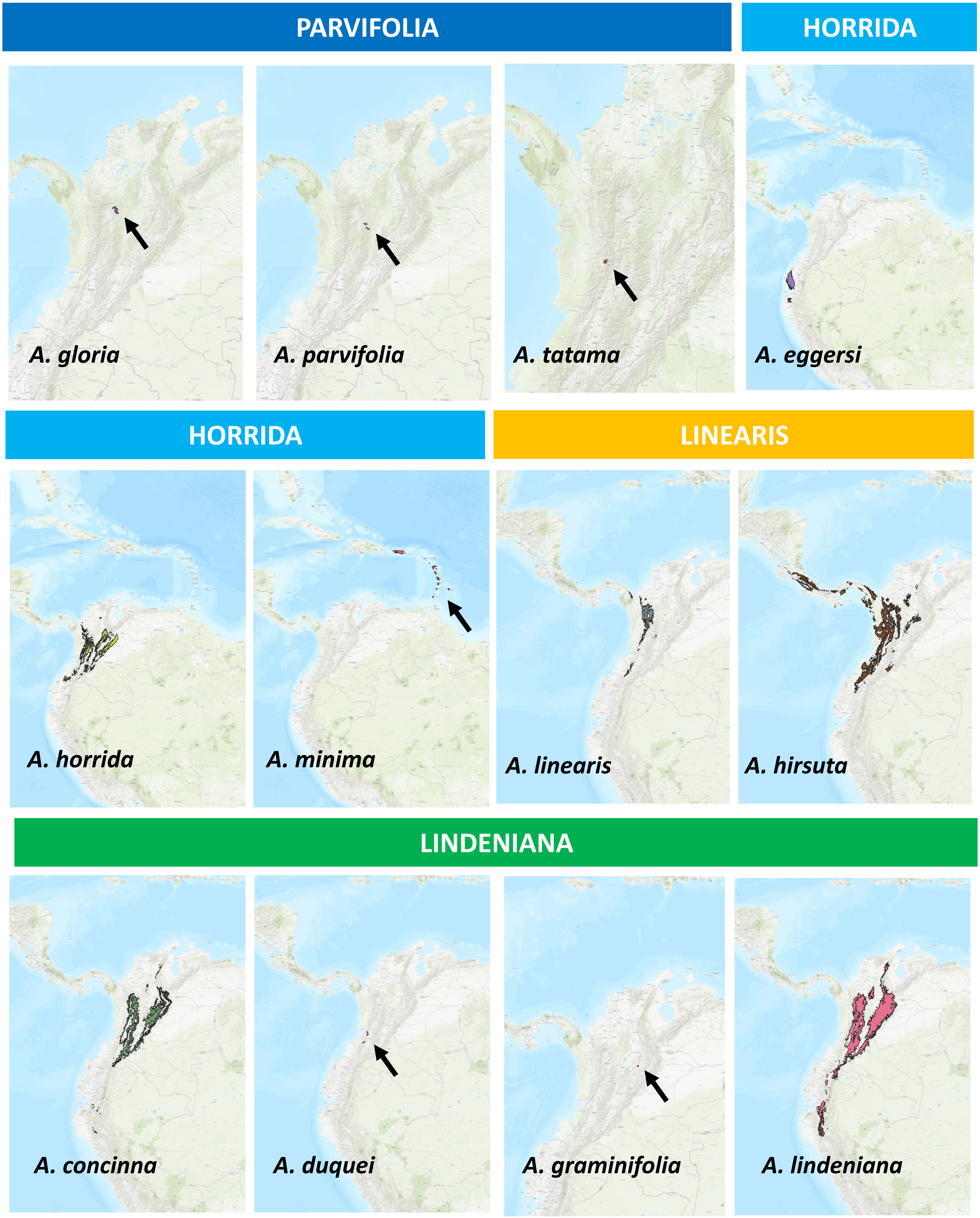
PARVIFOLIA
HORRIDA
A. gloria
A. parvifolia
A. tatama
A. eggersi
HORRIDA
LINEARIS
A. horrida
A. minima
A. linearis
A. hirsuta
LINDENIANA
A. concinna
A. duquei
A. graminifolia
A. lindeniana

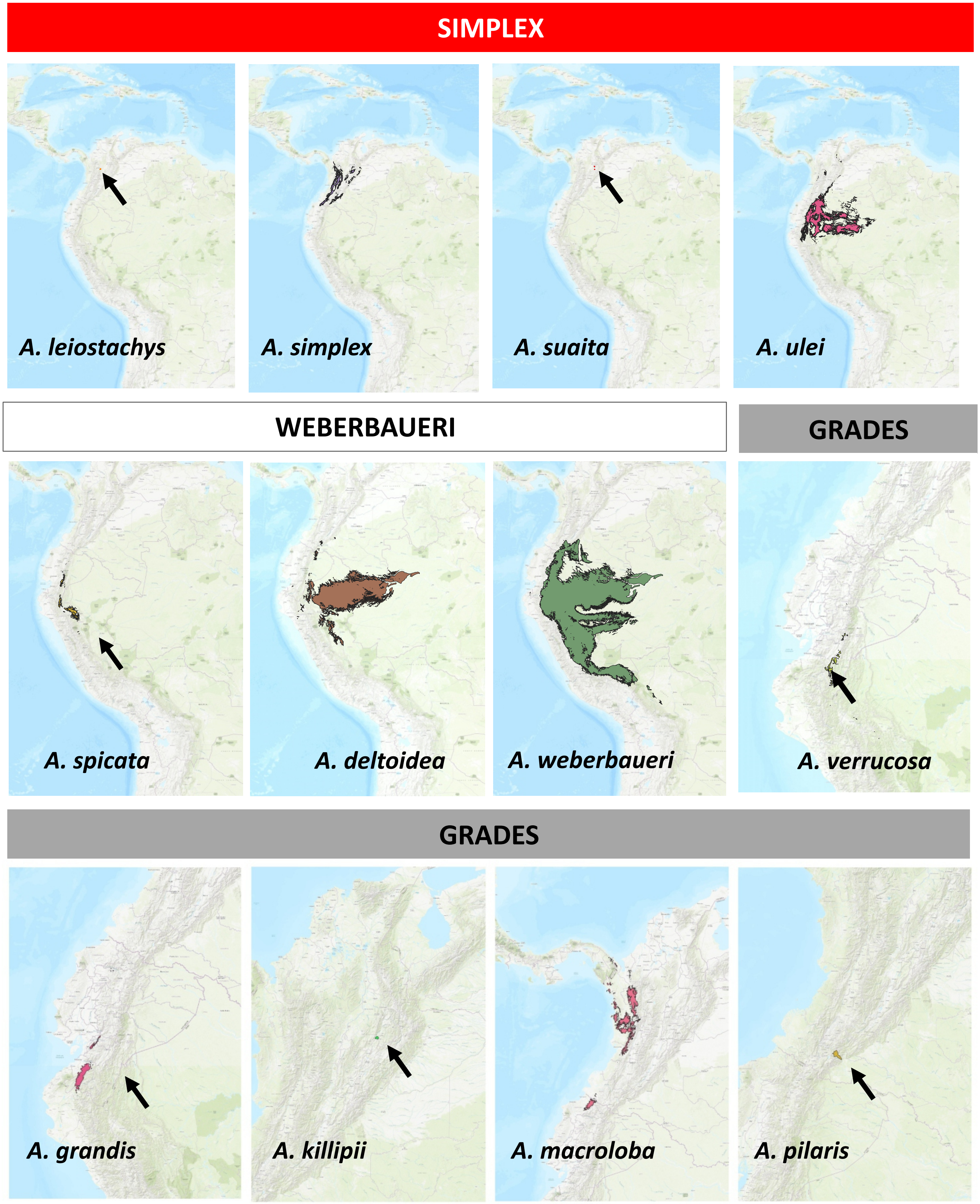
SIMPLEX
A. leiostachys
A. simplex
A. suaita
A. ulei
WEBERBAUERI
GRADES
A. spicata
A. deltoidea
A. weberbaueri
A. verrucosa
GRADES
A. grandis
A. killipii
A. macroloba
A. pilaris
