# Supplementary material for: The Tracking of Moist Habitats Allowed Aiphanes (Arecaceae) to Cover the Elevation Gradient of the Northern Andes
Source: Front Plant Sci. 2022 Jun 27;13:881879. doi: 10.3389/fpls.2022.881879 (PMC9272002; doi:10.3389/fpls.2022.881879)
Supplement: Supplementary file 5 [file Data_Sheet_5.PDF]

Supplementary Material

Supplementary Figure 5 - Ancestral state reconstruction of four independent Bioclimatic continuous states on the Sanger Sequence Phylogeny (**A-D**) and Sequence Capture Phylogeny (**E-H**). Each variable was reconstructed on tree by the best fitting evolutionary model by the AIC and AICc criteria. The biovariables and evolutionary models are indicated on each plot, as well as the variable's relatedness to each of the Principal Components of the PCA analysis from the Supplementary Figure 7.

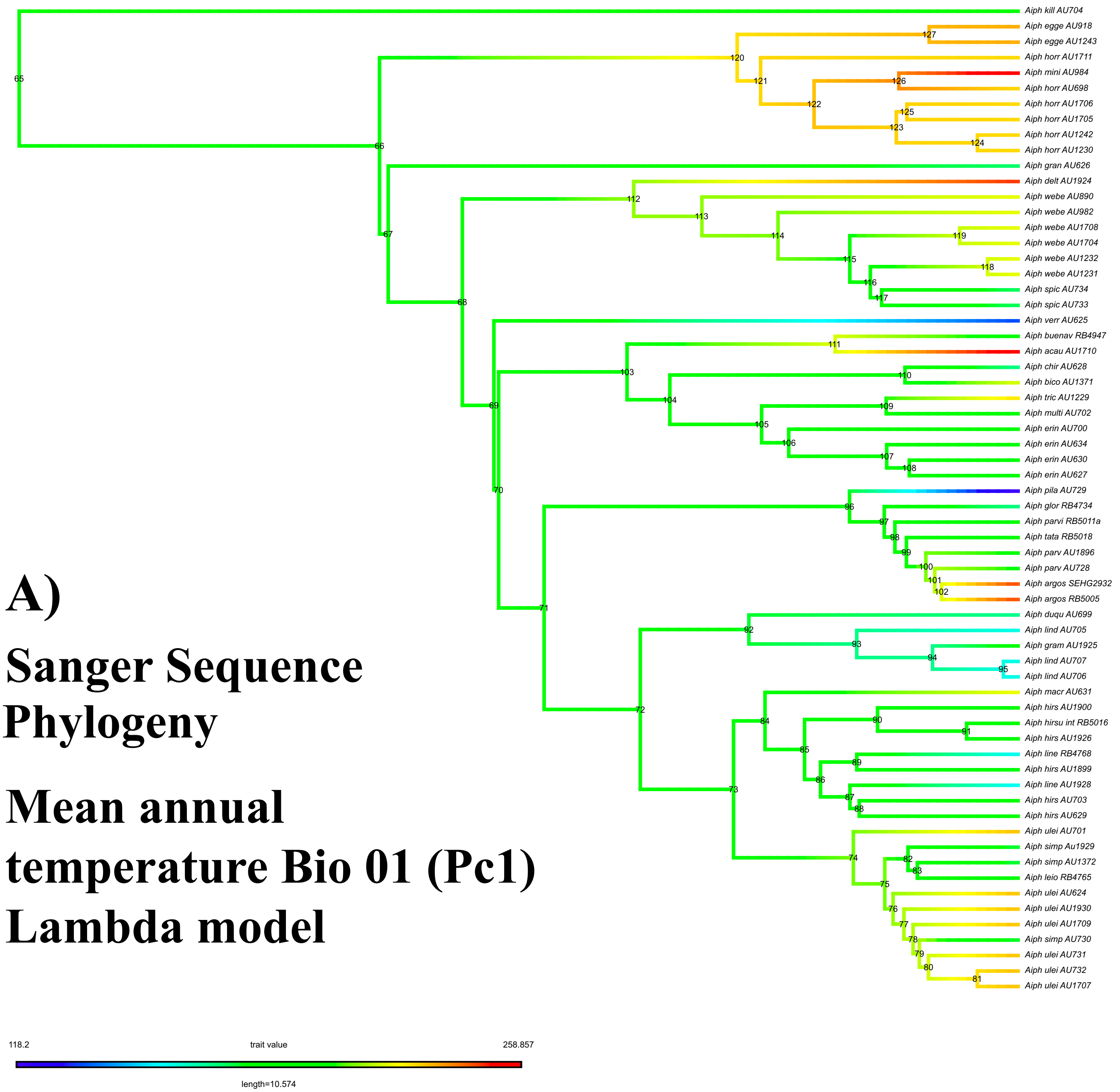

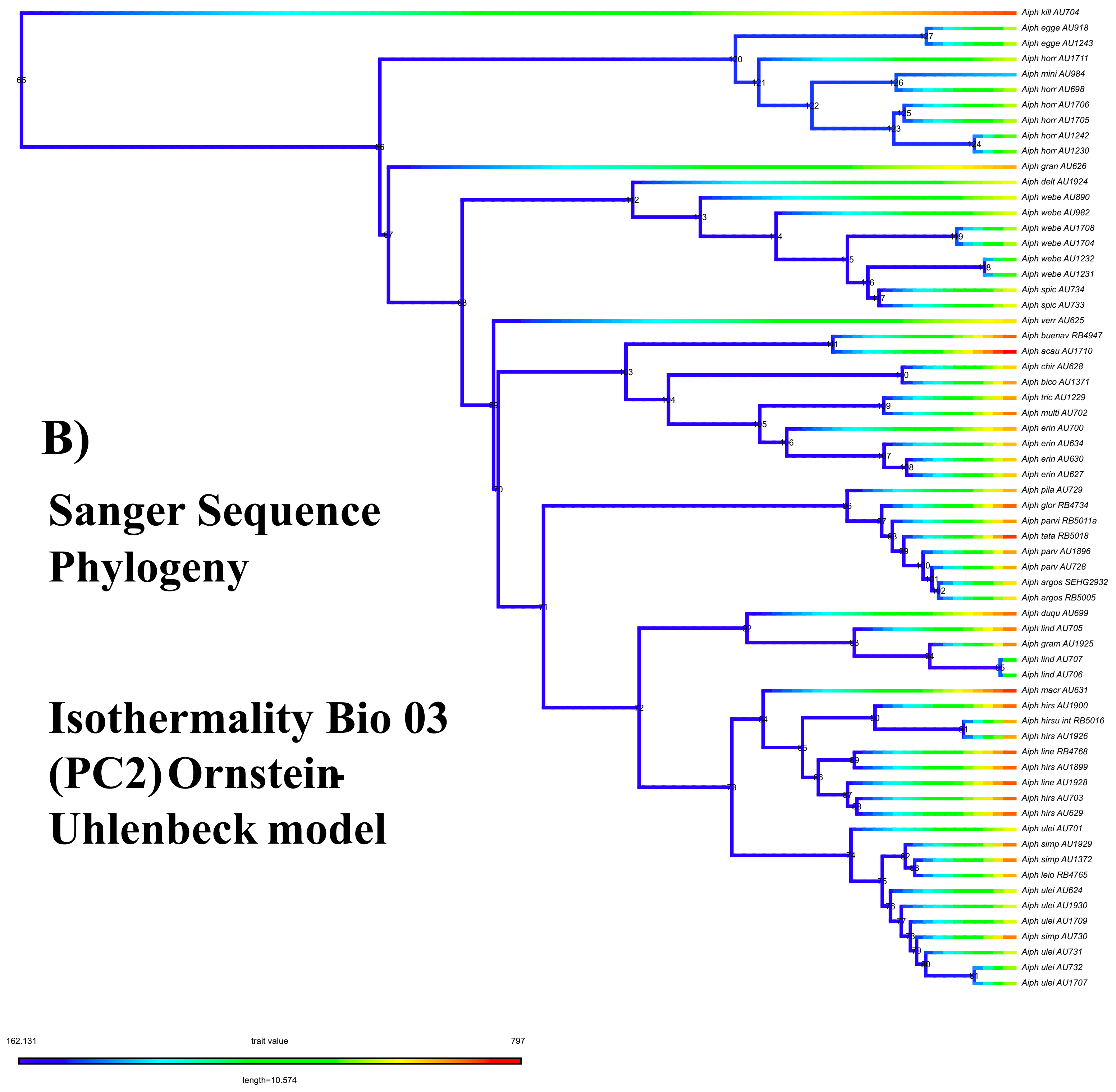
B)
Sanger Sequence Phylogeny
Isothermality Bio 03 (PC2) Ornstein-Uhlenbeck model
Aiph kill AU704
Aiph egge AU918
Aiph egge AU1243
Aiph horr AU1711
Aiph mini AU984
Aiph horr AU698
Aiph horr AU1706
Aiph horr AU1705
Aiph horr AU1242
Aiph horr AU1230
Aiph gran AU626
Aiph delt AU1924
Aiph webe AU890
Aiph webe AU982
Aiph webe AU1708
Aiph webe AU1704
Aiph webe AU1232
Aiph webe AU1231
Aiph spic AU734
Aiph spic AU733
Aiph verr AU625
Aiph buenav RB4947
Aiph acau AU1710
Aiph chir AU628
Aiph bico AU1371
Aiph tric AU1229
Aiph multi AU702
Aiph erin AU700
Aiph erin AU634
Aiph erin AU630
Aiph erin AU627
Aiph pila AU729
Aiph glor RB4734
Aiph parvi RB5011a
Aiph tata RB5018
Aiph parv AU1896
Aiph parv AU728
Aiph argos SEHG2932
Aiph argos RB5005
Aiph duqu AU699
Aiph lind AU705
Aiph gram AU1925
Aiph lind AU707
Aiph lind AU706
Aiph macr AU631
Aiph hirs AU1900
Aiph hirsu int RB5016
Aiph hirs AU1926
Aiph line RB4768
Aiph hirs AU1899
Aiph line AU1928
Aiph hirs AU703
Aiph hirs AU629
Aiph ulei AU701
Aiph simp AU1929
Aiph simp AU1372
Aiph leio RB4765
Aiph ulei AU624
Aiph ulei AU1930
Aiph ulei AU1709
Aiph simp AU730
Aiph ulei AU731
Aiph ulei AU732
Aiph ulei AU1707
162.131
trait value
797
length=10.574

C)

Sanger Sequence Phylogeny

Annual Precipitation Bio 12 (Pc2) Brownian Motion model

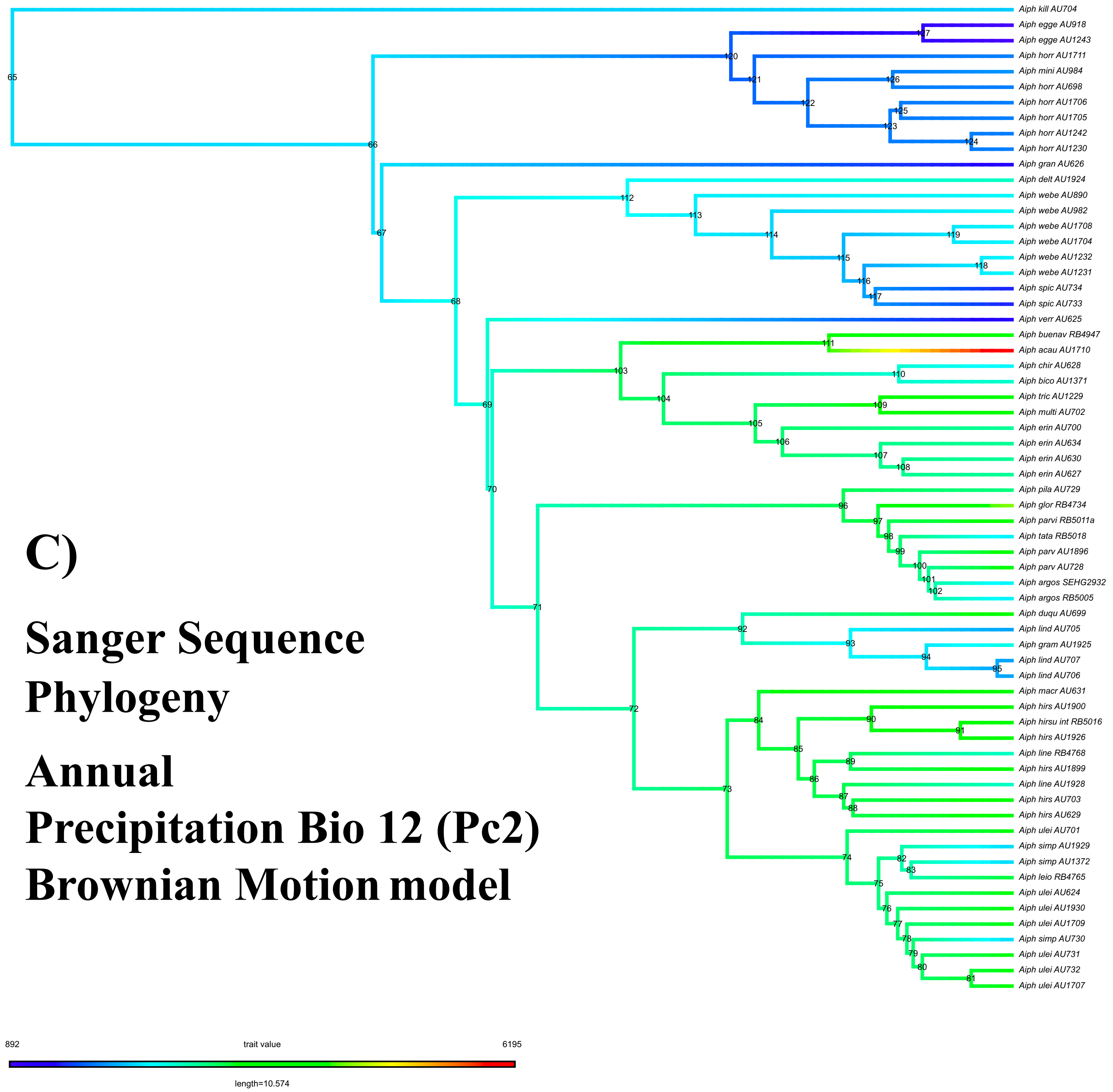

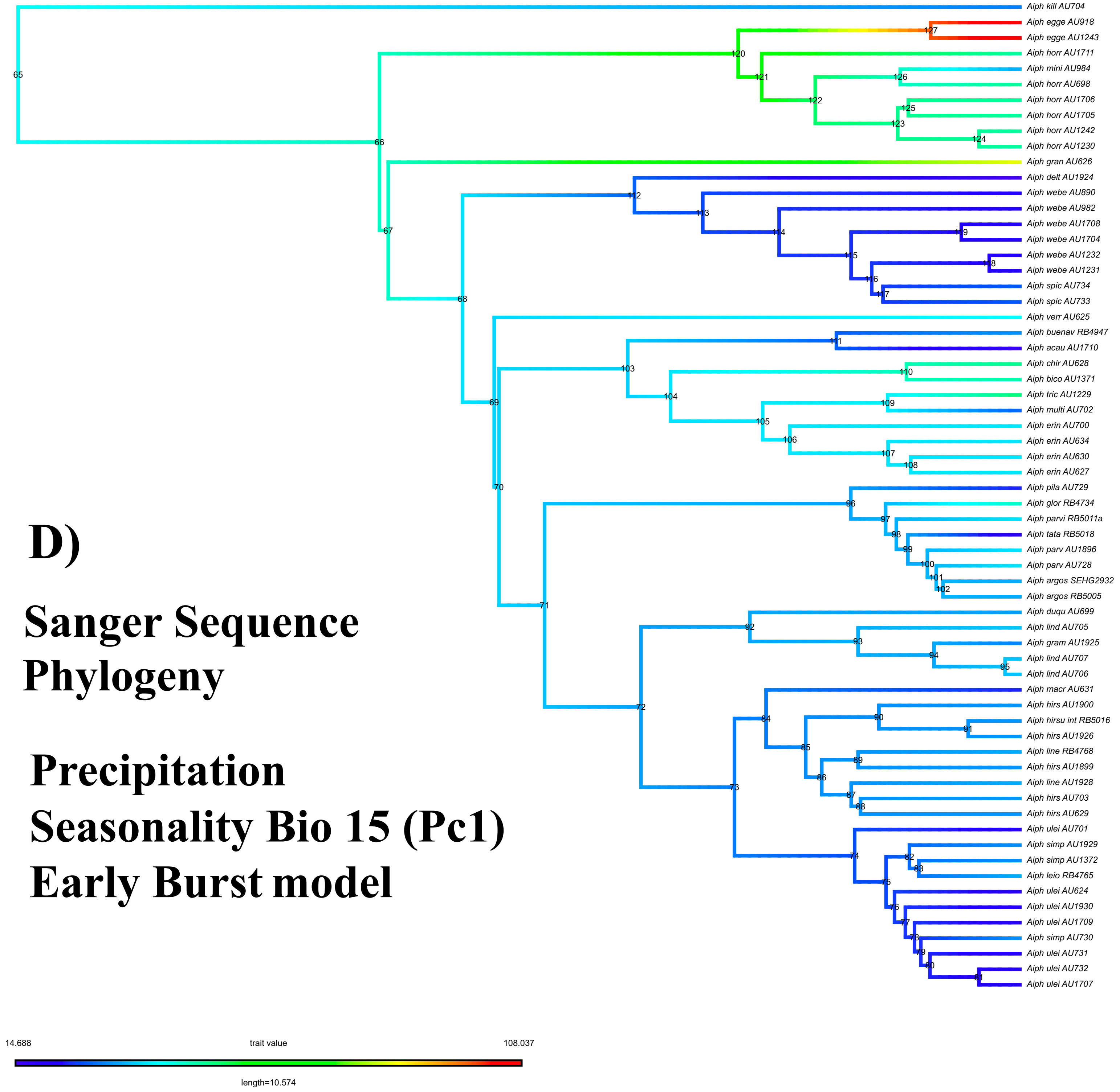
D)
Sanger Sequence Phylogeny
Precipitation Seasonality Bio 15 (Pc1) Early Burst model
Aiph kill AU704
Aiph egge AU918
Aiph egge AU1243
Aiph horr AU1711
Aiph mini AU984
Aiph horr AU698
Aiph horr AU1706
Aiph horr AU1705
Aiph horr AU1242
Aiph horr AU1230
Aiph gran AU626
Aiph delt AU1924
Aiph webe AU890
Aiph webe AU982
Aiph webe AU1708
Aiph webe AU1704
Aiph webe AU1232
Aiph webe AU1231
Aiph spic AU734
Aiph spic AU733
Aiph verr AU625
Aiph buenav RB4947
Aiph acau AU1710
Aiph chir AU628
Aiph bico AU1371
Aiph tric AU1229
Aiph multi AU702
Aiph erin AU700
Aiph erin AU634
Aiph erin AU630
Aiph erin AU627
Aiph pila AU729
Aiph glor RB4734
Aiph parvi RB5011a
Aiph tata RB5018
Aiph parv AU1896
Aiph parv AU728
Aiph argos SEHG2932
Aiph argos RB5005
Aiph duqu AU699
Aiph lind AU705
Aiph gram AU1925
Aiph lind AU707
Aiph lind AU706
Aiph macr AU631
Aiph hirs AU1900
Aiph hirsu int RB5016
Aiph hirs AU1926
Aiph line RB4768
Aiph hirs AU1899
Aiph line AU1928
Aiph hirs AU703
Aiph hirs AU629
Aiph ulei AU701
Aiph simp AU1929
Aiph simp AU1372
Aiph leio RB4765
Aiph ulei AU624
Aiph ulei AU1930
Aiph ulei AU1709
Aiph simp AU730
Aiph ulei AU731
Aiph ulei AU732
Aiph ulei AU1707
14.688
trait value
108.037
length=10.574

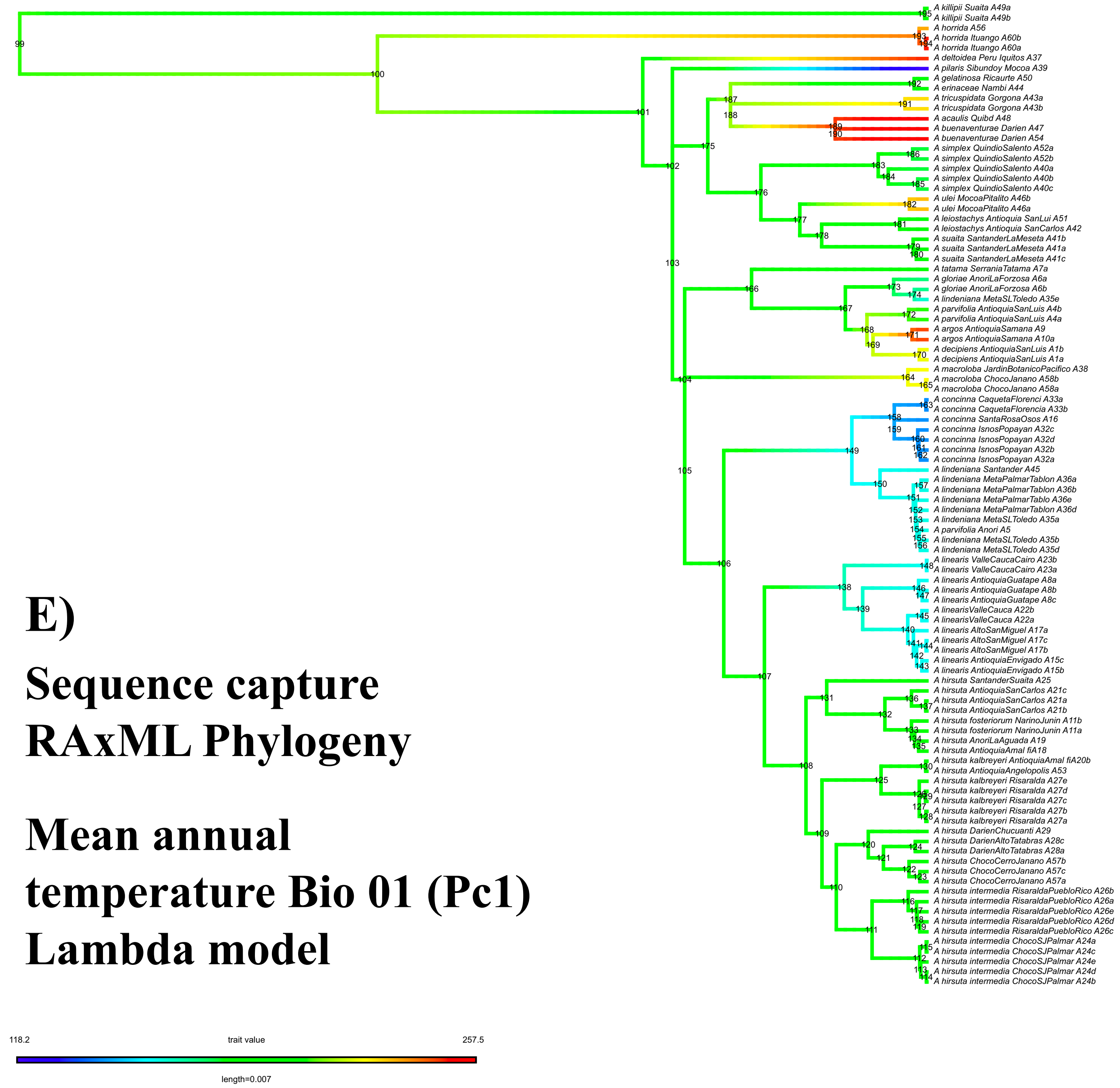

E)

Sequence capture RAxML Phylogeny

Mean annual temperature Bio 01 (Pc1) Lambda model

F)

Sequence capture RAxML Phylogeny

Isothermality Bio 03 (Pc2) Ornstein-Uhlenbeck model

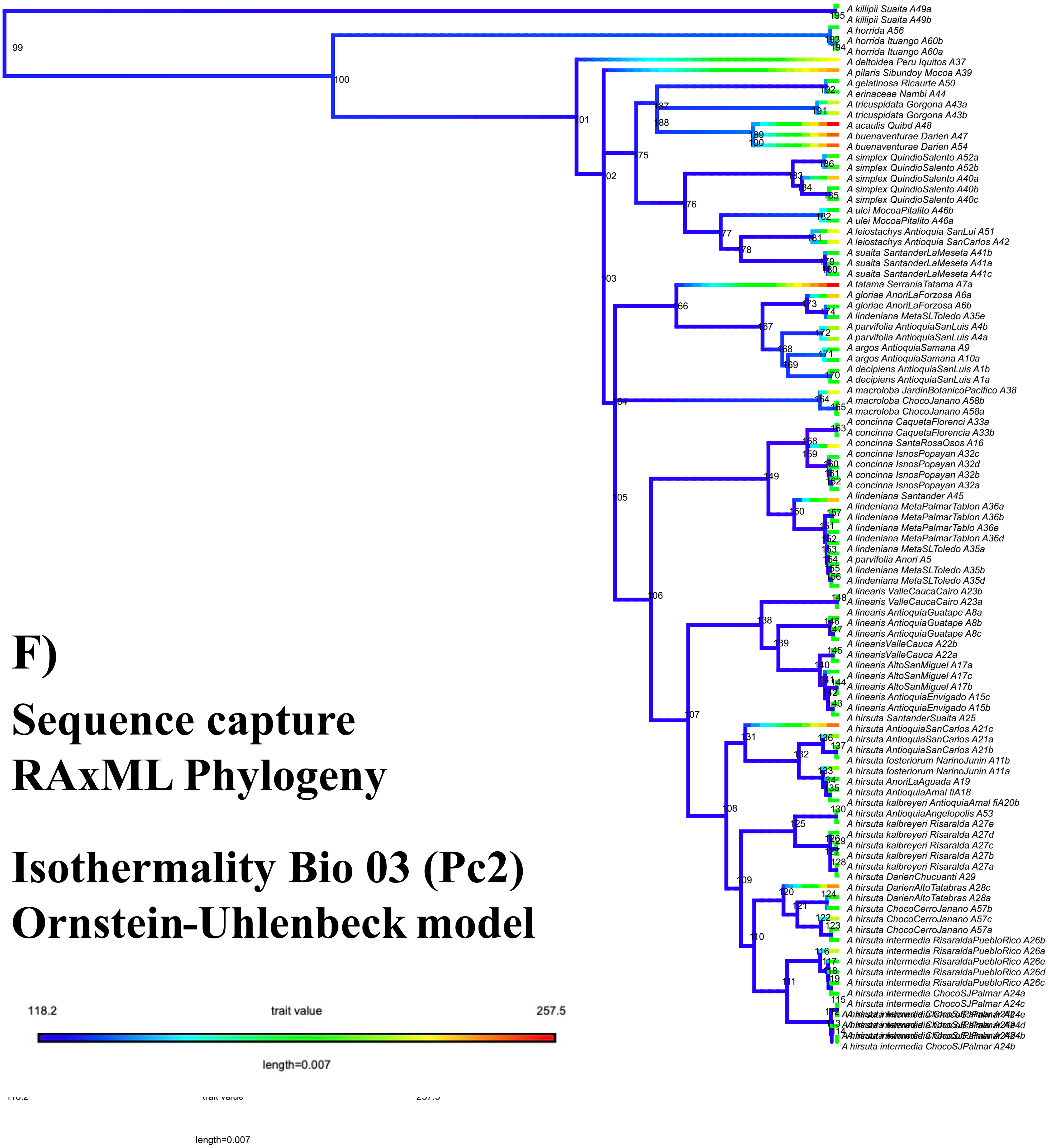

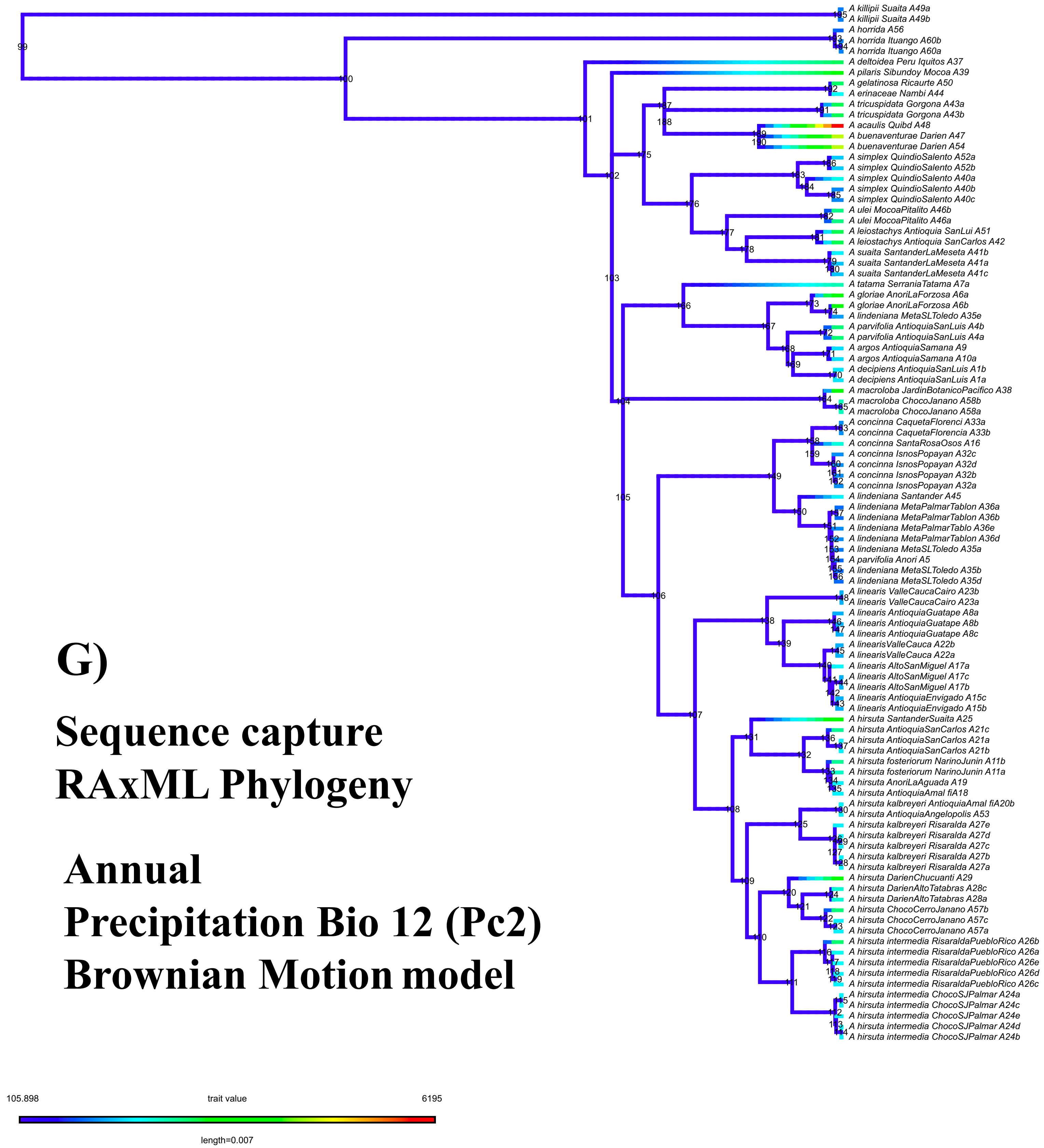
A killipii Suaita A49a
A killipii Suaita A49b
A horrida A56
A horrida Ituango A60b
A horrida Ituango A60a
A deltoidea Peru Iquitos A37
A pilaris Sibundoy Mocoa A39
A gelatinosa Ricaurte A50
A erinaceae Nambi A44
A tricuspidata Gorgona A43a
A tricuspidata Gorgona A43b
A acaulis Quibd A48
A buenaventurae Darien A47
A buenaventurae Darien A54
A simplex QuindioSalento A52a
A simplex QuindioSalento A52b
A simplex QuindioSalento A40a
A simplex QuindioSalento A40b
A simplex QuindioSalento A40c
A ulei MocoaPitalito A46b
A ulei MocoaPitalito A46a
A leiostachys Antioquia SanLui A51
A leiostachys Antioquia SanCarlos A42
A suaita SantanderLaMeseta A41b
A suaita SantanderLaMeseta A41a
A suaita SantanderLaMeseta A41c
A tatama SerraniaTatama A7a
A gloriae AnoriLaForzosa A6a
A gloriae AnoriLaForzosa A6b
A lindeniana MetaSLToledo A35e
A parvifolia AntioquiaSanLuis A4b
A parvifolia AntioquiaSanLuis A4a
A argos AntioquiaSamana A9
A argos AntioquiaSamana A10a
A decipiens AntioquiaSanLuis A1b
A decipiens AntioquiaSanLuis A1a
A macroloba JardinBotanicoPacifico A38
A macroloba ChocoJanano A58b
A macroloba ChocoJanano A58a
A concinna CaquetaFlorenci A33a
A concinna CaquetaFlorencia A33b
A concinna SantaRosaOsos A16
A concinna IsnosPopayan A32c
A concinna IsnosPopayan A32d
A concinna IsnosPopayan A32b
A concinna IsnosPopayan A32a
A lindeniana Santander A45
A lindeniana MetaPalmarTablon A36a
A lindeniana MetaPalmarTablon A36b
A lindeniana MetaPalmarTablo A36e
A lindeniana MetaPalmarTablon A36d
A lindeniana MetaSLToledo A35a
A parvifolia Anori A5
A lindeniana MetaSLToledo A35b
A lindeniana MetaSLToledo A35d
A linearis ValleCaucaCairo A23b
A linearis ValleCaucaCairo A23a
A linearis AntioquiaGuatape A8a
A linearis AntioquiaGuatape A8b
A linearis AntioquiaGuatape A8c
A linearisValleCauca A22b
A linearisValleCauca A22a
A linearis AltoSanMiguel A17a
A linearis AltoSanMiguel A17c
A linearis AltoSanMiguel A17b
A linearis AntioquiaEnvigado A15c
A linearis AntioquiaEnvigado A15b
A hirsuta SantanderSuaita A25
A hirsuta AntioquiaSanCarlos A21c
A hirsuta AntioquiaSanCarlos A21a
A hirsuta AntioquiaSanCarlos A21b
A hirsuta fosteriorum NarinoJunin A11b
A hirsuta fosteriorum NarinoJunin A11a
A hirsuta AnoriLaAguada A19
A hirsuta AntioquiaAmal fiA18
A hirsuta kalbreyeri AntioquiaAmal fiA20b
A hirsuta AntioquiaAngelopolis A53
A hirsuta kalbreyeri Risaralda A27e
A hirsuta kalbreyeri Risaralda A27d
A hirsuta kalbreyeri Risaralda A27c
A hirsuta kalbreyeri Risaralda A27b
A hirsuta kalbreyeri Risaralda A27a
A hirsuta DarienChucuanti A29
A hirsuta DarienAltoTatabras A28c
A hirsuta DarienAltoTatabras A28a
A hirsuta ChocoCerroJanano A57b
A hirsuta ChocoCerroJanano A57c
A hirsuta ChocoCerroJanano A57a
A hirsuta intermedia RisaraldaPuebloRico A26b
A hirsuta intermedia RisaraldaPuebloRico A26a
A hirsuta intermedia RisaraldaPuebloRico A26e
A hirsuta intermedia RisaraldaPuebloRico A26d
A hirsuta intermedia RisaraldaPuebloRico A26c
A hirsuta intermedia ChocoSJPalmar A24a
A hirsuta intermedia ChocoSJPalmar A24c
A hirsuta intermedia ChocoSJPalmar A24e
A hirsuta intermedia ChocoSJPalmar A24d
A hirsuta intermedia ChocoSJPalmar A24b
G)
Sequence capture
RAxML Phylogeny
Annual
Precipitation Bio 12 (Pc2)
Brownian Motion model
105.898
trait value
6195
length=0.007

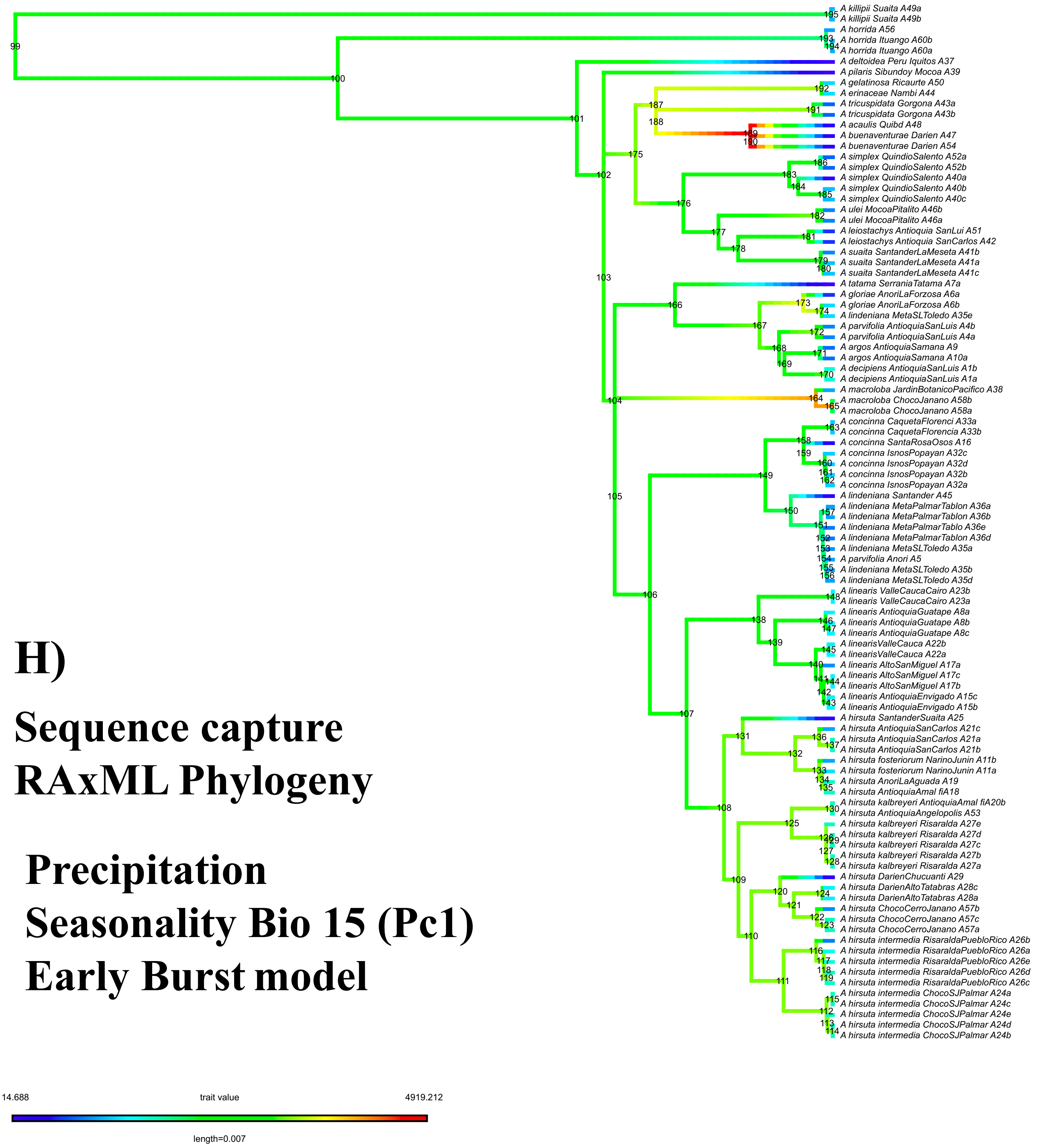
H)
Sequence capture
RAxML Phylogeny
Precipitation
Seasonality Bio 15 (Pc1)
Early Burst model
A killipii Suaita A49a
A killipii Suaita A49b
A horrida A56
A horrida Ituango A60b
A horrida Ituango A60a
A deltoidea Peru Iquitos A37
A pilaris Sibundoy Mocoa A39
A gelatinosa Ricaurte A50
A erinaceae Nambi A44
A tricuspidata Gorgona A43a
A tricuspidata Gorgona A43b
A acaulis Quibd A48
A buenaventurae Darien A47
A buenaventurae Darien A54
A simplex QuindioSalento A52a
A simplex QuindioSalento A52b
A simplex QuindioSalento A40a
A simplex QuindioSalento A40b
A simplex QuindioSalento A40c
A ulei MocoaPitalito A46b
A ulei MocoaPitalito A46a
A leiostachys Antioquia SanLui A51
A leiostachys Antioquia SanCarlos A42
A suaita SantanderLaMeseta A41b
A suaita SantanderLaMeseta A41a
A suaita SantanderLaMeseta A41c
A tatama SerraniaTatama A7a
A gloriae AnoriLaForzosa A6a
A gloriae AnoriLaForzosa A6b
A lindeniana MetaSLToledo A35e
A parvifolia AntioquiaSanLuis A4b
A parvifolia AntioquiaSanLuis A4a
A argos AntioquiaSamana A9
A argos AntioquiaSamana A10a
A decipiens AntioquiaSanLuis A1b
A decipiens AntioquiaSanLuis A1a
A macroloba JardinBotanicoPacifico A38
A macroloba ChocoJanano A58b
A macroloba ChocoJanano A58a
A concinna CaquetaFlorenci A33a
A concinna CaquetaFlorencia A33b
A concinna SantaRosaOsos A16
A concinna IsnosPopayan A32c
A concinna IsnosPopayan A32d
A concinna IsnosPopayan A32b
A concinna IsnosPopayan A32a
A lindeniana Santander A45
A lindeniana MetaPalmarTablon A36a
A lindeniana MetaPalmarTablon A36b
A lindeniana MetaPalmarTablo A36e
A lindeniana MetaPalmarTablon A36d
A lindeniana MetaSLToledo A35a
A parvifolia Anori A5
A lindeniana MetaSLToledo A35b
A lindeniana MetaSLToledo A35d
A linearis ValleCaucaCairo A23b
A linearis ValleCaucaCairo A23a
A linearis AntioquiaGuatape A8a
A linearis AntioquiaGuatape A8b
A linearis AntioquiaGuatape A8c
A linearisValleCauca A22b
A linearisValleCauca A22a
A linearis AltoSanMiguel A17a
A linearis AltoSanMiguel A17c
A linearis AltoSanMiguel A17b
A linearis AntioquiaEnvigado A15c
A linearis AntioquiaEnvigado A15b
A hirsuta SantanderSuaita A25
A hirsuta AntioquiaSanCarlos A21c
A hirsuta AntioquiaSanCarlos A21a
A hirsuta AntioquiaSanCarlos A21b
A hirsuta fosteriorum NarinoJunin A11b
A hirsuta fosteriorum NarinoJunin A11a
A hirsuta AnoriLaAguada A19
A hirsuta AntioquiaAmal fiA18
A hirsuta kalbreyeri AntioquiaAmal fiA20b
A hirsuta AntioquiaAngelopolis A53
A hirsuta kalbreyeri Risaralda A27e
A hirsuta kalbreyeri Risaralda A27d
A hirsuta kalbreyeri Risaralda A27c
A hirsuta kalbreyeri Risaralda A27b
A hirsuta kalbreyeri Risaralda A27a
A hirsuta DarienChucuanti A29
A hirsuta DarienAltoTatabras A28c
A hirsuta DarienAltoTatabras A28a
A hirsuta ChocoCerroJanano A57b
A hirsuta ChocoCerroJanano A57c
A hirsuta ChocoCerroJanano A57a
A hirsuta intermedia RisaraldaPuebloRico A26b
A hirsuta intermedia RisaraldaPuebloRico A26a
A hirsuta intermedia RisaraldaPuebloRico A26e
A hirsuta intermedia RisaraldaPuebloRico A26d
A hirsuta intermedia RisaraldaPuebloRico A26c
A hirsuta intermedia ChocoSJPalmar A24a
A hirsuta intermedia ChocoSJPalmar A24c
A hirsuta intermedia ChocoSJPalmar A24e
A hirsuta intermedia ChocoSJPalmar A24d
A hirsuta intermedia ChocoSJPalmar A24b
14.688
trait value
4919.212
length=0.007
