# Supplementary material for: The Tracking of Moist Habitats Allowed Aiphanes (Arecaceae) to Cover the Elevation Gradient of the Northern Andes
Source: Front Plant Sci. 2022 Jun 27;13:881879. doi: 10.3389/fpls.2022.881879 (PMC9272002; doi:10.3389/fpls.2022.881879)

Supplementary Material

Supplementary Figure 6 - Niche volume projections on the two first principal components by the ecospat method of Broenniman et al., 2012 of same clade species pairs for all clades in the SSP phylogeny of Figure 1. Individual graphs portray the breadth of individual projections and of niche overlap between species.

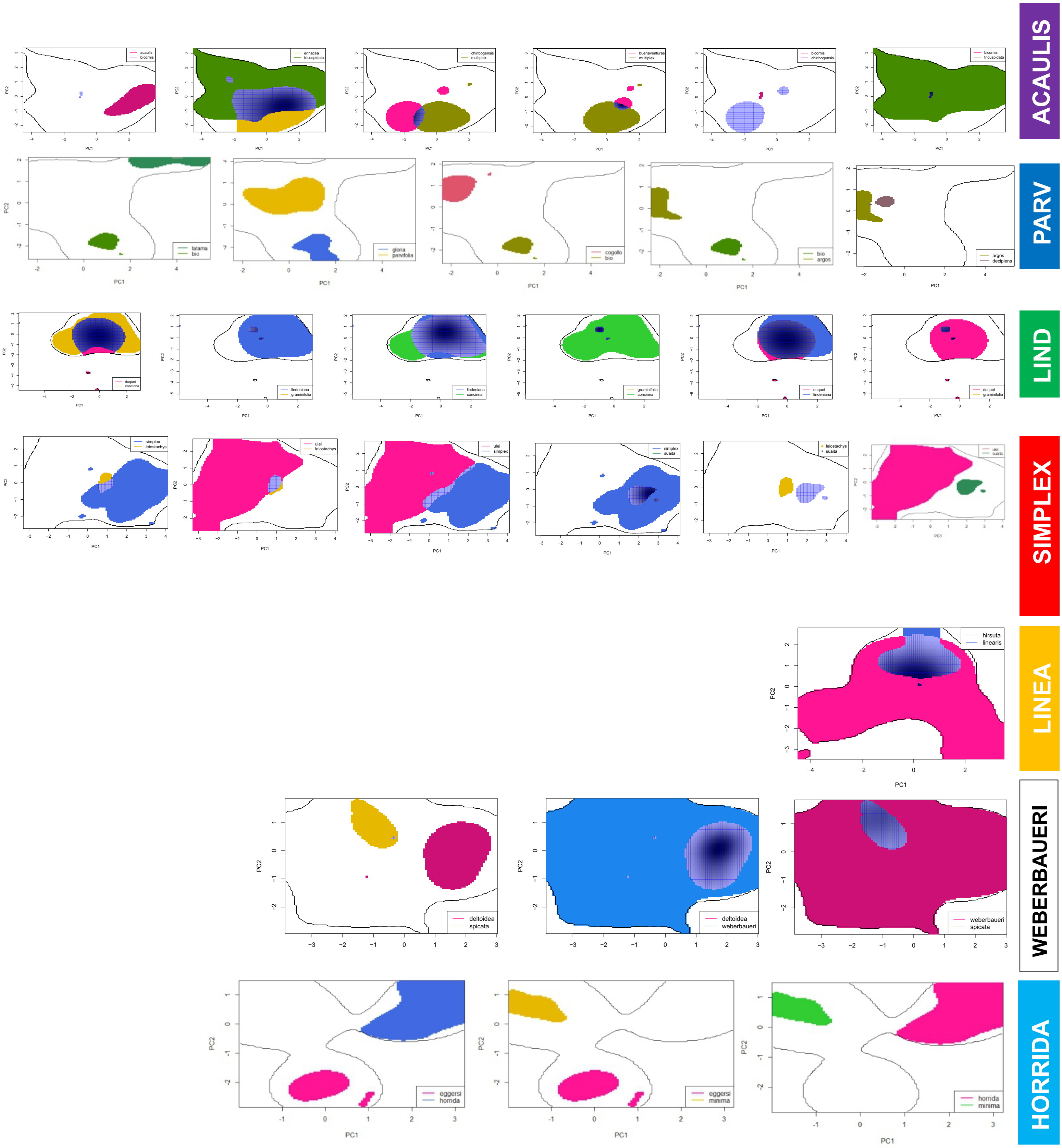

Supplement: Supplementary file 6 [file Data_Sheet_6.PDF]
