# Supplementary material for: The Tracking of Moist Habitats Allowed Aiphanes (Arecaceae) to Cover the Elevation Gradient of the Northern Andes
Source: Front Plant Sci. 2022 Jun 27;13:881879. doi: 10.3389/fpls.2022.881879 (PMC9272002; doi:10.3389/fpls.2022.881879)

Supplementary Material

Supplementary Figure 7– Principal component analysis of climatic variables for (**A**) temperature and (**B**) precipitation related variables (Bio1-11 and Bio12-19, respectively). Phylogenetic Generalized Least Squares lambda profiles for (**C**) temperature and (**D**) precipitation related Principal components 1 and 2 for the Sanger Sequence Phylogeny and (**E**) temperature and (**F**) precipitation related Principal components 1 and 2 for the Sequence Capture Phylogeny. (**G-J**) Plot diagnostics for the Phylogenetic Generalized Least Squares tests of phylogenetic signal of traits. Of four plots for each variable set, top left: residual density, top right: sample vs. theoretical quantiles, bottom left: residuals corrected by phylogeny vs the fitted value, bottom right: fitted vs. observed values for the variable set.

Principal component analyses of climatic variables

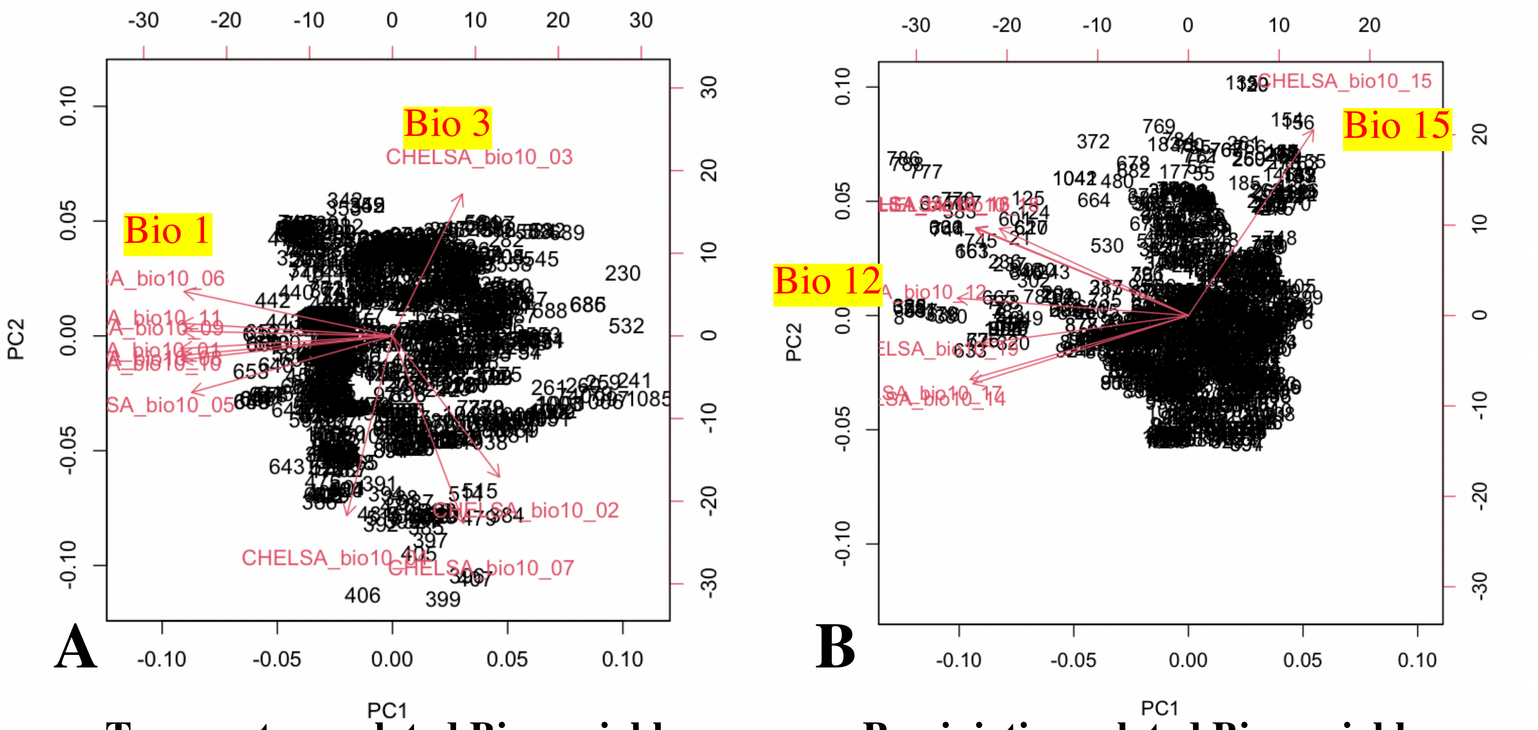

Temperature related Bio-variables **Precipiation related Bio-variables**

PGLS Lambda profiles (delta & kappa = 1)

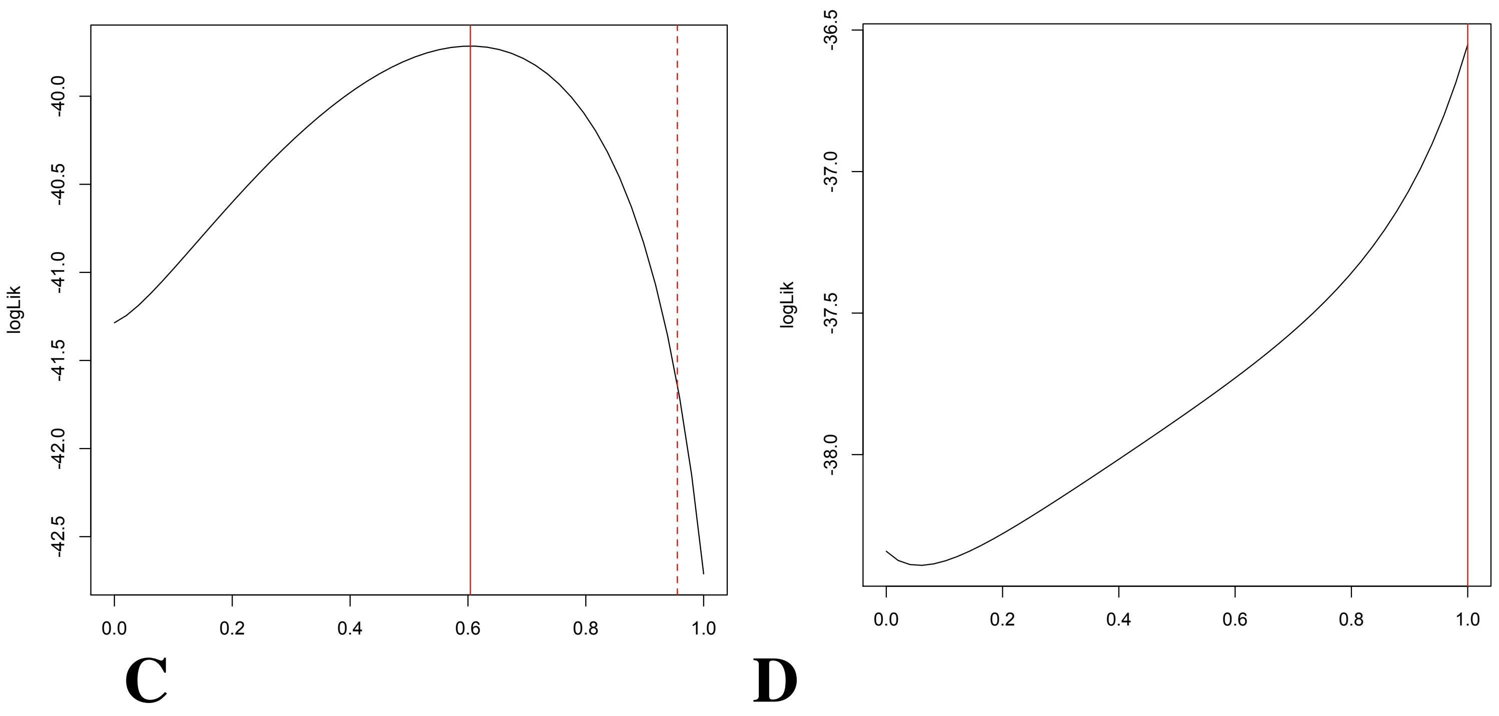

a. lambda temperature= 0.604 (lower bound= 0.07632

b. lambda precipitation= 1.00 (lower bound 0.000)

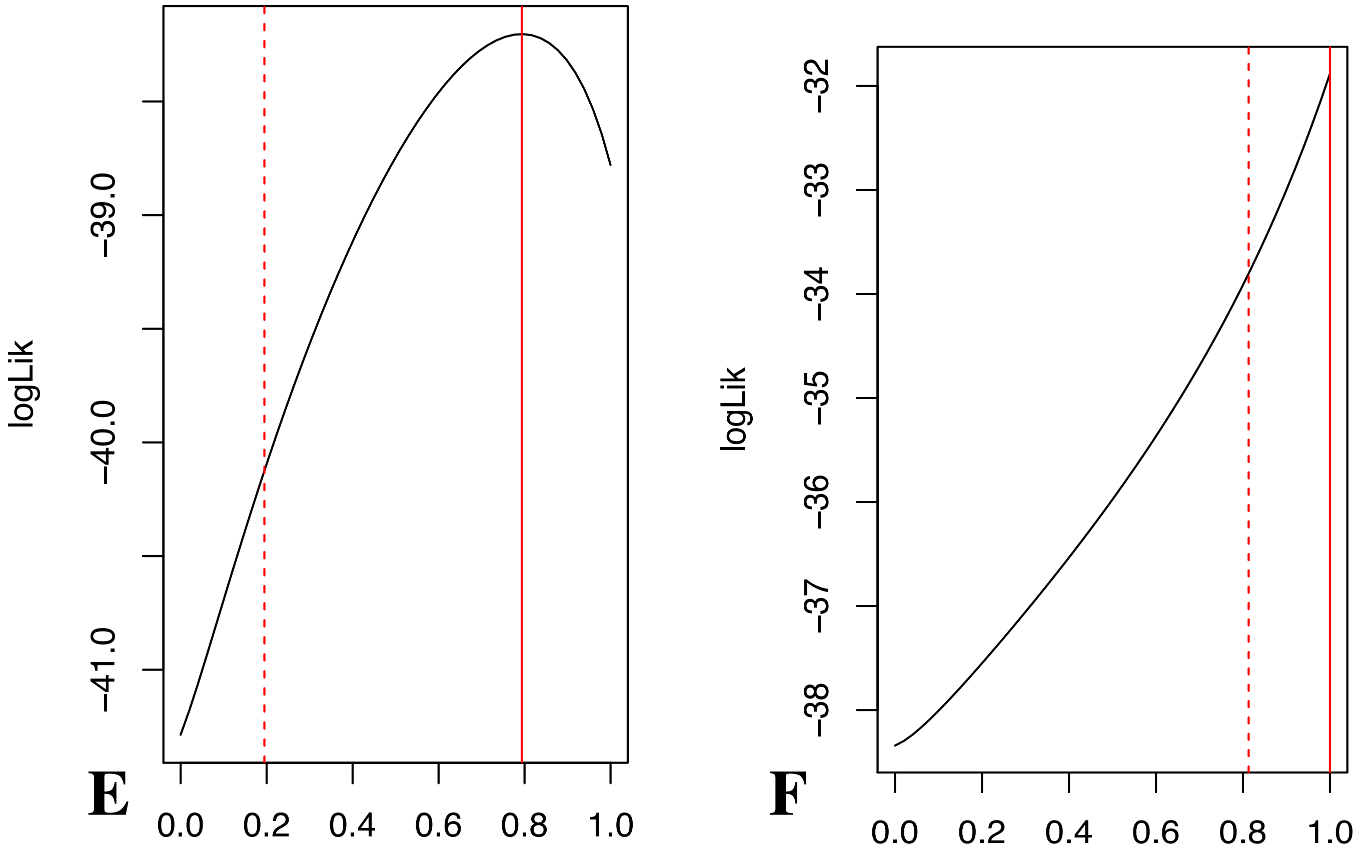

a. lambda temperature variables PC1 & PC2= 0.793 (lower bound= 0.195)

b. lambda precipitation variables PC1 & PC2= 1.00 (lower bound= 0.813)

Sanger Sequence Phylogeny

G Model diagnostics for Temperature variables

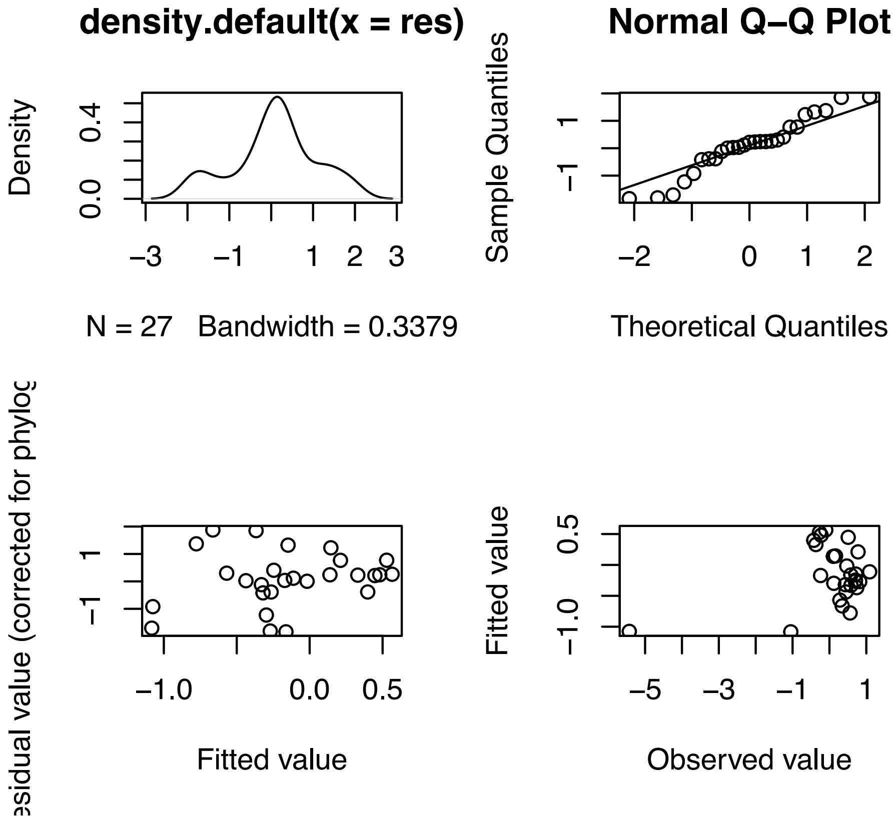

H Model diagnostics for Precipitation variables

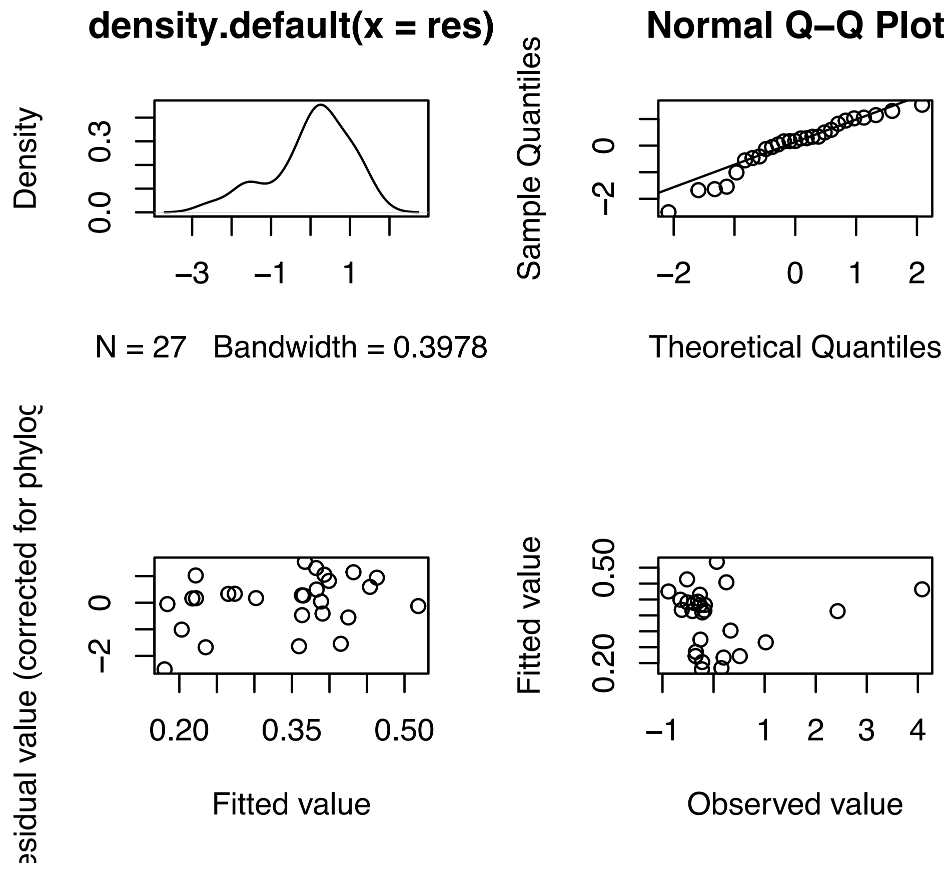

Sequence Capture Phylogeny

I Model diagnostics for Temperature variables

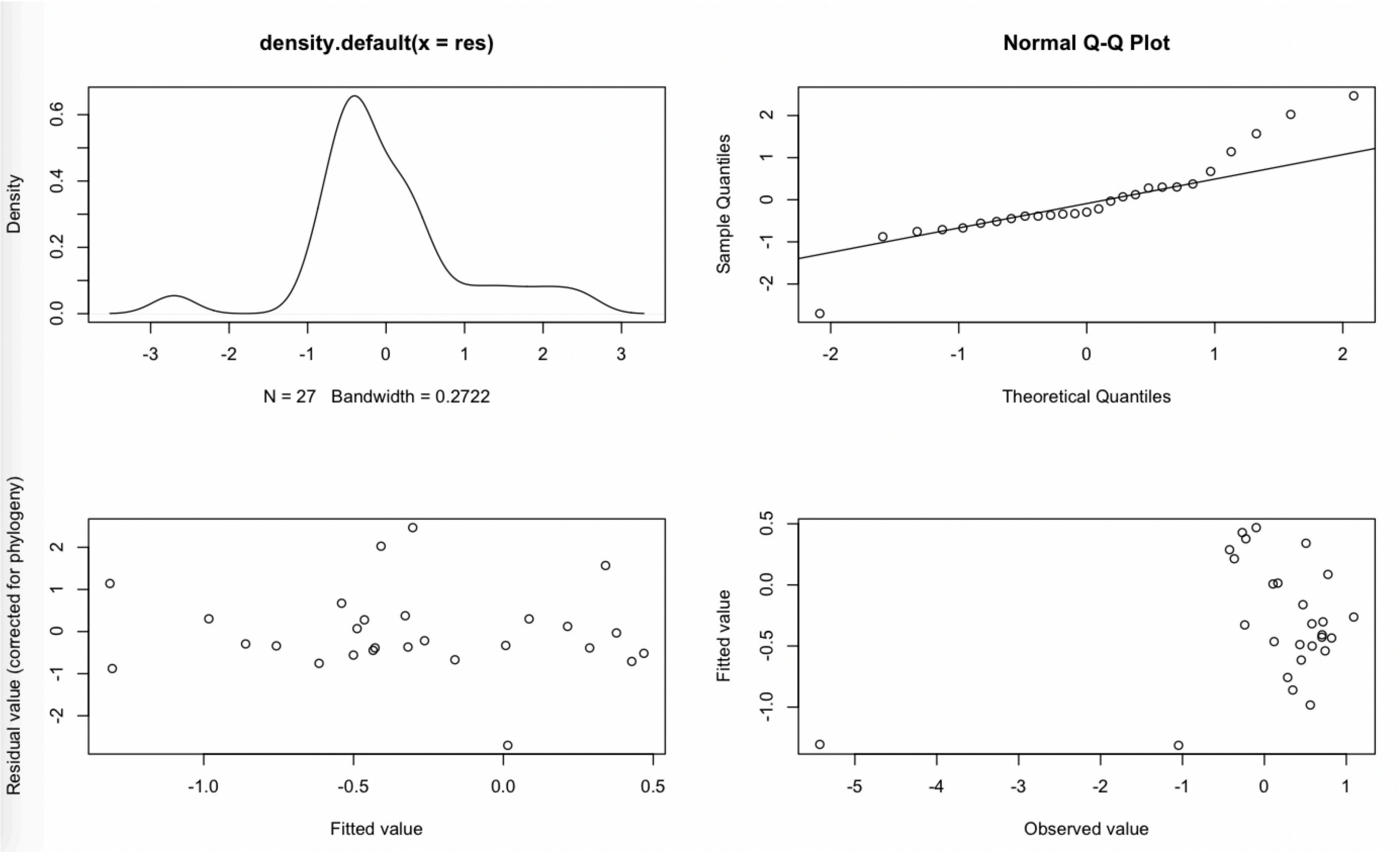

J Model diagnostics for Precipitation variables

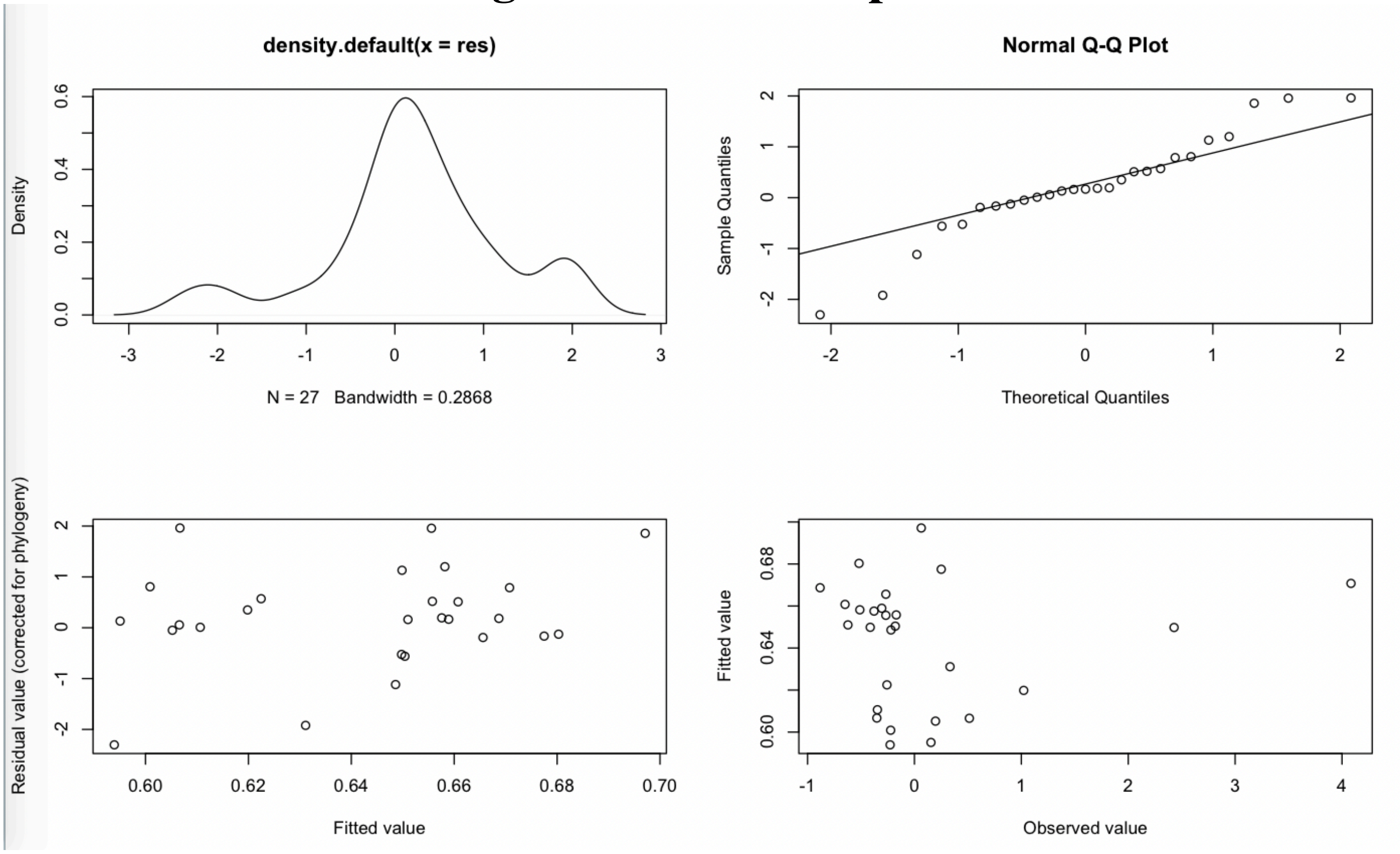

Supplement: Supplementary file 7 [file Data_Sheet_7.PDF]
