# Supplementary material for: The Tracking of Moist Habitats Allowed Aiphanes (Arecaceae) to Cover the Elevation Gradient of the Northern Andes
Source: Front Plant Sci. 2022 Jun 27;13:881879. doi: 10.3389/fpls.2022.881879 (PMC9272002; doi:10.3389/fpls.2022.881879)

Supplementary Material

Supplementary Figure 8 – Elevation ranges for all *Aiphanes* species, maximum elevation is represented by the green points and the minimum elevation by the blue points.

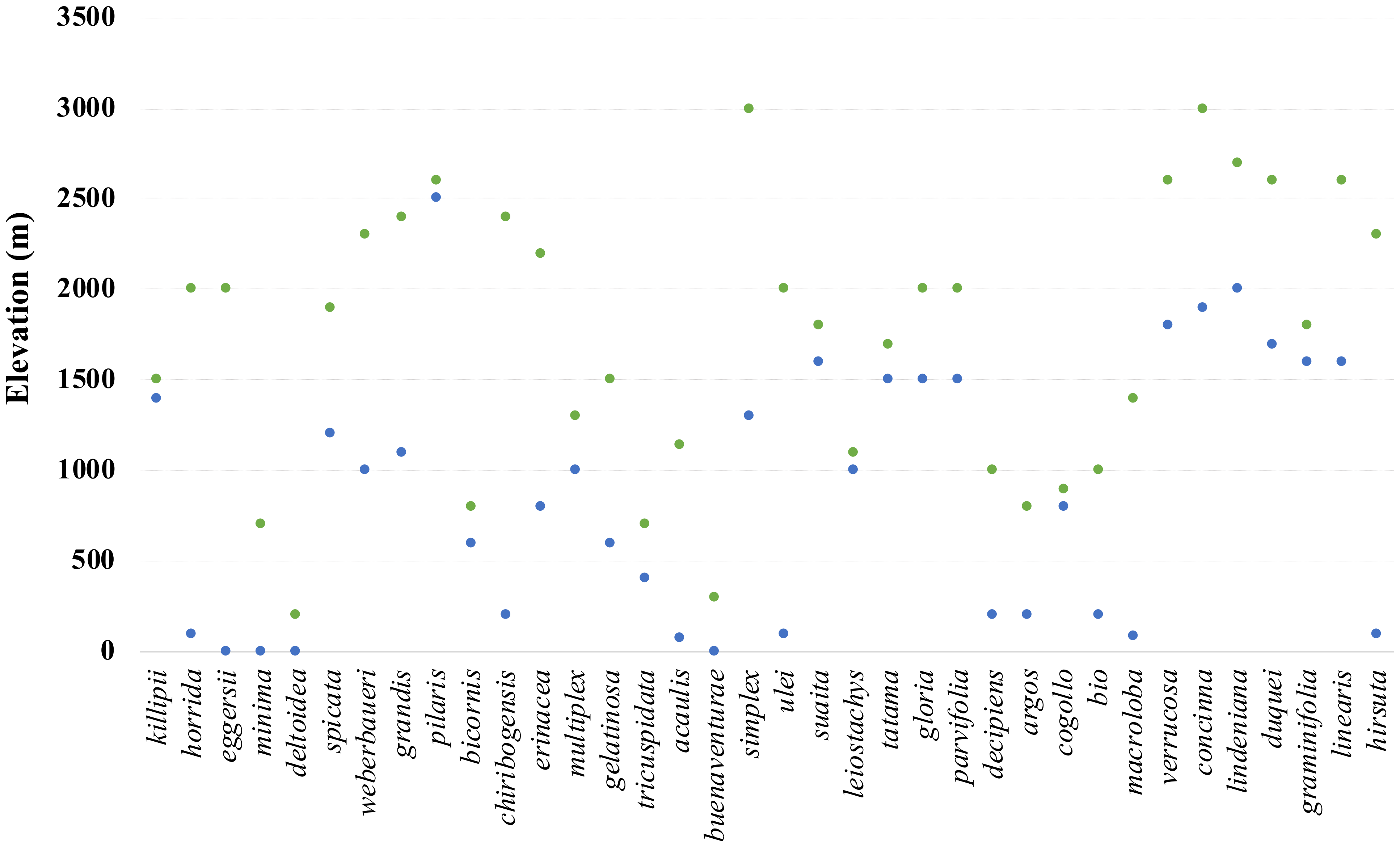

Supplement: Supplementary file 8 [file Data_Sheet_8.PDF]
