# Supplementary material for: The Tracking of Moist Habitats Allowed Aiphanes (Arecaceae) to Cover the Elevation Gradient of the Northern Andes
Source: Front Plant Sci. 2022 Jun 27;13:881879. doi: 10.3389/fpls.2022.881879 (PMC9272002; doi:10.3389/fpls.2022.881879)
Supplement: Supplementary file 9 [file Table_1.DOCX]

***Supplementary Material***

**Supplementary Table 1 -** Herbarium samples and Genbank accession codes for each region included in the Sanger sequencing phylogeny of the genus *Aiphanes*.

| **ID** | **Taxon** | **Country** | **Locality** | **Elevation (m)** | **Lat (°N)** | **Long (°W)** | **Collector & collection number** | **GenBank number (ITS)** | **GenBank number (prk)** | **GenBank number (rpb2)** |
| --- | --- | --- | --- | --- | --- | --- | --- | --- | --- | --- |
| AU985 | *Acrocomia aculeata* | United States of America | Montgomery Botanical Center, Miami-Dade County, Florida.  Cultivated plant. | 12 | 25.663 | -80.281 | W. J. Baker 1000 |  | MW447919 | MW447984 |
| AU986 | *Acrocomia crispa* | United States of America | Fairchild Tropical Garden, Miami-Dade County, Florida.  Cultivated plant. | 2 | 25.6787 | -80.274 | J. Roncal 79 |  | MW447920 | MW447985 |
| AU1710 | *Aiphanes acaulis* | Colombia | Biological Station Tutunendó, Chocó. | 78 | 5.7 | -76.5 | G. Galeano 7963 | MW405132 | MW447929 | MW447995 |
| RB5005 | *Aiphanes argos* | Colombia | Samaná Norte River, San Carlos, Antioquia. | 385 | 6.0557 | -74.8917 | R. Bernal 5005 | MW405121 | MW447914 | MW447980 |
| SEHG2932 | *Aiphanes argos* | Colombia | Right margin of the Samaná Norte River, village Palacio, San Luis, Antioquia. | 808 | 6.0031 | -74.9321 | S.E. Hoyos-Gómez 2932 |  | MW447918 | MW447983 |
| AU1371 | *Aiphanes bicornis* | Ecuador |  | 600 | 0.242500 | -78.9578 | J. West s.n. | MW405133 | MW447930 | MW447996 |
| RB4947 | *Aiphanes buenaventurae* | Colombia | Right margin of the Río Muerto, village El Brillante, Chocó. | 255 | 8.5881 | -77.4156 | R. Bernal 4947 | MW405120 | MW447913 | MW447979 |
| AU628 | *Aiphanes chiribogensis* | Ecuador | Las Palmeras on old Quito-Alluriquin. | 1940 | -0.2486 | -78.8049 | F. Borchsenius 652 | MW405134 | MW447931 | MW447997 |
| AU728 | *Aiphanes decipiens* | Colombia | Village La Josefina, San Luis, Antioquia. | 790 | 5.975702 | -74.9113 | J. Navarro 273 | MW405165 | MW447961 | MW448027 |
| AU1924 | *Aiphanes deltoidea* | Colombia | Biological Station El Zafire, Leticia, Amazonas. | 84 | -4.0056 | -69.8986 | G. Galeano 8528 | MW405135 | MW447932 | MW447998 |
| AU699 | *Aiphanes duquei* | Colombia | Cauca. | 1836 | 2.75 | -76.89 | R. Bernal 2852 | MW405136 | MW447933 | MW447999 |
| AU1243 | *Aiphanes eggersii* | Ecuador | Esmeraldas. | 255 | 0.688759 | -79.4846 | J.-C. Pintaud 1476 | MW405137 | MW447934 | MW448000 |
| AU918 | *Aiphanes eggersii* | United Kingdom | Richmond, Kew Palm House.  Cultivated plant. | 21 | 51.4789 | -0.29275 | cf. Baslev & Kristensen leg. 62012 (K) | MW405138 | MW447935 | MW448001 |
| AU627 | *Aiphanes erinacea* | Ecuador | Old road Alluriqin-Quito, km 10. | 1250 | -0.3056 | -78.8847 | F. Borchsenius 651 | MW405139 | MW447936 | MW448002 |
| AU630 | *Aiphanes erinacea* | Ecuador | Road Lita-San Lorenzo, km 17.8. | 830 | -0.8989 | -78.5436 | F. Borchsenius 657 | MW405140 | MW447937 | MW448003 |
| AU634 | *Aiphanes erinacea* | Ecuador | Road Baeza-Tena, km 32, Cordillera de Huacamayos. | 2200 | -0.6833 | -77.8667 | F. Borchsenius 626 | MW405141 | MW447938 |  |
| AU700 | *Aiphanes erinacea* | Colombia | El Tambo, PNN Munchique, village El Condor, Cauca. | 1800 | 2.7 | -76.9 | R. Bernal 2835 | MW405142 | MW447939 | MW448004 |
| RB4734 | *Aiphanes gloria* | Colombia | La Forzosa, Amalfi, Antioquia. | 1769 | 6.8729 | -75.1030 | R. Bernal 4734 |  | MW447912 | MW447978 |
| AU1896 | *Aiphanes gloria* | Colombia | Forest Reserve La Forzosa, quebrada La Soledad, Anorí, Antioquia. | 1624 | 7.0 | -75.1 | R. Bernal 4742 | MW405164 | MW447960 | MW448026 |
| AU1925 | *Aiphanes graminifolia* | Colombia | Quebrada Los Canelones, village San José de Suaita, Santander. | 1694 | 6.1750 | -73.4258 | R. Bernal 4825 | MW405145 |  | MW448007 |
| AU626 | *Aiphanes grandis* | Ecuador | Sambotambo, km 10.4 on road going N from road Piñas-Machala. | 1600 | -3.6033 | -79.7406 | F. Borchsenius 648 | MW405146 | MW447942 | MW448008 |
| AU1899 | *Aiphanes hirsuta* | Colombia | Village Manzanares, quebrada La Vivora, Amalfi, Antioquia | 1702 | 6.9 | -75.1 | R. Bernal 4752 | MW405147 | MW447943 | MW448009 |
| AU1900 | *Aiphanes hirsuta* | Colombia | Village Puerto Rico, quebrada La Chorrera, San Carlos, Antioquia. | 1560 | 6.2 | -75.0 | R. Bernal 4761 | MW405148 | MW447944 | MW448010 |
| AU1926 | *Aiphanes hirsuta* | Colombia | Village San Antonio, road going to Alto del Galápago, San José del Palmar, Chocó. | 1478 | 4.9 | -76.2 | R. Bernal 4785 | MW405149 | MW447945 | MW448011 |
| AU629 | *Aiphanes hirsuta* | Ecuador | Road Lita-San Lorenzo, km 16. | 860 | -0.8842 | -78.535 | F. Borchsenius 654 | MW405150 | MW447946 | MW448012 |
| AU703 | *Aiphanes hirsuta* | Colombia | El Barro, between Junin and Altaquer, Nariño. | 1100 | 1.3 | -78.1 | R. Bernal 2497 | MW405151 | MW447947 | MW448013 |
| RB5016 | *Aiphanes hirsuta intermedia* | Colombia | Pueblo Rico, Risaralda. | 1866 | 5.2145 | -76.0443 | R. Bernal 5016 |  | MW447916 | MW447982 |
| AU1230 | *Aiphanes horrida* | Peru | Lima. | 500 | -13.0176 | -76.2872 | F. Borchsenius. photo voucher | MW405152 | MW447948 | MW448014 |
| AU1242 | *Aiphanes horrida* | No Data | No Data | No Data | No Data | No Data | J.-C. Pintaud 1467 | MW405153 | MW447949 | MW448015 |
| AU1705 | *Aiphanes horrida* | Peru | Río Ucayali, 6 km N of Contamana,132 km NW of Pucallpa. Km 8 on Contamana - Aguas Calientes road, left side of road. | 174 | -7.2977 | -75.0078 | H. Balslev 7970 | MW405154 | MW447950 | MW448016 |
| AU1706 | *Aiphanes horrida* | Peru | Río Ucayali, 25 km SW of Orellana, 166 km NW of Pucallpa. Village of Inahuaya, App. 4 km from river. | 172 | -15.6306 | -75.2924 | H. Balslev 7992 | MW405155 | MW447951 | MW448017 |
| AU1711 | *Aiphanes horrida* | Bolivia | Cochabamba. | 653 | -16.91490 | -65.5072 | F. Borchsenius. photo voucher | MW405156 | MW447952 | MW448018 |
| AU698 | *Aiphanes horrida* | Colombia | Santander | No Data | No Data | No Data | R. Bernal 3434 | MZ581332 | MZ612414 | MZ612413 |
| AU704 | *Aiphanes killipi* | Colombia | San José de Suaita, Santander. | 1400 | 6.2 | -73.3 | R. Bernal 3433 | MW405157 | MW447953 | MW448019 |
| AU706 | *Aiphanes lindeniana* | Colombia | km 2-4 on road from El 18 (km 16 of Bucaramanga-Pamplona route) to Tona, Santander. | 1800 | 7.1 | -73.0 | R. Bernal 3484 | MW405159 | MW447955 | MW448021 |
| AU707 | *Aiphanes lindeniana* | Colombia | Route between cerro La Judía and San Ignacio, Tona, Santander. | 1900 | 7.1 | -73.1 | R. Bernal 3574 | MW405160 | MW447956 | MW448022 |
| AU705 | *Aiphanes lindeniana* | Colombia | km 2-4 on road from El 18 (km 16 of Bucaramanga-Pamplona route) to Tona, Santander. | 1800 | 7.1 | -73.0 | R. Bernal 3484 | MW405158 | MW447954 | MW448020 |
| AU1928 | *Aiphanes linearis* | Colombia | Nature Reserve Cerro El Inglés, village Boquerón, El Cairo, Valle del Cauca. | 2264 | 4.8 | -76.3 | R. Bernal 4772 | MW405161 | MW447957 | MW448023 |
| AU631 | *Aiphanes macroloba* | Ecuador | Road Lita-San Lorenzo, km 17.8. | 830 | 0.8989 | -78.5436 | F. Borchsenius 658 | MW405162 | MW447958 | MW448024 |
| AU984 | *Aiphanes minima* | United States of America | Fairchild Tropical Garden, Miami-Dade County, Florida.  Cultivated plant | 2 | 25.6787 | -80.274 | S. Zona 87 | MW405163 | MW447959 | MW448025 |
| AU702 | *Aiphanes multiplex* | Colombia | PNN Munchique, Vereda el Condor, El Tambo, Cauca. | 1800 | 2.7 | -76.9 | R. Bernal 2837 | MW405144 | MW447941 | MW448006 |
| RB5011a | *Aiphanes parvifolia* | Colombia | Route to Piedra Castrillón (SW from town), San Luis, Antioquia. | 1503 | 6.0 | -75.0 | R. Bernal 5011a | MW405122 | MW447915 | MW447981 |
| AU729 | *Aiphanes pilaris* | Colombia | nr. 9 km E of  San Francisco, on road from Sibundoy to  Mocoa, Putumayo. | 2580 | 7.1 | -76.9 | R. Bernal 2479 | MW405166 | MW447962 | MW448028 |
| AU1372 | *Aiphanes simplex* | Colombia | Forest Reserve El Romeral, 5 km SW of Angelópolis, Antioquia. | 2066 | 6.1 | 75.7 | R. Bernal 4767 | MW405167 | MW447963 | MW448029 |
| AU730 | *Aiphanes simplex* | Colombia | Road Florencia-Suaza, km 28, Florencia, Caquetá. | 1500 | 1.7 | -75.7 | R. Bernal 2892 | MW405169 | MW447965 | MW448031 |
| AU733 | *Aiphanes spicata* | Peru | Bosque de Protección Alto Mayo, Venceremos, San Martín. | 1620 | -5.886 | -77.575 | B. Millán 1223 (USM)/ J.-C. Pintaud 1361 | MW405170 | MW447966 | MW448032 |
| AU734 | *Aiphanes spicata* | Peru | Bosque de Protección Alto Mayo, San Martín. | 1620 | -5.886 | -77.575 | B. Millán 1221 (USM)/ J.-C. Pintaud 1357 | MW405171 | MW447967 | MW448033 |
| AU1929 | *Aiphanes suaita* | Colombia | Village San José de Suaita, La Meseta, quebrada La Lajita, Santander. | 1678 | 6.17 | -73.4 | R. Bernal 4815 | MW405168 | MW447964 | MW448030 |
| RB5018 | *Aiphanes tatama* | Colombia | La Selva, Risaralda. | 1587 | 5.2335 | -76.0719 | R. Bernal 5018 |  | MW447917 |  |
| AU1229 | *Aiphanes tricuspidata* | Colombia | No Data | No Data | No Data | No Data | R. Bernal s.n | MW405172 | MW447968 | MW448034 |
| AU701 | *Aiphanes ulei* | Colombia | El Barro, between Junin y Altaquer, Nariño. | 1100 | 1.3 | -78.1 | R. Bernal 2496 | MW405143 | MW447940 | MW448005 |
| AU1707 | *Aiphanes ulei* | Peru | Río Amazonas, 90 km NE of Iquitos. Village of Roca Eterna, approx. 4 km N of village. | 106 | -3.4387 | -72.5084 | H. Balslev 8121 | MW405173 | MW447969 | MW448035 |
| AU1709 | *Aiphanes ulei* | Ecuador | Tena. At South West base of Galeras Cordillera, on east side of Pusuno River. | 926 | -0.8842 | -77.5827 | T. Couvreur 125 | MW405174 |  | MW448036 |
| AU1930 | *Aiphanes ulei* | Colombia | Road Mocoa-Pitalito, 5 km of San Juan de Villalobos to Pitalito, Santa Rosa, Cauca. | 1435 | 1.449 | -76.434 | R. Bernal 4631 | MW405175 | MW447970 | MW448037 |
| AU624 | *Aiphanes ulei* | Ecuador | Road Plan de Milagro-Gualaquiza, km 2. | 1850 | -3.0167 | -78.5 | F. Borchsenius 633 | MW405176 |  | MW448038 |
| AU731 | *Aiphanes ulei* | Colombia | Road Florencia-Suaza, km 28, village Las Brisas, Florencia, Caquetá. | 1500 | 1.7 | -75.7 | R. Bernal 2891 | MW405177 | MW447971 | MW448039 |
| AU732 | *Aiphanes ulei* | Colombia | Guanganai river, Amazon river tributary., Leticia, Amazonas. | 150 | 3.9 | -70.15 | R. Bernal 2527 | MW405178 | MW447972 | MW448040 |
| AU625 | *Aiphanes verrucosa* | Ecuador | Road Yangana-Valladolid, km 30, Cruz del Soldado. | 2500 | -4.4983 | -79.1347 | F. Borchsenius 641 | MW405179 | MW447973 | MW448041 |
| AU1231 | *Aiphanes weberbaueri* | Peru | Between Moyobamba and Tarapoto. | 1157 | -6.317645 | -76.7061 | F. Borchsenius. photo voucher | MW405180 | MW447974 | MW448042 |
| AU1232 | *Aiphanes weberbaueri* | No Data | No Data | No Data | No Data | No Data | L. Santa-Cruz 1275 |  | MW447975 | MW448043 |
| AU1704 | *Aiphanes weberbaueri* | Peru | Just N of Río Toniromashi 6 km off Río Ucayali, 226 km S of Pucallpa. | 217 | -10.353 | -73.963 | H. Balslev 7648 | MW405181 | MW447976 | MW448044 |
| AU1708 | *Aiphanes weberbaueri* | Peru | Río Ucayali, eastern bank, 33km NW of Atalaya. | 244 | -10.593 | -73.889 | H. Balslev 7769 | MW405182 | MW447977 | MW448045 |
| AU890 | *Aiphanes weberbaueri* | Peru | Allpahuayo-Mishana National Reserve, Loreto. | 128 | -3.944 | -73.606 | B. Millán 1323 (USM)/ J.-C. Pintaud 1434 | MW405183 |  | MW448046 |
| AU982 | *Aiphanes weberbaueri* | Peru | No Data | No Data | No Data | No Data | B. Millán 1406 (USM)/J.-C. Pintaud 1460 | MW405184 |  | MW448047 |
| AU882 | *Astrocaryum chambira* | Peru | Allpahuayo-Mishana National Reserve, Loreto. | 128 | -3.944 | -73.606 | F. Kahn 2447 (USM) | MW405123 | MW447921 | MW447986 |
| AU884 | *Astrocaryum gynacanthum* | French Guiana | Grand Matoury | 232 | 4.86333 | -52.3561 | J.-C. Pintaud 340 | MW405124 | MW447922 | MW447987 |
| AU886 | *Astrocaryum mexicanum* | United States of America | Fairchild Tropical Garden, Miami-Dade County, Florida.  Cultivated plant | 2 | 25.6787 | -80.274 | J.-C. Pintaud 367 | MW405125 | MW447923 | MW447988 |
| AU899 | *Bactris acanthocarpa* | French Guiana | Route de Saint-Elié. | 30 | 5.3167 | -53.033 | J.-C. Pintaud 1447 | MW405126 |  | MW447989 |
| AU892 | *Bactris major* | Bolivia | Provincia Abel Iturralde. Road from San Buenaventura to Alto Madidi, km 70, comunidad Santa Rosa de Maravilla. | 300 | -13.9517 | -68.009 | H. Balslev 6737 | MW405127 | MW447924 | MW447990 |
| AU895 | *Bactris maraja* | Peru | Region Loreto, Distrito Maipuco. Río Urituyacu, caseRío Velasco ca. 40 km NE of outlet into Río Maronon. | 180 | -4.7486 | -75.14 | H. Balslev 6577 | MW405128 | MW447925 | MW447991 |
| AU908 | *Desmoncus mitis* | Peru | Provincia Loreto, Distrito Trompeteros, Río Corrientes at Nuevo Porvenir, ca. 10 km W of Trompeteros. | 141 | -3.8076 | -75.1462 | H. Balslev 6649 | MW405129 | MW447926 | MW447992 |
| AU894 | *Desmoncus orthacanthos* | Peru | Region Loreto, Distrito Maipuco. Río Urituyacu, caseRío Velasco ca. 40 km NE of outlet into Río Maronon. | 180 | -4.7486 | -75.14 | H. Balslev 6576 | MW405130 | MW447927 | MW447993 |
| AU905 | *Desmoncus polyacanthos* | Peru | Provincia Loreto, Distrito Trompeteros, Río Corrientes at Nuevo Porvenir, ca. 10 km W of Trompeteros. | 141 | -3.8076 | -75.1462 | H. Balslev 6620 | MW405131 | MW447928 | MW447994 |
