# Supplementary material for: The Tracking of Moist Habitats Allowed Aiphanes (Arecaceae) to Cover the Elevation Gradient of the Northern Andes
Source: Front Plant Sci. 2022 Jun 27;13:881879. doi: 10.3389/fpls.2022.881879 (PMC9272002; doi:10.3389/fpls.2022.881879)
Supplement: Supplementary file 10 [file Table_2.DOCX]

***Supplementary Material***

**Supplementary Table 2 -** List of samples used for sequence capture phylogenies of the genus *Aiphanes*, with Herbarium number and accession codes. Targeted sequencing sequence reads generated as part of this manuscript are available in NCBI (project PRJNA689999).

| **ID** | **Taxon** | **Country** | **Locality** | **Elevation (m)** | **Lat (°N)** | | **Long (°W)** | **Hearium code** | **Collector & collection number** |
| --- | --- | --- | --- | --- | --- | --- | --- | --- | --- |
| A48 | *Aiphanes acaulis* | Colombia | Michitá, Chocó. | 98 | 5.6927 | | -76.5975 | CBUCES-G 1004 | M. Sanín |
| A9 | *Aiphanes argos* | Colombia | Samaná Norte River, San Carlos, Antioquia. | 385 | 6.0557 | | -74.8917 | CBUCES-G | R. Bernal 5005 |
| A10a | *Aiphanes argos* | Colombia | Right margin of the Samaná Norte River, village Palacio, San Luis, Antioquia. | 808 | 6.0031 | | -74.9321 | CBUCES-G | SEHG 2932 |
| A47 | *Aiphanes buenaventurae* | Colombia | Unguía, Chocó. | 220 | 7.9812 | | -77.1325 | CBUCES-G | M. Sanín |
| A54 | *Aiphanes buenaventurae* | Colombia | Unguía, Chocó. | 220 | 7.9812 | | -77.1325 | CBUCES-G | M. Sanín |
| A16 | *Aiphanes concinna* | Colombia | Santa Rosa de Osos, Antioquia. | 2557 | 6.6658 | | -75.4841 | CBUCES-G | M. Sanín |
| A32a | *Aiphanes concinna* | Colombia | Isnos, Popayán. | 3110 | 2.1687 | | -76.3881 | CBUCES-G | M. Sanín |
| A32b | *Aiphanes concinna* | Colombia | Isnos, Popayán. | 3110 | 2.1687 | | -76.3881 | CBUCES-G | M. Sanín |
| A32c | *Aiphanes concinna* | Colombia | Isnos, Popayán. | 3110 | 2.1687 | | -76.3881 | CBUCES-G | M. Sanín |
| A32d | *Aiphanes concinna* | Colombia | Isnos, Popayán. | 3110 | 2.1687 | | -76.3881 | CBUCES-G | M. Sanín |
| A33a | *Aiphanes concinna* | Colombia | Suaza, Florencia. | 2151 | 1.7564 | | -75.7454 | CBUCES-G | M. Sanín |
| A33b | *Aiphanes concinna* | Colombia | Suaza, Florencia. | 2151 | 1.7564 | | -75.7454 | CBUCES-G | M. Sanín |
| A1a | *Aiphanes decipiens* | Colombia | La Josefina, San Luis, Antioquia. | 716 | 5.9824 | | -74.9141 | CBUCES-G | R. Bernal 4344 |
| A1b | *Aiphanes decipiens* | Colombia | La Josefina, San Luis, Antioquia. | 716 | 5.9824 | | -74.9141 | CBUCES-G | R. Bernal 4344 |
| A37 | *Aiphanes deltoidea* | Peru | Iquitos. | 114 | -3.4775 | | -72.8499 | CBUCES-G 487 | G. Galeano GG 8528 |
| A44 | *Aiphanes erinacea* | Colombia | Jardín Botánico del Quindío, Quindío. | 1511 | 4.5124 | | -75.6519 | CBUCES-G | M. Sanín |
| A50 | *Aiphanes gelatinosa* | Colombia | Ricaurte, Nariño. | 1119 | 1.2243 | | -78.0835 | CBUCES-G | M. Sanín |
| A5 | *Aiphanes gloria* | Colombia | La Forzosa, Amalfi, Antioquia. | 1769 | 6.8729 | | -75.103 | CBUCES-G 670 | R. Bernal 4734 |
| A6a | *Aiphanes gloria* | Colombia | La Forzosa, Amalfi, Antioquia. | 1769 | 6.8729 | | -75.103 | CBUCES-G 673 | R. Bernal 4740 |
| A6b | *Aiphanes gloria* | Colombia | La Forzosa, Amalfi, Antioquia. | 1769 | 6.8729 | | -75.103 | CBUCES-G | M. Sanín |
| A18 | *Aiphanes hirsuta* | Colombia | Amalfi, Antioquia. | 1769 | 6.8729 | | -75.103 | CBUCES-G 665 | R. Bernal 4722 |
| A21a | *Aiphanes hirsuta* | Colombia | San Carlos, Antioquia. | 1562 | 6.1953 | | -75.0431 | CBUCES-G 371 | R. Bernal 4761 |
| A21b | *Aiphanes hirsuta* | Colombia | San Carlos, Antioquia. | 1562 | 6.1953 | | -75.0431 | CBUCES-G 372 | R. Bernal 4761 |
| A21c | *Aiphanes hirsuta* | Colombia | San Carlos, Antioquia. | 1562 | 6.1953 | | -75.0431 | CBUCES-G 373 | R. Bernal 4761 |
| A25 | *Aiphanes hirsuta* | Colombia | Suaita, Santander. | 1676 | 6.17516 | | -73.4261 | CBUCES-G 711 | R. Bernal 4817 |
| A28a | *Aiphanes hirsuta* | Colombia | Unguía, Chocó. | 1069 | 8.2327 | | -77.2319 | CBUCES-G | R. Bernal 4973 |
| A28c | *Aiphanes hirsuta* | Colombia | Unguía, Chocó. | 1069 | 8.2327 | | -77.2319 | CBUCES-G | R. Bernal 4973 |
| A29 | *Aiphanes hirsuta* | Panama | Chucuanti, Darién. | 1069 | 8.1827 | | -77.7304 | CBUCES-G | ACS 471 |
| A57a | *Aiphanes hirsuta* | Colombia | Cabo Corriente, Chocó. | 259 | 5.4989 | | -77.5030 | CBUCES-G | M. Sanín |
| A57b | *Aiphanes hirsuta* | Colombia | Cabo Corriente, Chocó. | 259 | 5.4989 | | -77.5030 | CBUCES-G | M. Sanín |
| A57c | *Aiphanes hirsuta* | Colombia | Cabo Corriente, Chocó. | 259 | 5.4989 | | -77.5030 | CBUCES-G | M. Sanín |
| A11a | *Aiphanes hirsuta fosteri* | Colombia | Jardín Botánico del Quindío, Quindío. | 1511 | 4.5124 | | -75.6519 | CBUCES-G | M. Sanín |
| A11b | *Aiphanes hirsuta fosteri* | Colombia | Jardín Botánico del Quindío, Quindío. | 1511 | 4.5124 | | -75.6519 | CBUCES-G | M. Sanín |
| A19 | *Aiphanes hirsuta fosteri* | Colombia | Amalfi, Antioquia. | 1627 | 7.0010 | | -75.1247 | CBUCES-G 679 | R. Bernal 4747 |
| A24a | *Aiphanes hirsuta intermedia* | Colombia | San José del Palmar, Chocó. | 1666 | 4.8512 | | -76.2180 | CBUCES-G 377 | R. Bernal 4785 |
| A24b | *Aiphanes hirsuta intermedia* | Colombia | San José del Palmar, Chocó. | 1666 | 4.8512 | | -76.2180 | CBUCES-G 378 | R. Bernal 4785 |
| A24c | *Aiphanes hirsuta intermedia* | Colombia | San José del Palmar, Chocó. | 1666 | 4.8512 | | -76.2180 | CBUCES-G 379 | R. Bernal 4785 |
| A24d | *Aiphanes hirsuta intermedia* | Colombia | San José del Palmar, Chocó. | 1666 | 4.8512 | | -76.2180 | CBUCES-G 380 | R. Bernal 4785 |
| A24e | *Aiphanes hirsuta intermedia* | Colombia | San José del Palmar, Chocó. | 1666 | 4.8512 | | -76.2180 | CBUCES-G 382 | R. Bernal 4785 |
| A26a | *Aiphanes hirsuta intermedia* | Colombia | Pueblo Rico, Risaralda. | 1866 | 5.2145 | | -76.0443 | CBUCES-G | R. Bernal 5016 |
| A26b | *Aiphanes hirsuta intermedia* | Colombia | Pueblo Rico, Risaralda. | 1866 | 5.2145 | | -76.0443 | CBUCES-G | R. Bernal 5016 |
| A26c | *Aiphanes hirsuta intermedia* | Colombia | Pueblo Rico, Risaralda. | 1866 | 5.2145 | | -76.0443 | CBUCES-G | R. Bernal 5016 |
| A26d | *Aiphanes hirsuta intermedia* | Colombia | Pueblo Rico, Risaralda. | 1866 | 5.2145 | | -76.0443 | CBUCES-G | R. Bernal 5016 |
| A26e | *Aiphanes hirsuta intermedia* | Colombia | Pueblo Rico, Risaralda. | 1866 | 5.2145 | | -76.0443 | CBUCES-G | R. Bernal 5016 |
| A20b | *Aiphanes hirsuta kalbreyeri* | Colombia | Amalfi, Antioquia. | 1746 | 6.8861 | | -75.1183 | CBUCES-G 682 | R. Bernal 4752 |
| A27a | *Aiphanes hirsuta kalbreyeri* | Colombia | La Selva, Risaralda. | 1526 | 5.2335 | | -76.0719 | CBUCES-G | R. Bernal 5020 |
| A27b | *Aiphanes hirsuta kalbreyeri* | Colombia | La Selva, Risaralda. | 1526 | 5.2335 | | -76.0719 | CBUCES-G | R. Bernal 5020 |
| A27c | *Aiphanes hirsuta kalbreyeri* | Colombia | La Selva, Risaralda. | 1526 | 5.2335 | | -76.0719 | CBUCES-G | R. Bernal 5020 |
| A27d | *Aiphanes hirsuta kalbreyeri* | Colombia | La Selva, Risaralda. | 1526 | 5.2335 | | -76.0719 | CBUCES-G | R. Bernal 5020 |
| A27e | *Aiphanes hirsuta kalbreyeri* | Colombia | La Selva, Risaralda. | 1526 | 5.2335 | | -76.0719 | CBUCES-G | R. Bernal 5020 |
| A53 | *Aiphanes hirsuta kalbreyeri* | Colombia | Angelópolis, Antioquia. | 1910 | 6.1121 | | -75.7055 | CBUCES-G | M. Sanín |
| A56 | *Aiphanes horrida* | Colombia | Jardín Botánico del Quindío, Quindío. | 1511 | 4.5124 | | -75.6519 | CBUCES-G | M. Sanín |
| A60a | *Aiphanes horrida* | Colombia | Ituango, Antioquia. | 237 | 7.1403 | | -75.6504 | CBUCES-G | V. Correa |
| A60b | *Aiphanes horrida* | Colombia | Ituango, Antioquia. | 237 | 7.1403 | | -75.6504 | CBUCES-G | V. Correa |
| A49a | *Aiphanes killipii* | Colombia | Suaita, Santander. | 1424 | 6.1636 | | -73.4519 | CBUCES-G | M. Sanín |
| A49b | *Aiphanes killipii* | Colombia | Suaita, Santander. | 1424 | 6.1636 | | -73.4519 | CBUCES-G | M. Sanín |
| A42 | *Aiphanes leiostachys* | Colombia | San Carlos, Antioquia. | 1124 | 6.1912 | | -75.0181 | CBUCES-G | M. Sanín |
| A51 | *Aiphanes leiostachys* | Colombia | San Carlos, Antioquia. | 1124 | 6.0449 | | -74.9991 | CBUCES-G | M. Sanín |
| A35a | *Aiphanes lindeniana* | Colombia | San Luis de Toledo, Cundinamarca. | 1868 | 4.4901 | | -73.661 | CBUCES-G | M. Sanín |
| A35b | *Aiphanes lindeniana* | Colombia | San Luis de Toledo, Cundinamarca. | 1868 | 4.4901 | | -73.661 | CBUCES-G | M. Sanín |
| A35d | *Aiphanes lindeniana* | Colombia | San Luis de Toledo, Cundinamarca. | 1868 | 4.4901 | | -73.661 | CBUCES-G | M. Sanín |
| A35e | *Aiphanes lindeniana* | Colombia | San Luis de Toledo, Cundinamarca. | 1868 | 4.4901 | | -73.661 | CBUCES-G | M. Sanín |
| A36a | *Aiphanes lindeniana* | Colombia | San Juanito, Cundinamarca. | 1919 | 4.4546 | | -73.6756 | CBUCES-G | M. Sanín |
| A36b | *Aiphanes lindeniana* | Colombia | San Juanito, Cundinamarca. | 1919 | 4.4546 | | -73.6756 | CBUCES-G | M. Sanín |
| A36d | *Aiphanes lindeniana* | Colombia | San Juanito, Cundinamarca. | 1919 | 4.4546 | | -73.6756 | CBUCES-G | M. Sanín |
| A36e | *Aiphanes lindeniana* | Colombia | San Juanito, Cundinamarca. | 1919 | 4.4546 | | -73.6756 | CBUCES-G | M. Sanín |
| A45 | *Aiphanes lindeniana* | Colombia | Piedecuesta, Santander. | 2476 | 7.06761 | | -72.9899 | CBUCES-G | M. Sanín |
| A22a | *Aiphanes linearis* | Colombia | El Cairo, Valle del Cauca. | 2227 | 4.7578 | | -76.2835 | CBUCES-G 465 | R. Bernal 4768 |
| A22b | *Aiphanes linearis* | Colombia | El Cairo, Valle del Cauca. | 2227 | 4.7578 | | -76.2835 | CBUCES-G 466 | R. Bernal 4768 |
| A8a | *Aiphanes linearis* | Colombia | Alto Caldera, Granada, Antioquia. | 2091 | 6.1833 | | -75.1166 | CBUCES-G | SEHG 3264 |
| A8b | *Aiphanes linearis* | Colombia | Alto Caldera, Granada, Antioquia. | 2091 | 6.1833 | | -75.1166 | CBUCES-G | SEHG 3265 |
| A8c | *Aiphanes linearis* | Colombia | Alto Caldera, Granada, Antioquia. | 2091 | 6.1833 | | -75.1166 | CBUCES-G | SEHG 3266 |
| A15b | *Aiphanes linearis* | Colombia | Envigado, Antioquia. | 2287 | 6.1232 | | -75.5873 | CBUCES-G 998 | M. Sanín |
| A15c | *Aiphanes linearis* | Colombia | Envigado, Antioquia. | 2287 | 6.1232 | | -75.5873 | CBUCES-G 999 | M. Sanín |
| A17a | *Aiphanes linearis* | Colombia | Alto San Miguel, Caldas, Antioquia. | 1929 | 6.0394 | | -75.6203 | CBUCES-G 1000 | M. Sanín |
| A17b | *Aiphanes linearis* | Colombia | Alto San Miguel, Caldas, Antioquia. | 1929 | 6.0394 | | -75.6203 | CBUCES-G 1001 | M. Sanín |
| A17c | *Aiphanes linearis* | Colombia | Alto San Miguel, Caldas, Antioquia. | 1929 | 6.0394 | | -75.6203 | CBUCES-G 1002 | M. Sanín |
| A23a | *Aiphanes linearis* | Colombia | El Cairo, Valle del Cauca. | 2227 | 4.7578 | | -76.2835 | CBUCES-G 693 | R. Bernal 4772 |
| A23b | *Aiphanes linearis* | Colombia | El Cairo, Valle del Cauca. | 2227 | 4.7578 | | -76.2835 | CBUCES-G 694 | R. Bernal 4772 |
| A38 | *Aiphanes macroloba* | Colombia | Bahía Solano, Chocó. | 13 | 6.1173 | | -77.3665 | CBUCES-G 539 | JCC 21 |
| A58a | *Aiphanes macroloba* | Colombia | Janano, Chocó. | 619 | 5.5697 | | -77.2162 | CBUCES-G | M. Sanín |
| A58b | *Aiphanes macroloba* | Colombia | Janano, Chocó. | 619 | 5.5697 | | -77.2162 | CBUCES-G | M. Sanín |
| A4a | *Aiphanes parvifolia* | Colombia | Piedra Castrillón, San Luis, Antioquia. | 1046 | 6.0505 | | -74.9994 | CBUCES-G | M. Sanín |
| A4b | *Aiphanes parvifolia* | Colombia | Piedra Castrillón, San Luis, Antioquia. | 1046 | 6.0505 | | -74.9994 | CBUCES-G | M. Sanín |
| A39 | *Aiphanes pilaris* | Colombia | San Francisco, Mocoa, Putumayo. | 2160 | 1.1772 | | -76.8824 | CBUCES-G | M. Sanín |
| A40a | *Aiphanes simplex* | Colombia | Jardín Botánico del Quindío, Quindío. | 1511 | 4.5124 | | -75.6519 | CBUCES-G | M. Sanín |
| A40b | *Aiphanes simplex* | Colombia | Jardín Botánico del Quindío, Quindío. | 1511 | 4.5124 | | -75.6519 | CBUCES-G | M. Sanín |
| A40c | *Aiphanes simplex* | Colombia | Jardín Botánico del Quindío, Quindío. | 1511 | 4.5124 | | -75.6519 | CBUCES-G | M. Sanín |
| A52a | *Aiphanes simplex* | Colombia | Boquía, Quindío. | 1968 | 4.6553 | | -75.5953 | CBUCES-G | M. Sanín |
| A52b | *Aiphanes simplex* | Colombia | Boquía, Quindío. | 1968 | 4.6553 | | -75.5953 | CBUCES-G | M. Sanín |
| A41a | *Aiphanes suaita* | Colombia | Suaita, Santander. | 1597 | 6.1014 | | -73.4456 | CBUCES-G | M. Sanín |
| A41b | *Aiphanes suaita* | Colombia | Suaita, Santander. | 1597 | 6.1014 | | -73.4456 | CBUCES-G | M. Sanín |
| A41c | *Aiphanes suaita* | Colombia | Suaita, Santander. | 1597 | 6.1014 | | -73.4456 | CBUCES-G | M. Sanín |
| A7a | *Aiphanes tatama* | Colombia | La Selva, Risaralda. | 1587 | 5.2335 | | -76.0719 | CBUCES-G | R. Bernal 5018 |
| A43a | *Aiphanes tricuspidata* | Colombia | Jardín Botánico del Quindío, Quindío. | 1511 | 4.5124 | | -75.6519 | CBUCES-G | M. Sanín |
| A43b | *Aiphanes tricuspidata* | Colombia | Jardín Botánico del Quindío, Quindío. | 1511 | 4.5124 | | -75.6519 | CBUCES-G | M. Sanín |
| A46a | *Aiphanes ulei* | Colombia | Road Pitalito-Mocoa, Putumayo. | 736 | 1.0792 | | -76.6671 | CBUCES-G | M. Sanín |
| A46b | *Aiphanes ulei* | Colombia | Road Pitalito-Mocoa, Putumayo. | 736 | 1.0792 | | -76.6671 | CBUCES-G | M. Sanín |
| 1177 | *Asterogyne guianensis* | French Guiana | No data | No data | No data | | No data | No data | No data |
| G13c | *Geonoma undata* | Colombia | San Luis de Toledo, Cundinamarca. | 1868 | 4.4901 | | -73.661 | CBUCES-G | M. Sanín |
| 2477 | *Calyptrogyne costatifrons* | Colombia | Acandí, Chocó. | 218 | 8.576 | | -77.397 | CBUCES-G 732 | R. Bernal 4930 |
| 984 | *Cocos nucifera* | No data | No data | No data | No data | | No data | No data | No data |
| B743 | *Bactris gasipaes* | No data | No data | No data | No data | | No data | No data | No data |
| coco2 | *Cocos nucifera* | Colombia | No data | No data | No data | | No data | No data | T. Arias |
| coco2 | *Cocos nucifera* | Colombia | No data | No data | No data | | No data | No data | T. Arias |
| TC117 | *Geonoma macrostachys* | Ecuador | No data | No data | No data | | No data | No data | No data |
| AC72 | *Welfia regia* | Panamá | No data | No data | No data | | No data | No data | No data |
| AC64 | *Pholidostachys dactyloides* | Panamá | No data | No data | No data | | No data | No data | No data |
| ch2 | *Chamaedorea alleni* | Colombia | Istmina, Chocó. | 147 | 5.15915 | -76.69153 | | CBUCES-G | J.C. Copete |
| ch3 | *Chamaedorea alleni* | Colombia | Istmina, Chocó. | 147 | 5.15915 | -76.69153 | | CBUCES-G | J.C. Copete |
| 3125 | *Socratea exorrhiza* | No data | No data | No data | No data | | No data | No data | No data |
| 296B | *Wettinia maynensis* | No data | No data | No data | No data | | No data | No data | No data |
| 1011 | *Licuala merguensis* | No data | No data | No data | No data | | No data | No data | No data |
| 523B | *Ceroxylon alpinum* | No data | No data | No data | No data | | No data | No data | No data |
| 8a | *Ceroxylon ceriferum* | Venezuela | Colonia Tovar, Aragua. | 2220 | 10.424 | | -67.291 | CBUCES-G | MJS |
| out1 | *Ravenea sambiranensis* | Madagascar | Private collection | No data | No data | | No data | CBUCES-G | NYBG |
| out7 | *Phytelephas aequatorialis* | Colombia | Private collection | 1511 | 4.5124 | | -75.6519 | CBUCES-G | JBQ |
| out11 | *Ammandra decasperma* | Colombia | Chocó | 140 | 5.74638 | | -77.22748 | CBUCES-G | M. Sanín |
| out6 | *Phytelephas tumacana* | Colombia | Private collection | 1511 | 4.5124 | | -75.6519 | CBUCES-G | JBQ |
| out5 | *Phytelephas macrocarpa* | Colombia | No data | No data | No data | | No data | CBUCES-G |  |
| out8 | *Phytelephas* sp. | Colombia | San Agustín, Huila. | 1340 | 1.8633 | | -76.2311 | CBUCES-G | M. Sanín |
| 1017 | *Licuala distans* | No data | No data | No data | No data | | No data | No data | No data |
