# Supplementary material for: The Tracking of Moist Habitats Allowed Aiphanes (Arecaceae) to Cover the Elevation Gradient of the Northern Andes
Source: Front Plant Sci. 2022 Jun 27;13:881879. doi: 10.3389/fpls.2022.881879 (PMC9272002; doi:10.3389/fpls.2022.881879)
Supplement: Supplementary file 11 [file Table_3.DOCX]

***Supplementary Material***

**Supplementary Table 3.** Variance Inflation Factor (VIF) for seven non-collinear (VIF < 10) CHELSEA bioclimatic variables.

| **Variables** | **VIF** |
| --- | --- |
| Bio2: Mean Diurnal Range | 2.65 |
| Bio3: Isothermality | 3.22 |
| Bio4: Temperature Seasonality | 3.87 |
| Bio8: Mean Temperature of Wettest Quarter | 1.61 |
| Bio15: Precipitation Seasonality | 1.82 |
| Bio18: Precipitation of Warmest Quarter | 1.48 |
| Bio19: Precipitation of Coldest Quarter | 2.14 |
