# Supplementary material for: The Tracking of Moist Habitats Allowed Aiphanes (Arecaceae) to Cover the Elevation Gradient of the Northern Andes
Source: Front Plant Sci. 2022 Jun 27;13:881879. doi: 10.3389/fpls.2022.881879 (PMC9272002; doi:10.3389/fpls.2022.881879)
Supplement: Supplementary file 13 [file Table_5.DOCX]

***Supplementary Material***

**Supplementary Table 5.** Pairwise comparisons of niche overlap (D). p-values of the niche equivalency and similarity tests are given for a pairwise comparison.

| **Clade** | **Pairs species** | **Schoener’s D Index** | ***p-value equivalency*** | ***p-value similarity*** |
| --- | --- | --- | --- | --- |
| acaulis | *acaulis vs buenaventurae* | 0.00 | 1 | 0.17822 |
|  | *acaulis vs bicornis* | 0.00 | 1 | 1 |
|  | *acaulis vs chiribogensis* | 0.00 | 1 | 1 |
|  | *acaulis vs erinacea* | 0.19 | 0.91089 | 0.19802 |
|  | *acaulis vs multiplex* | 0.07 | 0.9802 | 0.41584 |
|  | *acaulis vs tricuspidata* | 0.06 | 1 | 0.22772 |
|  | *acaulis vs gelatinosa* | **0.3084** | 0.91089 | 0.17822 |
|  | *buenaventurae vs bicornis* | 0.00 | 1 | 1 |
|  | *buenaventurae vs chiribogensis* | 0.00 | 1 | 1 |
|  | *buenaventurae vs erinacea* | 0.0568 | 0.9901 | 0.1386 |
|  | *buenaventurae vs multiplex* | 0.0179 | 1 | 0.4158 |
|  | *buenaventurae vs tricuspidata* | 0.0095 | 1 | 0.4554 |
|  | *buenaventurae vs gelatinosa* | **0.2305** | 0.9109 | 0.0396 |
|  | *bicornis vs chiribogensis* | 0.0000 | 1 | 1.0000 |
|  | *bicornis vs erinacea* | 0.0026 | 1 | 0.3564 |
|  | *bicornis vs multiplex* | 0.0000 | 1 | 1.0000 |
|  | *bicornis vs tricuspidata* | 0.0013 | 1 | 0.5842 |
|  | *bicornis vs gelatinosa* | 0.0000 | 1 | 1.0000 |
|  | *chiribogensis vs erinacea* | 0.1308 | 1 | 0.495 |
|  | *chiribogensis vs multiplex* | 0.0713 | 1 | 0.2772 |
|  | *chiribogensis vs tricuspidata* | 0.0358 | 1 | 0.7723 |
|  | *chiribogensis vs gelatinosa* | 0.0016 | 1 | 0.5941 |
|  | *multiplex vs tricuspidata* | 0.0159 | 1 | 0.6534 |
|  | *multiplex vs gelatinosa* | **0.2104** | 0.9802 | 0.2376 |
|  | *tricuspidata vs gelatinosa* | 0.0541 | 1 | 0.5743 |
|  | *erinacea vs tricuspidata* | 0.1260 | 0.9901 | 0.6436 |
|  | *erinacea vs gelatinosa* | **0.2605** | 0.8317 | 0.1485 |
|  | *erinacea vs multiplex* | **0.5246** | 0.5149 | 0.0099 |
| horrida | eggersi vs horrida | 0 | 1 | 1 |
|  | eggersi vs minima | 0 | 1 | 1 |
|  | horrida vs minima | 0 | 1 | 1 |
| hirsuta | hirsuta vs linearis | 0.1242 | 0.9901 | 0.7327 |
| simplex | ulei vs simplex | 0.0185 | 1 | 0.7525 |
|  | ulei vs leiostachys | 0.0091 | 1 | 0.6832 |
|  | ulei vs suaita | 0 | 1 | 1 |
|  | simplex vs leiostachys | 0.0136 | 1 | 0.3762 |
|  | simplex vs suaita | 0.1477 | 0.8812 | 0.1386 |
|  | leiostachys vs suaita | 0 | 1 | 1 |
| deltoidea | deltoidea vs weberbaueri | 0.1097 | 1 | 0.0792 |
|  | deltoidea vs spicata | 0.0016 | 1 | 0.5149 |
|  | weberbaueri vs spicata | 0.0525 | 0.9703 | 0.4653 |
| lindeniana | duquei vs lindeniana | **0.5093** | 0.4554 | 0.3366 |
|  | duquei vs graminifolia | 0.0180 | 1 | 0.2772 |
|  | duquei vs concinna | **0.4422** | 0.2475 | 0.2178 |
|  | lindeniana vs graminifolia | 0.0167 | 1 | 0.6535 |
|  | lindeniana vs concinna | **0.6455** | 0.1287 | 0.0693 |
|  | graminifolia vs concinna | 0.0105 | 1 | 0.5446 |
| parvifolia | cogollo vs gloria | 0 | 1 | 1 |
|  | cogollo vs parvifolia | 0.0319 | 0.9802 | 0.4455 |
|  | cogollo vs tatama | 0 | 1 | 1 |
|  | cogollo vs bio | 0 | 1 | 1 |
|  | cogollo vs argos | **0.3005** | 0.7129 | 0.0594 |
|  | cogollo vs decipiens | 0.0115 | 1 | 0.2079 |
|  | gloria vs parvifolia | 0 | 1 | 1 |
|  | gloria vs tatama | 0 | 1 | 1 |
|  | gloria vs bio | **0.4460** | 0.4753 | 0.0099 |
|  | gloria vs argos | 0 | 1 | 1 |
|  | gloria vs decipiens | 0 | 1 | 1 |
|  | parvifolia vs tatama | 0 | 1 | 1 |
|  | parvifolia vs bio | 0 | 1 | 1 |
|  | parvifolia vs argos | 0.0158 | 1 | 0.5842 |
|  | parvifolia vs decipiens | 0.0525 | 0.9505 | 0.2079 |
|  | tatama vs bio | 0 | 1 | 1 |
|  | tatama vs argos | 0 | 1 | 1 |
|  | tatama vs decipiens | 0 | 1 | 1 |
|  | bio vs argos | 0 | 1 | 1 |
|  | bio vs decipiens | 0 | 1 | 1 |
|  | argos vs decipiens | 0.0019 | 1 | 0.3663 |
