# Supplementary material for: The Tracking of Moist Habitats Allowed Aiphanes (Arecaceae) to Cover the Elevation Gradient of the Northern Andes
Source: Front Plant Sci. 2022 Jun 27;13:881879. doi: 10.3389/fpls.2022.881879 (PMC9272002; doi:10.3389/fpls.2022.881879)

Supplementary Material

Supplementary Table 4. Table with age estimates and trees drawn in densitree from the SNAPP divergence time analysis of *Aiphanes concinna, A. lindeniana, A. linearis and A. hirsuta* (lindeniana and linearis complexes).

MUTATION RATE	2.6E-09					
**Runs 1 to 5 combined**	**Convergence checked!**					
	**95% CI**			**95% CI**		
**SPECIES**	**MIN**	**MEAN**	**MAX**	**my(MIN)**	**my (MEAN)**	**my (MAX)**
concinna - lindeniana	1554.62	277623.46	532484.23	0.00	0.28	0.53
concinna -lindeniana - linearis - hirsuta	1000122.31	1497810.77	2081635.38	1.00	1.50	2.08

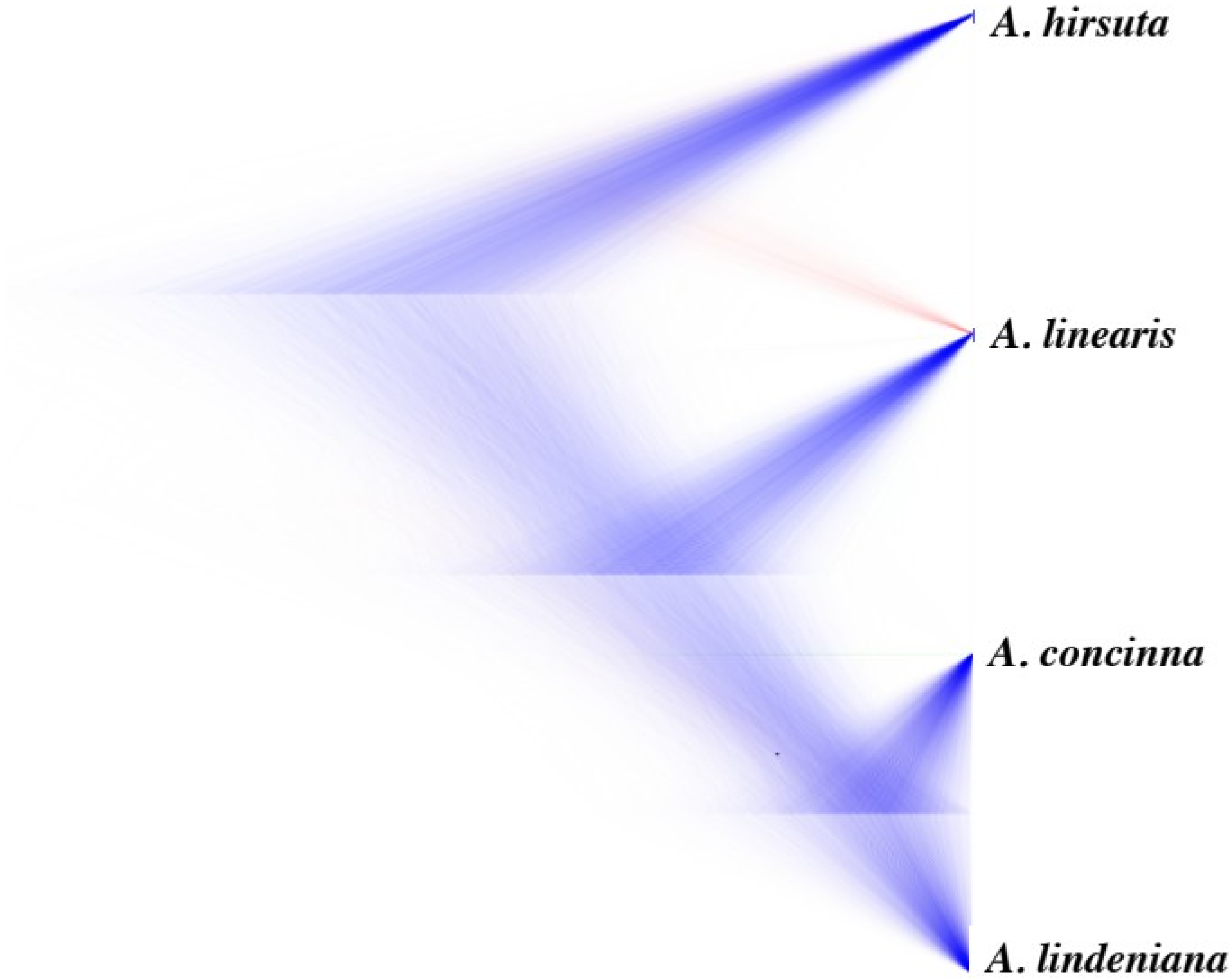

Supplement: Supplementary file 13 [file Table_4.DOCX]
